# Synthesis and Optical Properties of Unsymmetric Aromatically π‑Extended BODIPY

**DOI:** 10.1021/acs.joc.5c01432

**Published:** 2025-09-09

**Authors:** Metodej Dvoracek, Craig Newman, Mikhail Drobizhev, Brendan Twamley, Mathias O. Senge, Sergei A. Vinogradov, Mikhail A. Filatov

**Affiliations:** † School of Chemical and Biopharmaceutical Sciences, 8819 Technological University Dublin, City Campus, Grangegorman, Dublin D07 EWV4, Ireland; ‡ Department of Microbiology and Cell Biology, Montana State University, Bozeman, Montana 59717, United States; § School of Chemistry, Trinity College Dublin, The University of Dublin, Dublin 2 D02 R590, Ireland; ∥ Medicinal Chemistry, Trinity Translational Medicine Institute, St. James’s Hospital, Trinity College Dublin, The University of Dublin, Dublin D08 W9RT, Ireland; ⊥ Department of Biochemistry and Biophysics, Perelman School of Medicine, 6572 University of Pennsylvania, Philadelphia, Pennsylvania 19104, United States; # Department of Chemistry, School of Arts and Sciences, 6572 University of Pennsylvania, Philadelphia, Pennsylvania 19104, United States

## Abstract

A series of unsymmetrically substituted BODIPY dyes featuring fused benzo- or naphtho-fragments on one pyrrolic unit were synthesized from the corresponding pyrrolic precursors. The synthetic route was optimized using a modular approach based on the condensation of formylpyrroles with alkylpyrroles, enabling the identification of precursor combinations that minimize byproduct formation and improve preparative yields. The resulting benzo- and naphtho-fused BODIPYs display intense fluorescence in the red region, with emission maxima spanning 590–680 nm and fluorescence quantum yields ranging from 0.27 to 0.84. Their two-photon absorption (2PA) properties were studied both experimentally and computationally. An increase in the two-photon absorption cross-section with an increase in the size of the π-conjugated system was observed, reaching 80 GM for the naphthoBODIPY derivative. These findings demonstrate the potential of π-extended BODIPY scaffolds as NIR-active fluorophores with enhanced nonlinear optical properties.

## Graphical abstract

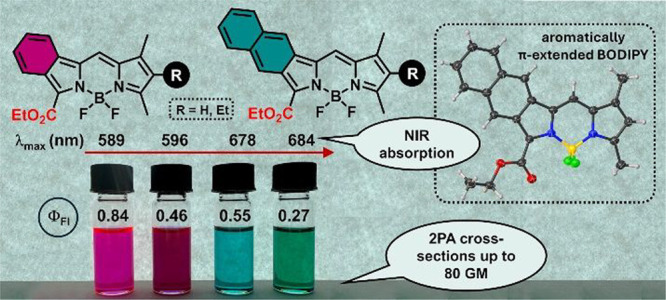

## Introduction

Dyes exhibiting absorption and/or emission in the red/near-infrared (NIR) region have garnered significant attention due to their diverse technological applications, such as dye-sensitized solar cells,^1,2^ photoemissive^3–5^ and photoacoustic probes,^6^ or photodynamic therapy (PDT) agents.^7^ The ability of red light to penetrate tissue efficiently makes red/NIR-absorbing dyes particularly valuable for medical imaging and therapeutic applications.^8^ As a result, interest in such dyes has grown rapidly in the past two decades.

For most dyes, absorption and emission characteristics are primarily governed by the HOMO–LUMO gap, which determines the energy of the electronic transitions involved. Structural modification of the chromophore allows for fine-tuning of this gap to achieve red-shifts in optical bands. Common strategies to modulate the HOMO–LUMO gap include extending π-conjugation, increasing molecular planarity to enhance orbital delocalization, and introducing charge-transfer character in the corresponding transitions.^9^

One prominent approach in the design of red/NIR dyes is based on using polymethine scaffolds, where electron-donating and electron-withdrawing groups are connected by sp^2^-hybridized carbon atoms, enabling charge delocalization over a larger molecular skeleton.^10^ Structural modification of the polymethine scaffold, such as the introduction of electron-donating/accepting groups or extension of the conjugated bridge, usually leads to red-shifts in absorption and emission and promotes intramolecular charge transfer. However, polymethine dyes typically exhibit rather low chemical stability, being highly susceptible to oxidation and various nucleophilic or electrophilic reactions, which limits their practicality in applications. Hence, alternative approaches to structural modification leading to efficient NIR absorption are of high interest.^11^

Boron dipyrromethene (BODIPY) dyes^12–14^ and their aza-substituted analogues^15,16^ have emerged as a versatile and attractive class of compounds with potential applications in bioimaging,^17^ photodynamic therapy (PDT),^18^ triplet–triplet annihilation upconversion,^19^ and photocatalysis.^20^ BODIPYs are characterized by excellent photostability, high molar absorption coefficients, sharp and intense fluorescence, and convenient synthetic accessibility. They are also highly amenable to diverse structural modifications, enabling fine-tuning of their optical and physicochemical properties.

Several approaches are available for inducing bathochromic shifts in the BODIPY optical spectra, such as extension of conjugation or fusion of aromatic rings to the core,^21^ introduction of electron-donating substituents,^22^ B–O chelation,^23^ rigidification of the conjugated backbone by annelation,^24^ oligomerization,^25^ introduction of sulfur atoms into the aromatic systems,^26^ and formation of aggregates.^27^ A combination of these approaches can lead to highly red-shifted absorption maxima, potentially up to 1000 nm.^28^

BODIPYs are also being investigated for their two-photon absorption (2PA) properties, which enable a range of nonlinear optical applications, including 3D microfabrication,^29^ optical data storage,^30^ optical power limiting,^31^ two-photon microscopy,^32^ and two-photon photodynamic therapy.^33^ 2PA occurs when a molecule simultaneously absorbs two photons, leading to excitation to a higher energy state. Structure–property relationships could be an effective tool for designing chromophores exhibiting large 2PA.^34^ Achieving strong 2PA requires careful structural tuning, where approaches similar to those used for linear absorption tuning can be applied. In particular, incorporating donor–acceptor (D-A) motifs may enhance 2PA by promoting intramolecular charge transfer (ICT).^31^ This strategy has recently been employed to develop BODIPY-based optical probes^35^ and theranostic agents,^36,37^ though the synthesis of such D–A systems is often demanding and can lead to large, hydrophobic structures with limited biomedical applicability.

This work focuses on the synthesis and characterization of two types of π-extended BODIPY dyes: benzo- and naphtho-BODIPY. The dyes were designed having an unsymmetric structure, where one pyrrolic unit is modified with an electron-donating alkyl groups and the other with a net electron-withdrawing fused aromatic ring and an alkoxycarbonyl group (Figure 1). The unsymmetric distribution of charge in these structures can lead to a more pronounced difference between the static dipole moments in the ground and excited states, which could lead to an enhancement of two-photon absorption cross sections.^38^ Previous studies have shown that unsymmetrical substitution significantly alters the photophysical properties of BODIPY derivatives, including fluorescence quantum yields and lifetimes.^39^ Notably, unsymmetrical substitution significantly enhances singlet oxygen photosensitization efficiency compared with symmetric BODIPYs. In particular, introduction of a single ethoxycarbonyl group on one of the pyrrolic rings of the BODIPY core led to efficient S_1_ → T_2_ intersystem crossing (ISC), facilitated by a reduced singlet–triplet energy gap (Δ*E*_S–T_).^40^ Importantly, unsymmetric BODIPYs possess relatively compact structures, as compared with many other NIR BODIPY dyes, which make them potentially practical as fluorophores for translational development of biological imaging probes.

**Figure 1:**
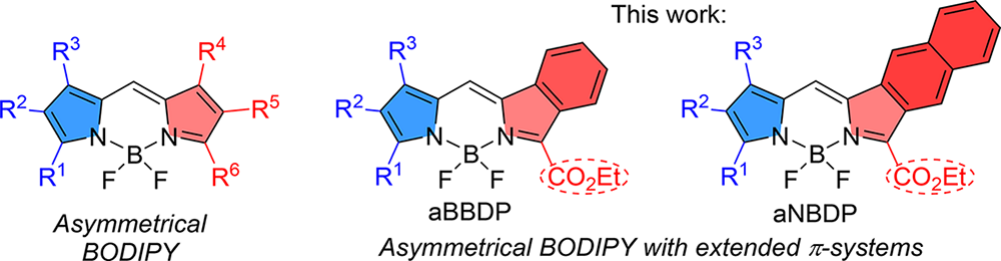
Structures of the target unsymmetric benzo- and naphthoBODIPY (**aBBDP** and **aNBDP**, respectively).

Here, we report the synthesis of unsymmetric BODIPYs with extended π-conjugation systems (**aBBDP** and **aNBDP**, Figure 1) and investigate their NIR optical properties, focusing on their two-photon absorption. We show that π-extension and unsymmetrical substitution enhance 2PA to the lowest energy excited states and, more importantly, stabilize higher-lying strongly 2P-active excited states. Both computational and experimental data reveal a red-shift in the 2PA band with increasing π conjugation, suggesting that our approach could be used to manipulate 2PA in BODIPY dyes.

## Results and Discussion

### Synthesis of Target Compounds

Based on the molecular design outlined above, we focused on the synthesis and characterization of the newly designed BODIPY derivatives, **aBBDP-1** and **aNBDP-1**, featuring extended π-systems through the attachment of benzo- and naphtho-rings to one of the pyrrolic rings of the BODIPY core (Figure 1). The rationale for this modification lies in its potential to reduce the HOMO–LUMO gap, leading to a red-shift in the absorption spectrum, extending into the near-infrared NIR region. The introduction of asymmetry in the substitution pattern—attaching the benzo- or naphtho-rings to only one pyrrolic ring—distinguishes this work from the prevalent focus on symmetric BODIPY derivatives, such as dibenzo-^41^ and distyryl-substituted BODIPYs.^42^ In the latter, symmetric substitution results in a more uniform electronic density distribution over the chromophore structure. In contrast, the unsymmetric attachment of the π-extended groups in the current study is expected to desymmetrize the electronic structure, which can significantly impact the photophysical properties, particularly 2PA. This effect has been observed in similar unsymmetrically π-extended porphyrins, where breaking the molecular symmetry increases the 2PA cross-section to the lowest energy states.^43^

Based on the retrosynthetic analysis presented in Scheme 1, there are two potential synthetic routes for the preparation of the target compounds. While some isoindoles bearing electron-withdrawing groups are sufficiently stable,^44^ most substituted isoindoles lack the necessary stability, precluding their direct use in synthesis.^45^ To overcome this limitation, either synthetic equivalents must be employed or alternative methods developed to introduce fused aromatic rings into the target structures. We opted to use synthetic equivalents, which offer improved stability while retaining key reactivity features of pyrroles, enabling their use as precursors in the synthetic strategy outlined in Scheme 1.

**Scheme 1:**
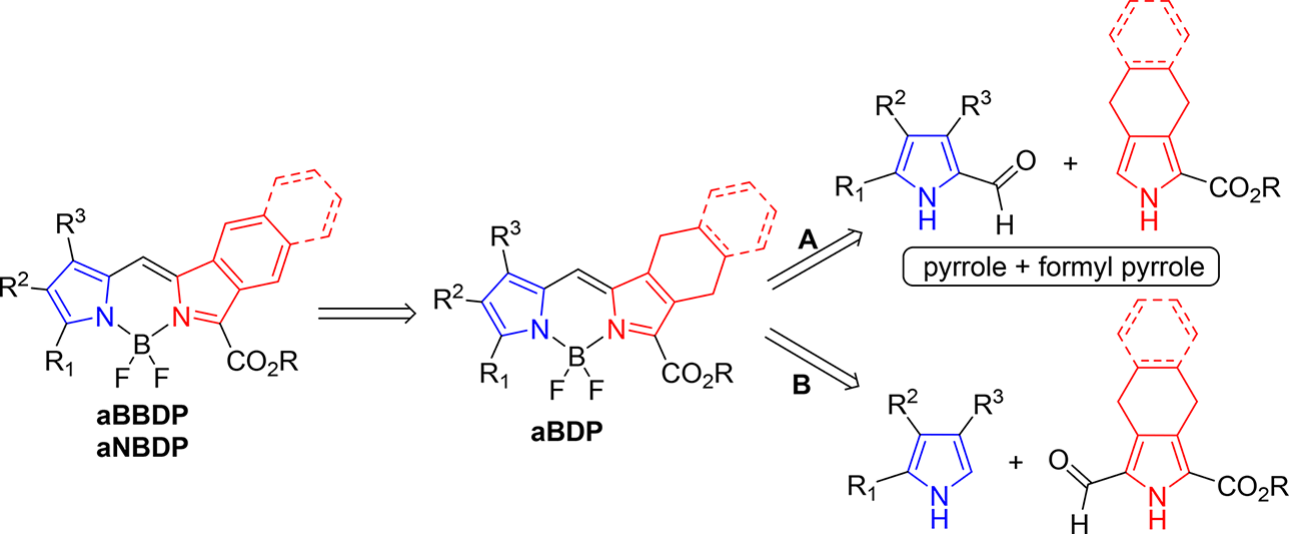
Synthetic Approach toward Unsymmetric BODIPYs

Several approaches to the synthesis of π-extended BODIPY derivatives have been reported in the literature.^21c^ In this study, we adopted a strategy involving the oxidative aromatization^46^ of tetrahydro- or dihydro-isoindole precursors, a method previously developed for the synthesis of π-extended porphyrins.^47^ As outlined in Scheme 1, both synthetic routes begin with the condensation of an α-unsubstituted pyrrole with a suitable formylpyrrole. ‘Masked’ isoindole precursors, specifically 5,6,7,8-tetrahydroisoindole and 4,9-dihydrobenzoisoindole derivatives, were used as stable synthetic equivalents of otherwise unstable isoindole and benzoisoindole derivatives. This strategy relies on the assumption that the target π-extended BODIPY derivatives are stable. Thus, the masked fragments can be safely incorporated into the BODIPY framework during synthesis and subsequently ‘unmasked’ via oxidative aromatization using reagents such as DDQ, affording the corresponding benzo- and naphtho-extended BODIPY derivatives.

The key distinction between these routes lies in the placement of the formyl group on the pyrrole ring. In route A, the formyl group is introduced into the alkylpyrrole precursor, which is subsequently reacted with an alkoxycarbonyl pyrrole bearing a single unsubstituted α-position. In route B, the formyl group is introduced into the alkoxycarbonyl pyrrole, which is then reacted with an appropriate alkylpyrrole. These formylpyrroles are readily accessible via the Vilsmeier reaction.^48^ In this work, we have explored both synthetic routes to evaluate their relative efficiency, yield, and selectivity. The comparison of these two approaches provides insight into the most effective and scalable method for preparing the desired unsymmetric BODIPY derivatives.

The pyrrolic precursors selected to provide the necessary building blocks for the synthesis of the target BODIPY derivatives are depicted in Figure 2. These include 1*H*-pyrrole (**P1**), 2,4-dimethylpyrrole and 3-ethyl-2,4-dimethylpyrrole (**P2** and **P3**, respectively), as well as derivatives of 4,5,6,7-tetrahydro-2*H*-isoindole^49^ (**P4** and **P6**) and 4,9-dihydro-2*H*-benzo­[*f*]­isoindole benzoisoindole^50^ (**P5** and **P7**, respectively). Compounds **P6** and **P7** were synthesized from **P4** and **P5**, respectively, by the reduction of the ethoxycarbonyl group using LiAlH_4_.^51^ These pyrrole precursors were then successfully converted into the corresponding formylpyrroles via the Vilsmeier reaction using a literature procedure.^52^

**Figure 2:**
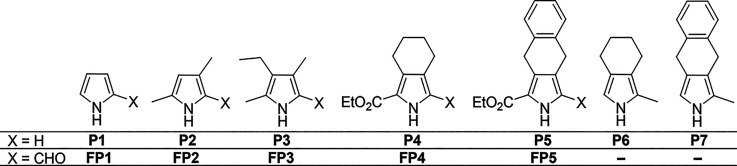
Structures of starting pyrroles and formylpyrrole compounds.

BODIPYs containing “masked″ isoindole and benzoisoindole fragments (**aBDP 1–6**) were synthesized via the condensation of various pyrroles with formylpyrroles according to routes A and B (Scheme 2). The reaction involved treating a pyrrole and formylpyrrole mixture with phosphorus oxychloride, leading to the formation of the corresponding dipyrromethenes.^53^ These intermediates were not isolated but were directly converted into BODIPYs by treatment with boron trifluoride etherate in the presence of triethylamine. In both cases, the condensation resulted in the formation of the target products, along with symmetric byproducts, including 1,3,5,7-tetramethyl-BODIPY (**sBDP-1**) and 2,6-diethyl-1,3,5,7-tetramethyl-BODIPY (**sBDP-2**).

**Scheme 2:**
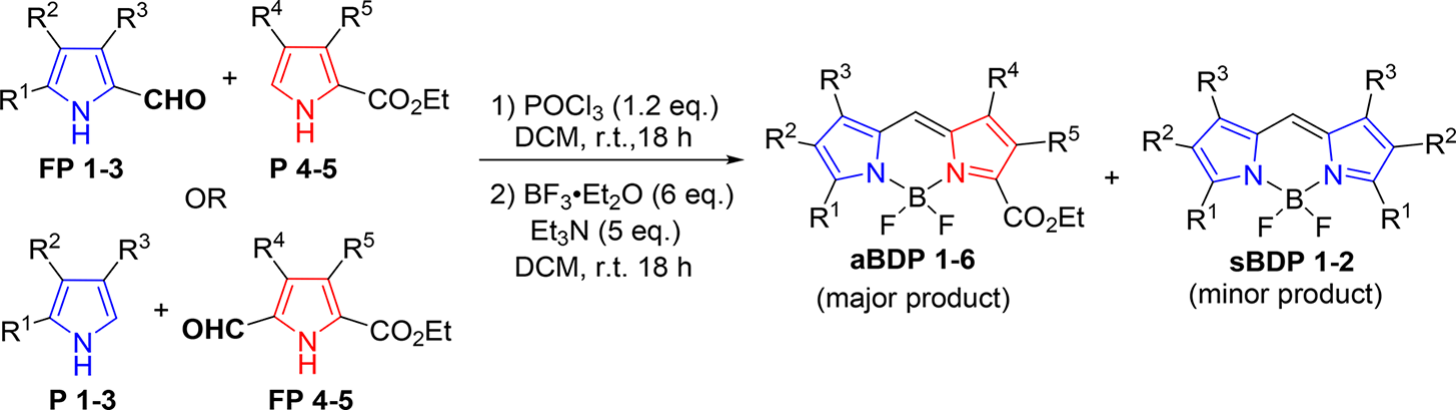
Condensation of Pyrroles with Formylpyrroles

As can be seen from Table 1, in most cases, route A led to a higher yield of these byproducts, compared to route B, which gave a higher yield of the target **aBDP 1–6**. The products were purified by column chromatography, which in some cases proved challenging due to the presence of byproducts. In some instances, the obtained **aBDP**s required further purification through preparative TLC or using a second silica gel column for the isolation of the target compounds in pure form. This led to reduced overall yields of the target products, as observed, for example, in the cases of **aBDP 5** and **6**. However, in general, the synthetic procedure used in this work is relatively efficient compared to previous reports on pyrrole–formylpyrrole condensation, which usually gives a yield range of 20–50%.^54^ Notably, much higher yields were obtained in the cases of **aBDP 2** and **4** (75 and 85%, respectively), which demonstrates the efficacy of the approach used. The improved selectivity observed for methodology B is likely due to the higher electrophilicity of the formyl group in pyrrolic substrates bearing an ethoxycarbonyl substituent, which may facilitate faster and more efficient condensation, thereby reducing the extent of byproduct formation.

**Table 1:**
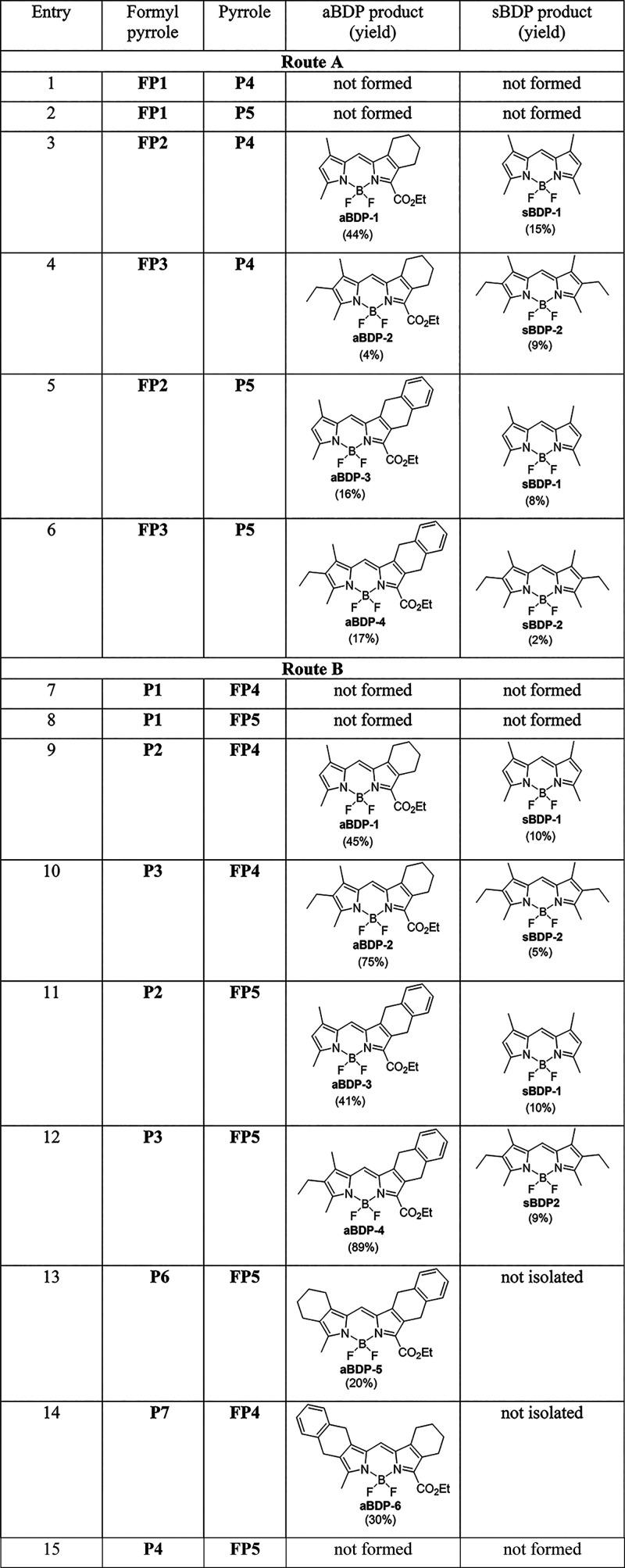
Starting Materials Used for the Synthesis of **aBDP 1–6** and Isolated Byproducts

The formation of symmetric byproducts during the synthesis of asymmetrical BODIPYs has been previously reported in the works of Ortiz and co-workers^54^ and Wu and Burgess.^55^ In the latter study, it was shown that phosphorus oxychloride can promote the self-condensation of pyrrole-2-carbaldehyde, leading to the formation of symmetric alkyl BODIPYs. The proposed mechanism involves substitution of the aldehyde oxygen phosphorus oxychloride, generating a chlorinated azafulvene intermediate. This species is then attacked by a second pyrrole-2-carbaldehyde molecule, followed by a nucleophilic attack by chloride. Decomposition of the resulting unstable intermediate yields the corresponding dipyrromethene (Scheme 3).

**Scheme 3:**
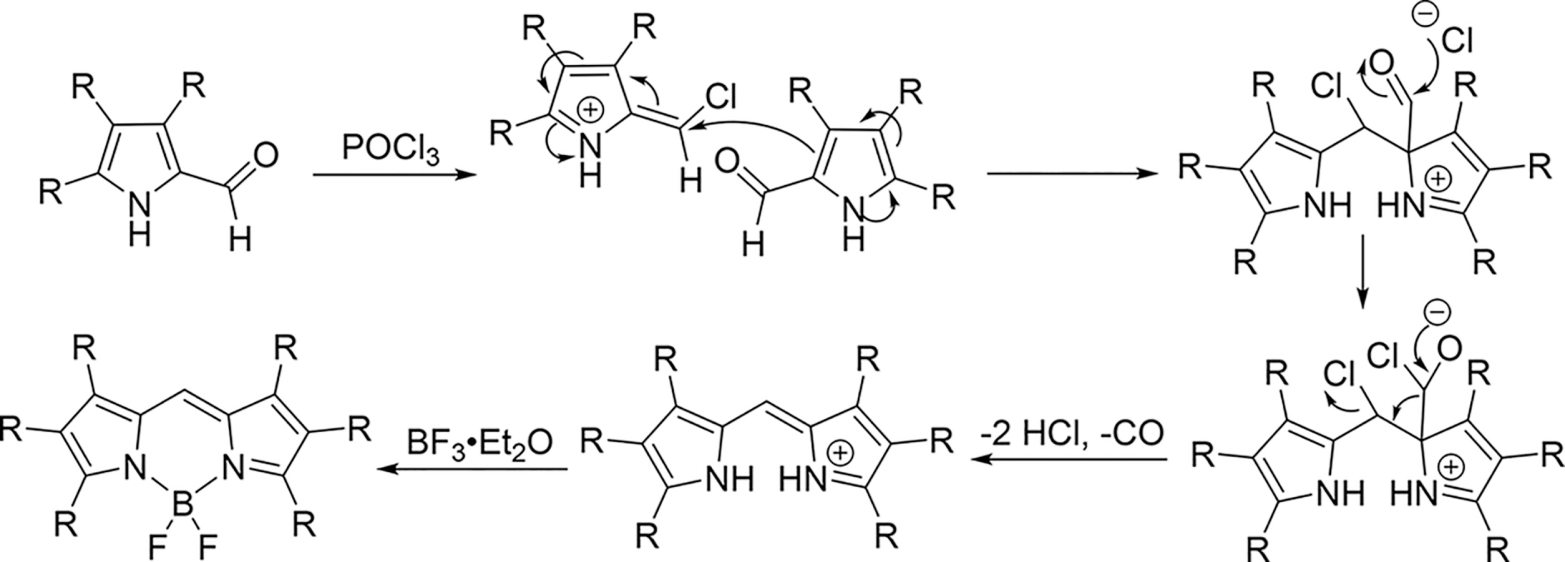
Proposed Mechanism for the Formation of Symmetric BODIPY Byproducts

As shown in Table 1, in certain cases, the condensation of pyrrole aldehyde with pyrroles did not yield the target BODIPY products. For example, attempts to synthesize the target BODIPY containing an alkyl-unsubstituted pyrrole fragment were unsuccessful. The same synthetic procedure was initially applied, where pyrrole and formylpyrrole reagents, such as **FP1** and **P4**, were reacted with POCl_3_, followed by treatment of the reaction mixture with BF_3_·Et_2_O and Et_3_N. However, in these attempts (entries 1–2, Table 1), no BODIPY formation was observed.

Subsequent attempts were made using alternative procedures, including increasing the amount of catalyst, using other catalysts such as hydrogen bromide and TFA, and extending the reaction time up to 36 h; however, these attempts were unsuccessful. Later, neat 1*H*-pyrrole was used as a solvent for reactions with **FP4** and **FP5** (entries 7 and 8, Table 1) instead of DCM. However, none of these modifications resulted in the formation of the target dipyrromethene. The starting compounds **FP4** and **FP5** were isolated unreacted from these mixtures, and UV–vis and TLC analyses of the resulting reaction mixtures showed no evidence of any BODIPY products. These results are likely due to the substantially lower nucleophilicity of 1H-pyrrole compared with the more electron-rich substituted pyrroles **P2** and **P3**, which reacted successfully under the same conditions. Additionally, the presence of multiple CH positions in 1H-pyrrole and its derivatives may enable alternative reaction pathways, leading to undesired side products.

Additionally, unsuccessful attempts were recorded when trying to react compounds **P4** and **FP5** to obtain the corresponding unsymmetric dialkoxycarbonyl BODIPY, using POCl_3_ as a catalyst. The reaction of **P4** with **FP5** showed consumption of the starting materials, which indicated the formation of the intermediate dipyrrin compound. However, subsequent treatment with BF_3_·Et_2_O and Et_3_N did not yield the desired asymmetric BODIPY product. Uppal et al. reported that the reaction of the corresponding dialkoxydipyrrins with BF_3_·Et_2_O and Et_3_N required heating at reflux in toluene to afford the corresponding BODIPYs,^56^ implying that steric hindrance from α-substituents may hinder boron insertion. Following this rationale, attempts were made to accelerate complexation of boron under various conditions, including (1) heating at 80 °C for 12–24 h in toluene or 1,2-dichloroethane (DCE), (2) refluxing in the same solvents, and (3) refluxing in DCM under microwave irradiation, as described by Shambalova et al.^57^ Nonetheless, none of these conditions yielded the desired BODIPY, and no product formation was detected in the reaction mixtures.

Scheme 4 illustrates the conversion of a series of prepared **aBDP** precursors into **aBBDP** 1–2 and **aNBDP** 1–2 through an oxidative aromatization reaction. In a typical reaction, the selected **aBDP** compound was refluxed in toluene with an excess of DDQ; 3 equiv for benzoBODIPY and 1.5 equiv for naphthoBODIPY. During the reaction, a noticeable color change occurred: from dark red/purple to vibrant magenta for the benzoBODIPY reaction mixtures, and from dark black/green to vibrant emerald for the naphthoBODIPY reaction mixtures. The progress of the reaction was monitored by TLC, with reaction times ranging from 30 to 60 min. The crude products were then purified on silica columns packed with DCM, using pure DCM as the eluent, and characterized by NMR and high-resolution mass spectrometry (Supporting Information). The yields obtained, ranging from 33 to 74%, are generally consistent with those reported for the aromatization reactions of corresponding porphyrins.^58^

**Scheme 4:**
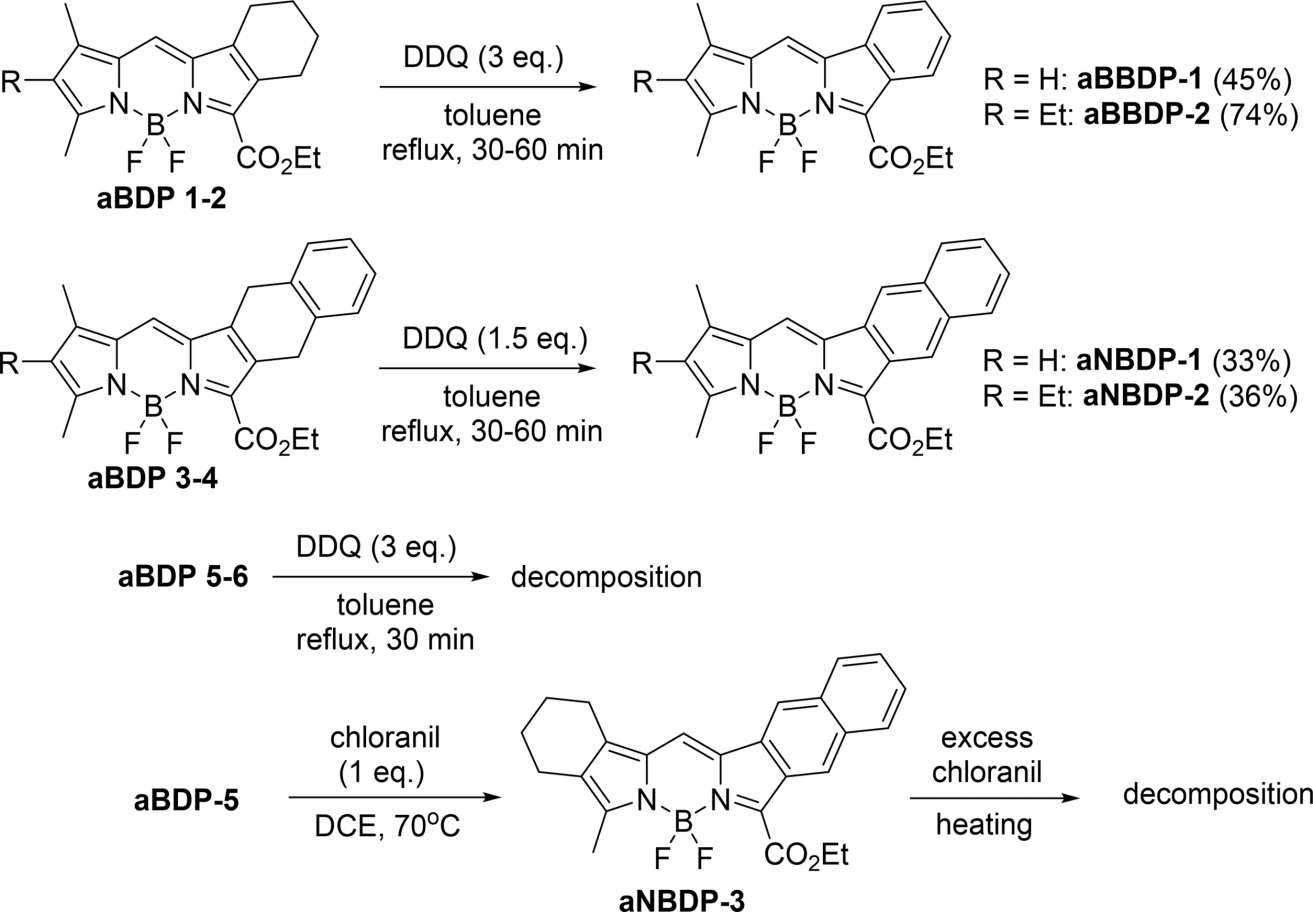
Aromatization of **aBDP**s

In the attempted aromatization of compounds **aBDP**-5 and **aBDP**-**6**, 4.5 equiv of DDQ were used to account for the additional rings in these structures that require aromatization. However, after refluxing the corresponding starting compounds with DDQ for 30 min, a color change to dark brown was observed, and no BODIPY products were detected by TLC or UV–vis analysis. Several attempts were made to optimize the reaction conditions, including varying solvents and reaction temperatures. Although the products formed in these reactions could not be identified, it is likely that the target compounds are not sufficiently stable and undergo decomposition, which is consistent with the known instability of isoindoles. In an attempt to carry out the reaction under milder conditions, **aBDP**-5 was reacted with 1 equiv of chloranil at 70 °C, resulting in the formation of the corresponding naphthoBODIPY **aNBDP**-3 (Scheme 4). However, further attempts to convert **aNBDP**-3 into a fully aromatized BODIPY with excess DDQ or chloranil led to decomposition. UV–vis analysis showed the complete disappearance of the characteristic BODIPY absorption bands, confirming that the reaction had resulted in decomposition. The structures of the products formed in this reaction could not be determined.

Single-crystal X-ray diffraction data were obtained for two BODIPY derivatives, **aBDP-1** and a**NBDP-1** (Figure 3). Details of structure determination are given in the Supporting Information.^59^ In **aBDP-1**, a face-to-face packing mode is observed. However, conformational flexibility of the cyclohexane moiety leads to disorder within the crystal, resulting in a 12:88 occupancy ratio between the two conformers. Intramolecular hydrogen bonding is present, specifically F1···H12C at a distance of 2.41209(10) Å. Analyzing the planarity of the BODIPY core showed minimal atomic displacement from the mean plane, with a root-mean-square distance (RMSD) value of 0.012 Å. The carbonyl group is not coplanar with the BODIPY core in either structure, with twist and fold angles of 14.0557(9)° and 129.116(2)°, respectively. **aNBDP-1** exhibits a similar face-to-face packing motif, with intermolecular hydrogen bonding contributing to crystal packing. Two notable interactions are observed: C12–H12···F2 at 2.47(8) Å and C6–H6···F2 at 2.7008(2) Å, forming a network in the crystal. The BODIPY core of **aNBDP-1** exhibits a slightly greater deviation from planarity in the BODIPY core than in **aBDP-1**, with an RMSD of 0.021 Å. However, the carbonyl group is more coplanar, with twist and fold angles of 10.2963(13)° and 5.5407(6)°, respectively. This indicates less deviation than that in **aBDP-1**, with angles closer to planarity. When the carbonyl group is included in the mean plane calculation of the BODIPY core, the overall RMSD is 0.098 Å for **aBDP-1** and 0.083 Å for **aNBDP-1**.

**Figure 3:**
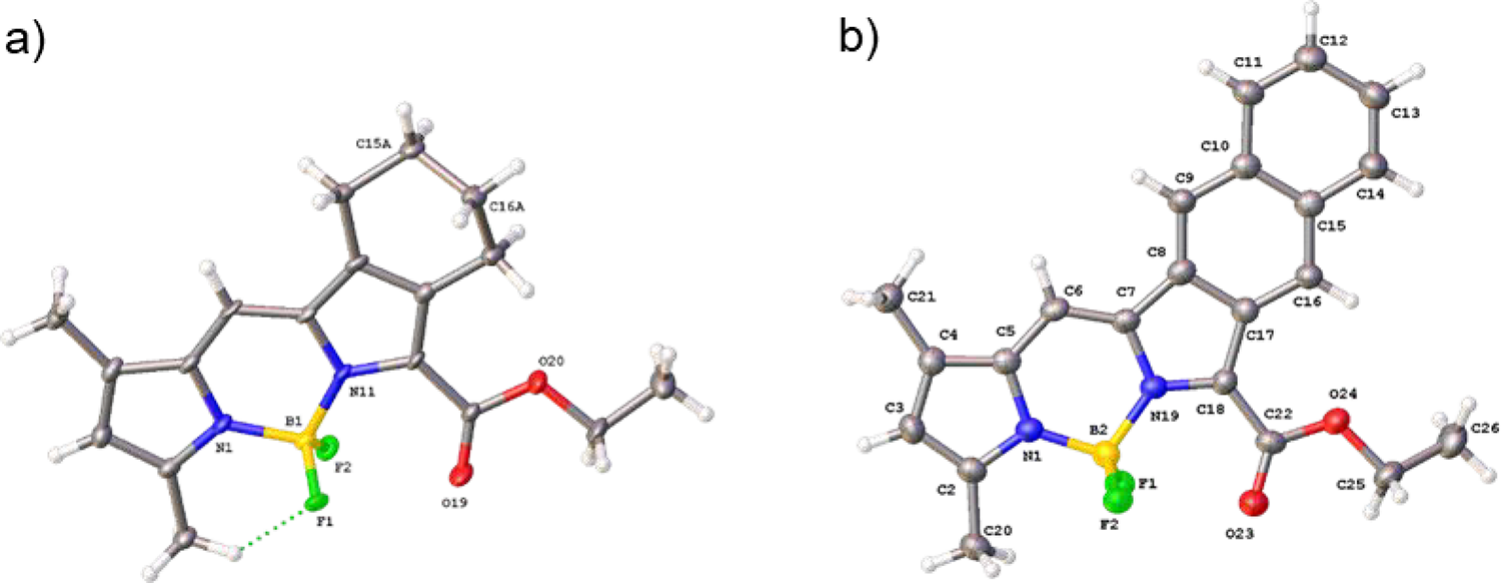
View of the molecular structures of **aBDP-1** (a) and **aNBDP-1** (b) in the crystal. Atomic displacement is shown at 50% probability.

Analysis of an **aNBDP-1** crystal obtained from toluene (**Tol**) revealed disorder in the terminal carbon of the ethoxycarbonyl group and the terminal ring of the naphto moiety. In this solvated form, the BODIPY core exhibits complete planarity, with an RMSD value of 0.000 Å. The carboxy group is coplanar to the BODIPY core, with no fold angle and a twist angle of 180°. When the carbonyl group is included in the mean plate calculation, the overall RMSD remains at 0.000 Å, further confirming the planarity of **aNBDP-1-Tol**.

Previously reported crystal structures of related BODIPY derivatives, which can be found in the Cambridge Crystallographic Data Centre, provide useful context for understanding the structural features observed here. A symmetric dinaphthoBODIPY reported by Yamazawa et al. exhibits significantly lower planarity of the BODIPY core compared to the structures in this work, with an RMSD of 0.104 Å.^60^ Several symmetric benzoBODIPY compounds, reported by Uppal et al.,^56^ also illustrate trends in planarity and coplanarity of the carbonyl group with respect to the BODIPY core. For example, SAVGON (Figure S20)—structurally analogous to **aBBDP-2**, but bearing a methoxycarbonyl substituent—shows a BODIPY core RMSD of 0.020 Å, similar to that of **aNBDP-1**. However, the carboxy group is less coplanar to the core, with a twist angle of 7.413486° and a fold angle of 130.048925°. Additional structures, SAVFIG and SAVGAZ, serve as symmetric references to the compounds in this study. The BODIPY core of SAVFIG has an RMSD of 0.070 Å, compared to 0.054 Å for SAVGAZ, showing increased planarity after aromatization of the cyclohexane moieties, similar to what was observed with **aBDP-1** and **aNDBP-1**.

### Optical Properties

The optical properties of the new compounds, including the precursor BODIPY **aBDP 1–6** with partially saturated rings, as well as the unsymmetric **aBBDP**s and **aNBDP**s, were investigated. The precursor BODIPYs were used as reference compounds to compare the effects of structural modifications on absorption, emission, fluorescence lifetimes, quantum yields, singlet oxygen generation, and two-photon absorption properties. The absorption and emission properties were investigated in dichloromethane, toluene, and acetonitrile, and the results are summarized in Tables 2 and Figure 4, with additional details in the Supporting Information (Figure S13).

**Figure 4:**
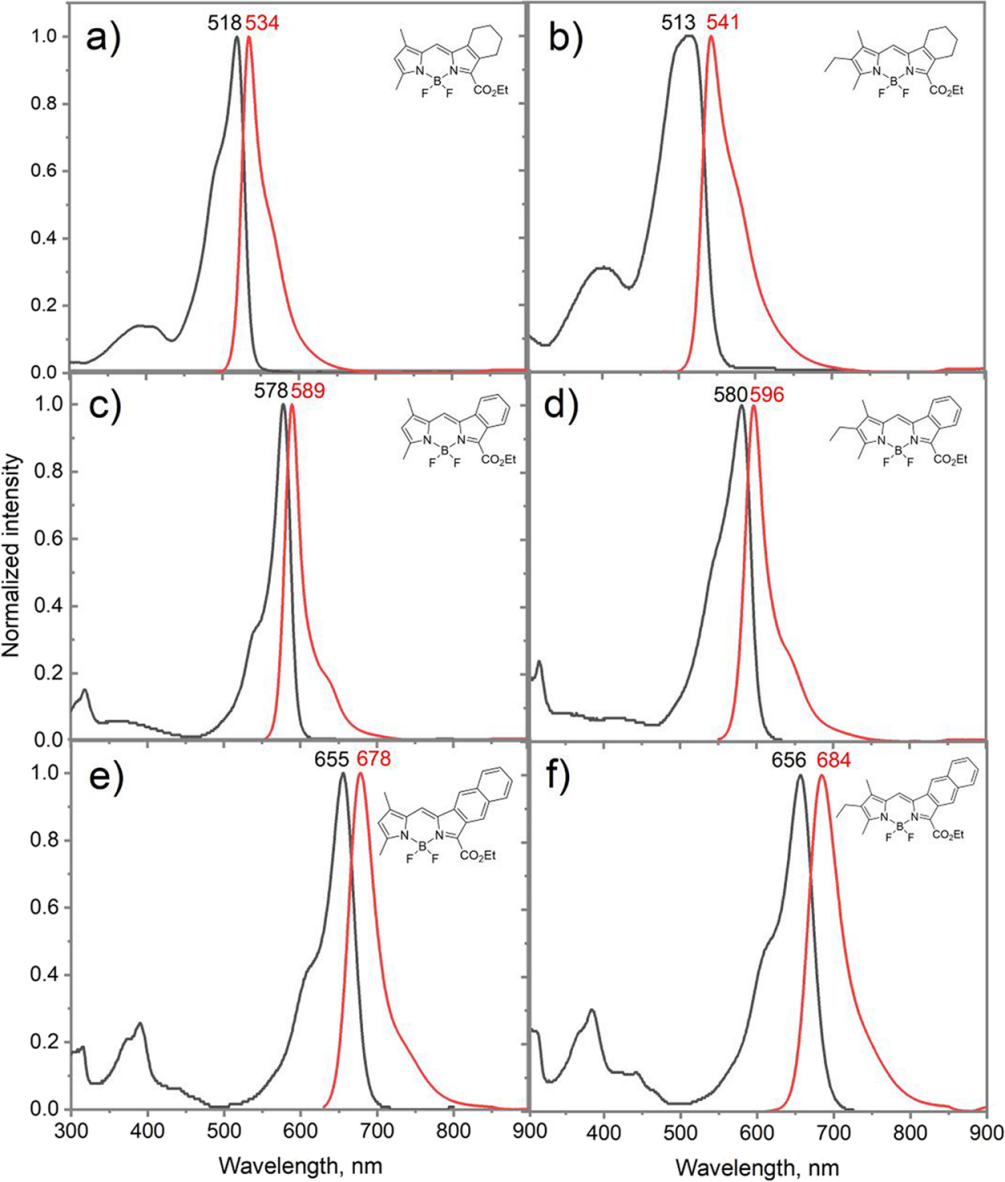
Comparison of optical spectra of the synthesized **aBDP** (a,b), **aBBDP** (c,d), and **aNBDP** (e,f) compounds in DCM.

**Table 2:** Optical Properties of the Compounds Studied^a^

compound	absorption λ_max_ (nm)^a^	ε, M^–1^ cm^–1^ × 10^4^ ^a^	fluorescence λ_max_ (nm)^a^	Φ_F_ ^a^ ^,^ ^b^	τ, ns	Φ_Δ_ ^c^	σ^2^_max_, GM^d^ (λ_2P_, nm)
**aBDP-1**	518	4.71	534	0.68	5.46	0.06	8.0 (684)
							12 (968)
**aBDP-2**	513	2.98	542	0.26	3.55	0.18	10 (680)
							9.3 (972)
**aBDP-3**	516	4.68	532	0.70	5.05	0.06	7.9 (680)
							9.2 (956)
**aBDP-4**	500	3.85	541	0.18	2.725	0.18	11 (680)
							11 (968)
**aBDP-5**	504	2.18	539	0.19	2.91	0.20	8.7 (680)
							8.2 (972)
**aBDP-6**	521	2.12	540	0.32	4.752	0.08	7.3 (680)
							4.5 (980)
**aBBDP-1**	578	8.43	589	0.84	6.16	0.02	23 (740)
							20 (1072)
**aBBDP-2**	580	5.67	596	0.46	3.88	0.04	15 (740)
							12 (1092)
**aNBDP-1**	655	6.04	678	0.55	6.635	n.d.^e^	67 (840)
							16 (1212)
**aNBDP-2**	657	6.11	684	0.27	3.775	n.d.^e^	83 (860)
							44 (1240)

a Measured in DCM. Additional UV–vis absorption, and fluorescence emission spectra and data measured in acetonitrile and toluene are reported in the Supporting Information.

b Measured using Rhodamine 6G as a standard (Φ_F_ = 0.95 in EtOH), and aBBDP-1 (Φ_F_ = 0.846 in acetonitrile) as a standard for aBBDP-1–2 and aNBDP-1–2.^64^

c Measured in toluene using 1,9-dimethylanthracene as a singlet oxygen sensor and 2,6-diiodo-8-phenylBODIPY (Φ_Δ_ = 0.85)^65^ and methylene blue (Φ_Δ_ = 0.51)^66^ as reference photosensitizers using a literature method.^67^

d Maximum 2PA cross-section measured in d^6^-DMSO in two different spectral regions. The first region (upper line) corresponds to a shorter wavelength transition (S_0_ → S_n_), and the second region (lower line) corresponds to a vibronic peak of the first electronic transition (S_0,0_ → S_1,1_).

e n.d.: not detected.

By comparing the emission and absorption spectra of the precursor **aBDP** compounds and the aromatized BODIPY products, it can be seen that the obtained π-extended BODIPYs exhibit red-shifted absorption and emission bands, with a shift of 50–70 nm per benzo-annulated ring. Figures 4 shows the normalized absorption and fluorescence spectra of **aBDPs 1–2**, benzoBODIPYs **aBBDP 1–2**, and naphthoBODIPYs **aNBDP 1–2** in dichloromethane.

**aBDP**s exhibit absorption bands in the range of 500–520 nm and emission bands in the 530–540 nm range, with a notable dependence on the substitution pattern. These spectra show full width at half-maximum (fwhm) values significantly larger than those observed for typical symmetric BODIPYs.^12^ These compounds also exhibit solvatochromic behavior (Figure S13), showing hypochromic shifts with an increase in solvent polarity. This effect was more pronounced for derivatives containing the 3-ethyl-2,4-dimethylpyrrole unit. For instance, compound **aBDP-2** displayed a larger Stokes shift (29 nm) compared to that of **aBDP-1** (16 nm) and a reduced molar extinction coefficient. At the same time, the emission quantum yields of **aBDP**s **2**, **4**, **5**, and **6**, which contain trialkyl-substituted pyrrolic groups, were reduced compared to **aBDP**s **1** and **3**, which feature dimethylpyrrole fragments. Fluorescence quantum yields for **aBDP-1** and **aBDP-3** (Φ_F_ = 0.68–0.7) were significantly higher than those for **aBDP**s **2**, **4**, **5**, and **6** (Φ_F_ = 0.18–0.32), which could be a result of a stronger donor–acceptor character in the latter chromophores due to the presence of an extra electron-donating group (ethyl) in the structures. The resulting asymmetric charge distribution could underpin enhancement of nonradiative decay pathways.^61^ A more polar nature of **aBDP**s **2**, **4**, **5**, and **6** could also be responsible for lower molar extinction coefficients of these compounds.

As expected, the annelation of benzo rings induced significant bathochromic shifts in both the absorption and emission bands, with a shift of 50–70 nm per ring across all solvents. For example, the aromatization of **aBDP**-1 to form the monobenzo-BODIPY **aBBDP**-1 resulted in an ∼60 nm red-shift. The incorporation of a naphthalene fragment in **aNBDP**-1 caused an approximate 140 nm red-shift (Table 2) relative to the parent compound **aBDP**-3. Even larger bathochromic shifts were observed for BODIPYs containing the 3-ethyl-2,4-dimethylpyrrolic fragment, with a 160 nm shift in absorption upon conversion of **aBDP**-4 to **aNBDP**-2.

Fluorescence quantum yields in dichloromethane were generally higher for compounds containing dialkyl-substituted pyrrole fragments compared to those with trialkyl-substituted ones. The quantum yields typically decreased with the extension of the π-system, dropping from 0.84 for **aBBDP**-1 to 0.55 for **aNBDP**-1. This reduction is likely due to the enhancement of nonradiative relaxation accompanying the red-shift, in accordance with the Strickler–Berg law, leading to a decrease in the excited-state lifetimes. Compounds **aBBDP**-2 and **aNBDP**-2 exhibited shorter lifetimes (3.88 and 3.78 ns, respectively) compared to **aBBDP**-1 and **aNBDP**-1 (6.16 and 6.64 ns, respectively). Solvent polarity also influences fluorescence quantum yield, with the impact depending on the structure. **aBBDP**-1, shows the lowest solvent dependence, maintaining high quantum yields across all solvents −0.84 in acetonitrile and dichloromethane and 0.86 in toluene. In contrast, **aBBDP**-2 exhibits the strongest solvent dependence, with quantum yields varying from 0.38 in acetonitrile to 0.62 in toluene. **aBBDP**-1 has the highest fluorescence quantum yield among the compounds studied in this work (0.84–0.86). Optical properties of all compounds in acetonitrile and toluene are presented in the Supporting Information (Figure S13, Tables S1 and S2).

High quantum yields of red-absorbing BODIPY derivatives have been reported in the past. For example, Uppal et al. reported high quantum yield values of 0.99 and 0.77 for an unsymmetric benzoBODIPY of 0.99 and 0.77 in dichloromethane and methanol, respectively.^56^ Analogues of **aBBDP**-1 with halogen substituents at the 3-position (Cl or Br), reported by Jiao et al., display quantum yields of 0.62–0.665 in dichloromethane.^62^ While no unsymmetric [*a*]-naphthoBODIPYs have been reported to date, Yamazawa et al. described the photophysical properties of symmetric dinaphthoBODIPYs, which exhibited fluorescence quantum yields ranging from 0.16 to 0.29.^60^

The singlet oxygen quantum yields (Φ_Δ_) (Table 2) were determined using both a direct method based on NIR phosphorescence (see Supporting Information) and an indirect method employing 1,9-dimethylanthracene as a chemical trap for singlet oxygen. Despite the absence of heavy atoms, some of the studied BODIPYs were found to generate singlet oxygen, with quantum yields as high as 20% observed for **aBDP**-5. Φ_Δ_ values were found to be solvent-dependent; however, no consistent trend was observed with respect to solvent polarity (Table S3). Singlet oxygen generation was enhanced for compounds containing electron-rich trialkylpyrrolic fragments (**aBDP** 2, 4, 5, and 6), which correlates with the lower fluorescence quantum yields observed for these compounds. No phosphorescence was detected for these BODIPYs, which are generally known for their nonemissive triplet state, but the generation of singlet oxygen suggests the involvement of the BODIPY triplet state formed through intersystem crossing.^63^ For **aBDP**-1 and **aBDP**-2, this enhanced intersystem crossing was previously attributed to an S_1_-T_2_ pathway facilitated by a reduced singlet–triplet energy gap (Δ*E*_S‑T_).^40^ Given the structural similarities of the other compounds in this series (**aBDP** 3–6), this mechanism is likely to apply to those compounds as well. Interestingly, no singlet oxygen generation was observed for the studied π-extended BODIPYs, as indicated by the Φ_Δ_ values in Table 2 and the IR phosphorescence spectra (Figure S15). This suggests that for **aBBDP 1–2** and **aNBDP 1–2**, this ISC mechanism is not operative.

The two-photon absorption (2PA) spectra of **aBDP-1**, **aBDP-2**, **aBBDP-1**, **aBBDP-2**, **aNBDP-1**, and **aNBDP-2** are shown in Figure 5. Similar spectra for other BODIPY compounds can be found in the Supporting Information (Figure S16). Our experimental results show that in the lowest energy transition (S_0_ → S_1_), the 2PA cross sections are not large—10–20 GM for **aBDP**s, **aBBDP**s, and **aNBDP-1**, increasing up to ∼40 GM for **aNBDP-2**. In all cases, a vibronic transition peak, shifted to a shorter wavelength with respect to the pure electronic transition peak (corresponding to double the 1PA peak wavelength, black solid line in Figure 5), exhibits higher 2PA than the pure electronic one. We attribute this effect to a specific Herzberg–Teller-type coupling of the permanent dipole moment change to the bond-length alternating vibration, similar to what has been observed in fluorescent proteins.^68^

**Figure 5:**
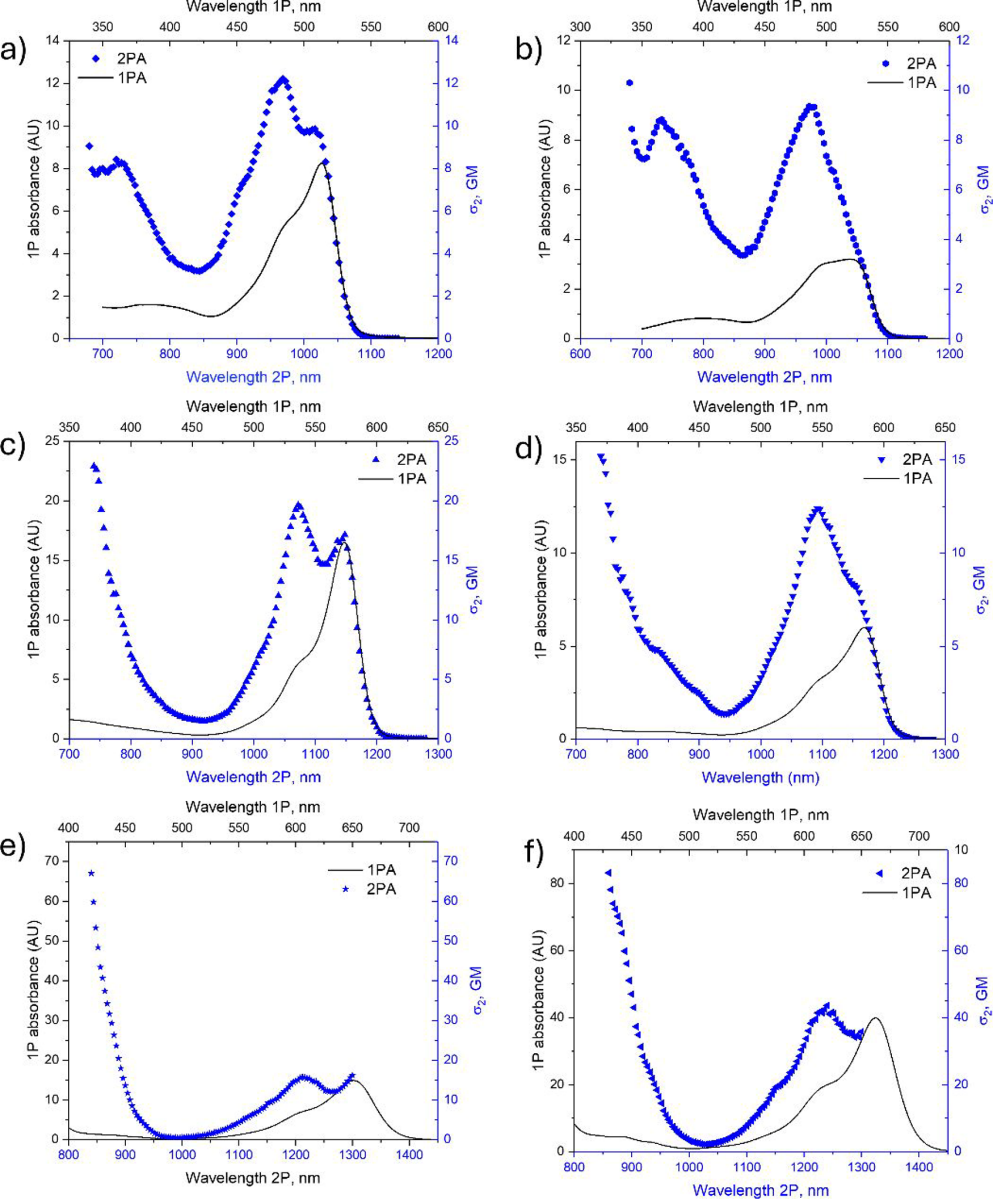
2PA spectra (data points) of **aBDP-1** (a), **aBDP-2** (b), **aBBDP-1** (c), **aBBDP-2** (d), **aNBDP-1** (e), and **aNBDP-2** (f) measured using deoxygenated DMSO as a solvent. The corresponding 1PA spectra (solid lines) are arbitrarily scaled. The red edge (1300 nm) was determined by the tunability range of the laser.^69^

For the studied compounds, the 2PA maxima corresponding to the higher energy S_0_ → S_n_ transitions could not be measured due to interference of linear (one-photon) absorption to the lower vibronic states of the S_0_ → S_1_ transition. For the **aBBDP** series in particular, linear absorption started to effectively compete with the 2PA, resulting in nonquadratic power dependencies of the fluorescence signals at wavelengths shorter than λ_2P_ < 740 nm. The same effect limited the measurements in the wavelength ranges shorter than 840 nm for **aNBDP-1** and shorter than 860 nm for **aNBDP-2**.

The 2PA spectra of **aBDP 1–6** exhibited a progressively intensifying band from 700 to 1100 nm (Figure 5 and S14). The maximum value of σ^2^ = 12.2 GM was observed for **aBDP**-1 at 968 nm. For the benzoBODIPY **aBBDP**-1, the 2PA increased nearly two-fold (σ^2^ = 22.9 GM) and emerged around 1070 nm (Figure 5c). Further increases in the 2PA cross-section were observed for **aNBDP**-1, reaching the maximal values for **aNBDP**-2 (σ^2^ = 83.2 GM). For these naphthoBODIPY compounds, 2PA extends to 1300 nm.

To gain insight into the effects of π-extension on the 2PA properties of BODIPY and to interpret the experimental 2PA measurements, we carried out electronic structure calculations for several model π-extended BODIPY dyes, including simulation of their linear as well as 2PA spectra using density functional theory (DFT), time-dependent DFT (TD-DFT), and the sum-overstates (SOS) formalism, as described previously.^70–72^ The computed spectra are shown in Figure 6, along with the structures of the studied molecules. Consistent with the experimental results (Table 2, Figure 5), the computed 2PA cross sections for the lowest excited states (S_1_) are not high for either of the chromophores, although there is a slight increase in 2PA with an increase in the size of the π-system. According to TD-DFT calculations (Figures S17–S19), in all cases, S_1_ transitions are essentially HOMO–LUMO single-electron excitations, whereby the HOMOs progressively extend onto the appended benzo- and then naphtho-fragments, which results in their destabilization and ensuing narrowing on the HOMO–LUMO gap.

**Figure 6:**
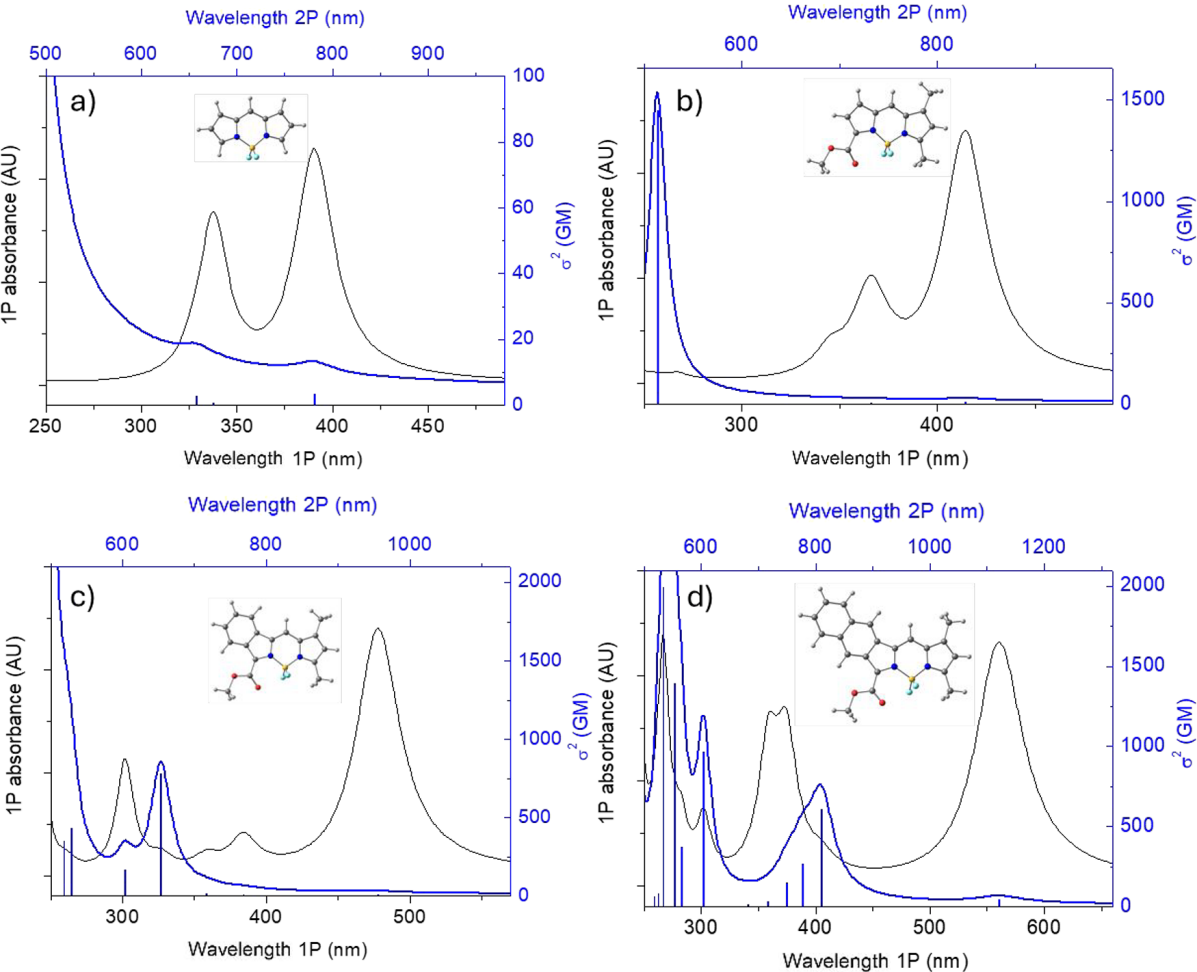
1PA (black lines) and 2PA spectra (blue bars) of parent BODIPY (a), 1-ethoxycarbonyl-3,7-dimethylBODIPY (b), **aBBDP-1** (c), and **aNBDP-1** (d) computed using TD-DFT/SOS methodology. Spectra for other BODIPY compounds can be found in the Supporting Information (Figures S19).

The lowest 2P-active transitions in all of the chromophores comprise configuration interactions involving additional excitations. The topologies of the orbitals involved (Figures 7, S17, and S18) do not inform the 2PA strengths of the corresponding transitions in any obvious way. However, an important trend that could be seen from the computed spectra is a decrease in the energy of the lowest 2P-active transition, with an increase in the size of the π-system. This latter tendency is consistent with an increase in the amplitude of the blue shoulder in the experimental 2PA spectra (Figure 5), which could be an indication of a strongly 2P-active band undergoing stabilization upon π-extension. In fact, our calculations predict that in BODIPY extended by fusion with both benzo- and naphtho-fragments, the corresponding 2P band should become significantly stabilized, resulting in a large increase in 2PA.

**Figure 7:**
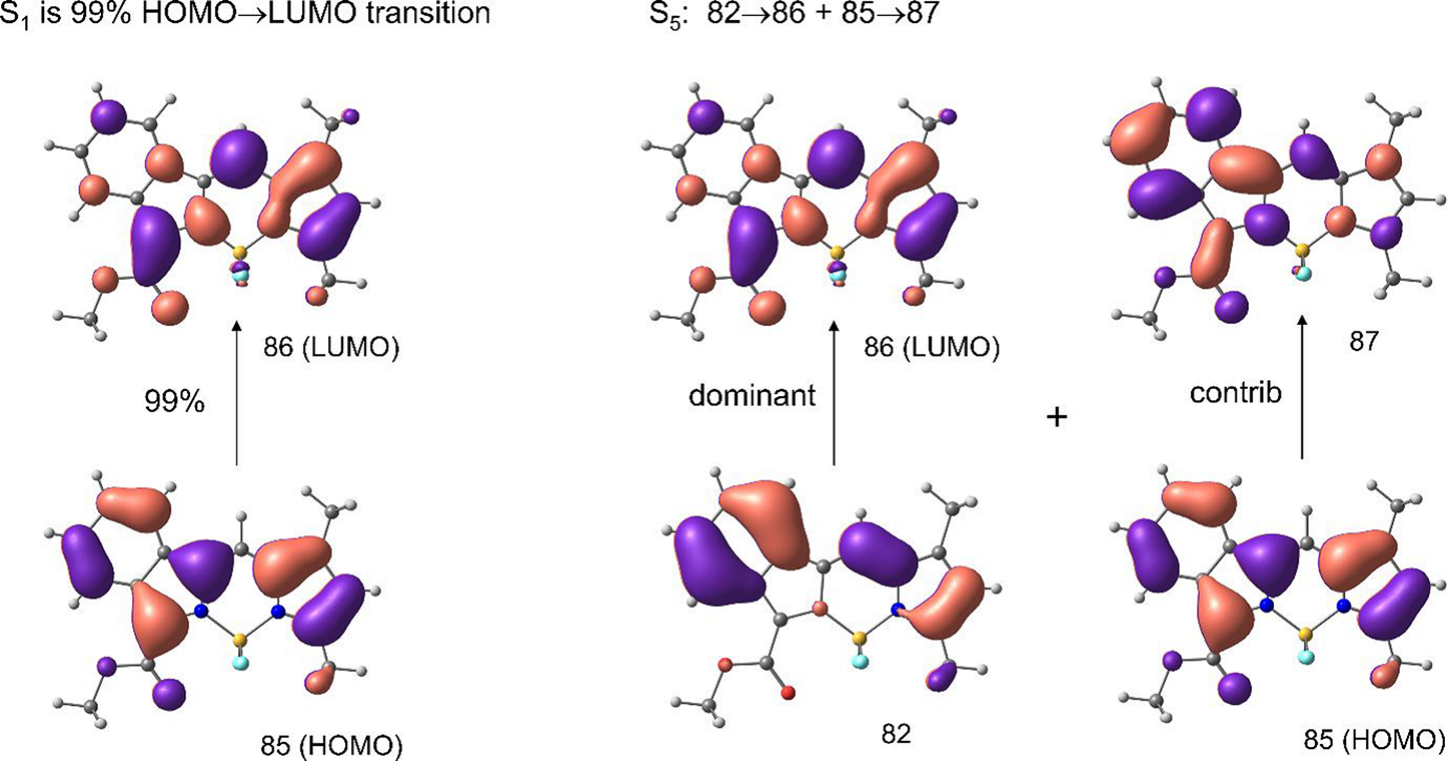
Orbitals involved in the 1P (S_1_) and 2P (S_5_) transitions of aBBDP-1.

The 2PA cross-section values for **aBBDP**-1 and **aNBDP**-1, while lower than those of the most efficient compounds in the series, remain relatively high compared to those of other symmetric BODIPY compounds, as predicted by the calculations. These results highlight the significant impact of unsymmetrical substitution and π-extension in enhancing the two-photon absorption properties of BODIPYs for advanced optical applications.

## Conclusions

In this study, we designed and synthesized a series of unsymmetric BODIPY derivatives (**aBBDP** and **aNBDP**) featuring π-extended systems through the incorporation of benzo- and naphtho-annulated pyrrolic units. The synthetic approach employed stable dihydro- and tetrahydroisoindole derivatives, enabling the formation of target BODIPY scaffolds via a modular condensation strategy followed by oxidative aromatization. Among the two explored synthetic routes, route B, employing precursors with the formyl group introduced in the alkoxycarbonyl pyrrole, proved more efficient for obtaining the target unsymmetric structures, with yields up to 85%.

Photophysical characterization revealed that π-extension and unsymmetrical substitution induce significant red-shifts in absorption and emission, with maxima spanning 590–680 nm and fluorescence quantum yields ranging from 0.27 to 0.84. Notably, the monobenzo derivative **aBBDP-1** exhibited the highest quantum yield (up to 0.86). The compounds also displayed solvent-dependent behavior, with variations in quantum yields and excited-state lifetimes linked to intramolecular charge-transfer effects. While precursor **aBDP** compounds generated singlet oxygen with moderate efficiency (up to 18%), no singlet oxygen generation was observed for the π-extended BODIPYs. Intramolecular charge-transfer interactions in these compounds are enhanced by introducing more electron-rich pyrrolic units, based on trialkyl-substituted pyrrolic derivatives. This lowers the excitation energy by stabilizing the excited state; however, at the same time, it leads to reduced oscillator strength and diminished fluorescence efficiency. Therefore, a careful balance between red-shift and radiative performance must be considered when incorporating CT character into dye design.

Two-photon absorption (2PA) studies demonstrated that π-extension leads to a pronounced increase in 2PA cross sections, reaching up to 83 GM for **aNBDP-2**. This enhancement was attributed to the stabilization of low-energy 2P-active transitions, according to TD-DFT/SOS calculations. The computational analysis confirmed that increasing the conjugated system size lowers the energy of the 2P-active states, while the experiments revealed that for the lowest energy state, the vibronic transition is characterized by a higher 2PA strength. These findings highlight the potential of unsymmetric, π-extended BODIPYs as promising fluorophores with tunable NIR properties and improved nonlinear optical performance for advanced imaging and photonic applications.

## Supplementary Material



## Data Availability

The data underlying this study are available in the published article and its Supporting Information.

## Notes

Synthetic procedures, NMR and optical spectroscopy data, singlet oxygen generation measurements data, quantum-chemical computations, and X-ray crystallography details (PDF)
