# Supplementary material for: Synthesis and Optical Properties of Unsymmetric Aromatically π‑Extended BODIPY
Source: J Org Chem. 2025 Sep 9;90(37):12984–97. doi: 10.1021/acs.joc.5c01432 (PMC12455648; doi:10.1021/acs.joc.5c01432)
Supplement: Supplementary file 1 [file jo5c01432_si_001.pdf]

Supporting Information

Synthesis and Optical Properties of Unsymmetric Aromatically π-Extended BODIPY

Metodej Dvoracek,[a] Craig Newman,[a] Mikhail Drobizhev,[b] Brendan Twamley,[c] Mathias O. Senge,[d] Sergei A. Vinogradov[e] and Mikhail A. Filatov[a*]

[a] School of Chemical and Biopharmaceutical Sciences, Technological University Dublin, City Campus, Grangegorman, Dublin 7, Ireland. Email: mikhail.filatov@tudublin.ie

[b] Department of Microbiology and Cell Biology, Montana State University, Bozeman, Montana 59717, United States

[c] School of Chemistry, Trinity College Dublin, The University of Dublin, Dublin 2, Ireland

[d] Medicinal Chemistry, Trinity Translational Medicine Institute, St. James's Hospital, Trinity College Dublin, The University of Dublin, Dublin D08 W9RT, Ireland

[e] Department of Biochemistry and Biophysics, Perelman School of Medicine, University of Pennsylvania, Philadelphia, USA; Department of Chemistry, School of Arts and Sciences, University of Pennsylvania, Philadelphia, USA.

Contents

1. General Procedures S2

2. Synthetic Procedures and Characterization S3

3. NMR spectra S7

4. Optical Properties S19

5. Computational Studies S26

6. Crystallographic Data S34

7. References S39

Experimental Section

1. General Procedures

^{1}H and ^{13}C NMR spectra were recorded on a Bruker Avance III 500 MHz spectrometer. Chemical shifts are reported as ppm relative to TMS and referenced to residual solvent peaks of CDCl3 (δ 7.26 ppm). Multiplicity assignments are abbreviated as follows: s = singlet, d = doublet, t = triplet, q = quartet, m = multiplet.

UV-Vis absorption spectra were recorded in solutions using Shimadzu UV-1900i and PerkinElmer Lambda 900 UV/VIS/NIR spectrometers (1 cm path length quartz cell). Fluorescence emission spectra were measured using FluoroMax-4 spectrometer. Emission quantum yields of the compounds were measured relative to the fluorescence of Rhodamine 6G as a standard (Φ_F = 0.95 in ethanol).[1] Sample concentrations were chosen to obtain an absorbance of 0.03-0.07 at the excitation wavelength; at least three measurements were performed for each sample.

Excited state lifetimes were determined by time correlated single photon counting (TCSPC) measurements using Horiba Scientific DeltaHub DH-HT High throughput TCSPC module equipped with a DeltaDiode-510L pulsed laser diode with peak wavelength 510nm +/-10nm. Datastation v2.4 software was used for data collection and analysis was conducted on Eztime software. Decay curves were fitted with mono- and bi-exponential functions and the quality was determined by the $\chi 2$ values.

Mass spectrometry analysis (HRMS) was performed with a Q-Tof Premier Waters MALDI quadrupole time-of-flight (Q-TOF) mass spectrometer equipped with Z-spray electrospray ionization (ESI) and matrix assisted laser desorption ionization (MALDI) sources in positive mode with trans-2-[3-(4-tertbutylphenyl)-2-methyl-2-propenylidene]malononitrile as the matrix. ESI mass spectra were acquired in positive modes as required, using a Micromass TOF mass spectrometer interfaced to a Waters 2960 HPLC or a Bruker microTOF-Q III spectrometer interfaced to a Dionex UltiMate 3000 LC. Atmospheric pressure chemical ionization (APCI) experiments were performed on a Bruker microTOF-Q III spectrometer interfaced to a Dionex UltiMate 3000 LC.

2. Synthetic Procedures and Characterization

The handling of all air/water sensitive materials was carried out using standard high vacuum techniques. Tetrahydrofuran and dichloromethane were distilled from $LiAlH_4$ and CaH_2, respectively. All other solvents were used as commercially supplied. Analytical thin layer chromatography was performed using silica gel 60 (fluorescence indicator F254, pre-coated sheets, 0.2 mm thick, 20 cm × 20 cm; Merck) plates and visualized by UV irradiation (λ = 254 nm). Column chromatography was carried out using silica gel 60 (0.063-0.2 mm).

2,3-Dichloro-5,6-dicyano-1,4-benzoquinone, N,N-dimethylformamide 1,8-diazabicyclo[5.4.0]undec-7-ene, Oxone® monopersulfate compound, ethyl isocyanoacetate, 2,4-dimethyl-1*H*-pyrrole, boron trifluoride diethyl etherate, N,N-diisopropylethylamine, ethyl 2,4-dimethyl-1H-pyrrole-3-carboxylate, phosphorus(V) oxychloride, potassium acetate and dimethylformamide were purchased from Sigma-Aldrich. Ethyl-4,5,6,7-tetrahydro-isoindole-1-carboxylate and ethyl-4,9-dihydro-2H-benzo[f]isoindole-1-carboxylate were prepared according to previously published procedures.[2,3]

General procedure for synthesis of pyrroles P6 and P7

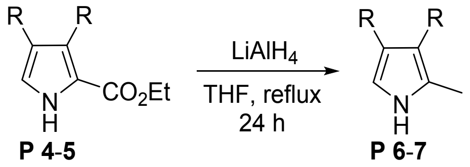

Compounds **P6 and P7** were prepared using a previously reported procedure.[4] $LiAlH_4$ (0.456 g, 12 mmol) was added to dry THF (30 mL) under nitrogen, the mixture was stirred and cooled to 0°C using an ice bath. Corresponding ethoxycarbonyl pyrrole **P4** or **P5** (3 mmol) was dissolved in dry THF (10 mL) and the solution was added dropwise into the reaction flask. The mixture was heated under reflux for 24 h using an oil bath, cooled to room and then to 0°C. Aqueous solution of NaOH (10%, 5 mL) was added slowly to quench the residual $LiAlH_4$. The resulting mixture was filtered through a short layer of silica (5 cm) which was then washed THF (20 mL). The solvent was removed in vacuum to give the target products which were used without additional purification.

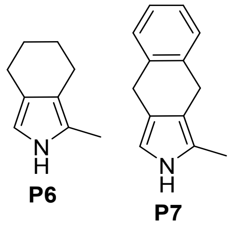

Compound **P6**. Yellow oil, 39% yield (158 mg). Analytical data are identical to previously described.[5]

Compound **P7**. Colorless amorphous solid, 46 % yield (253 mg). ^1H NMR (400 MHz, $CDCl_3$) δ 7.71 (s, 1H), 7.30 – 7.24 (m, 4H), 7.19 – 7.14 (m, 3H), 6.59 – 6.46 (m, 1H), 3.91 (s, 2H), 3.81 (s, 3H), 2.27 (s, 4H). $^{13}C\{^1H\}$ NMR (101 MHz, $CDCl_3$) δ 136.7, 136.6, 129.2, 129.1, 125.8, 125.7, 121.6, 118.9, 114.6, 110.2, 28.0, 27.1, 11.3.

General procedure for synthesis of formylpyrroles FP2-FP5

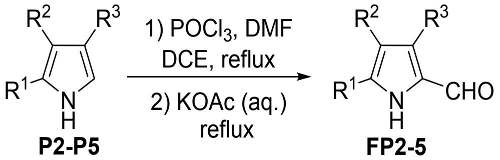

Compounds **FP2-FP4** were prepared using a modified literature procedure.[6] Phosphorus(V) oxychloride (1.687 g, 11 mmol) was added to DMF (0.804 g, 11 mmol) at 10 °C under N_2 and the resulting mixture was left to stir for 15 mins. The mixture was then diluted with 5-10 mL of 1,2-dichloroethane (DCE). A solution of pyrrole/pyrrole ester compound (10 mmol) in DCE (15-30 mL) was then added dropwise over 1 hour at 10°C. The reaction mixture was left to stir at room temperature for an additional hour and then heated at reflux using an oil bath under N_2 for 15 mins. The resulting mixture was cooled to room temperature and potassium acetate (5.398 g, 55 mmol) in H_2O (20 mL) was added. Reflux under N_2 was then continued for 15 mins. The crude product was extracted with DCM (2 × 50 mL), combined extracts were dried over Na_2SO_4 and evaporated in vacuum. The crude products were purified by recrystallization from MeOH-H_2O or EtOH-H_2O mixtures (varying ratio).

Compound **FP2**. 55% yield (677 mg). The analytical data are in agreement with those reported in the literature.[7]

Compound **FP3**. 50% yield (756 mg). The analytical data are in agreement with those reported in the literature.[6]

Compound **FP4.** 79% yield (1.747 g). The analytical data are in agreement with those reported in the literature.[8]

Compound **FP5.** 87% yield (2.343 g). ^{1}H NMR (400 MHz, CDCl3) δ 9.71 (s, 1H), 9.44 (s, 1H), 4.34 (q, *J* = 7.1 Hz, 2H), 2.82 (d, *J* = 20.4 Hz, 4H), 1.87 – 1.70 (m, 4H), 1.37 (t, *J* = 7.1 Hz, 3H). ^{13}C{^{1}H} NMR (101 MHz, $CDCl_3$) δ 178.9, 160.8, 134.2, 132.9, 129.7, 129.5, 129.2, 128.5, 126.9, 126.7, 126.6, 123.2, 61.3, 27.6, 26.1, 14.6.

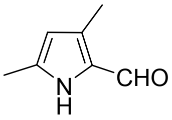

FP2

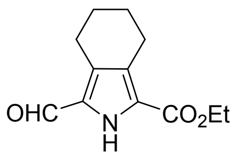

FP4

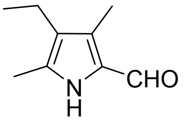

FP3

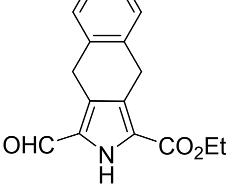

FP5

General procedure for the synthesis of compounds aBDP 1-6

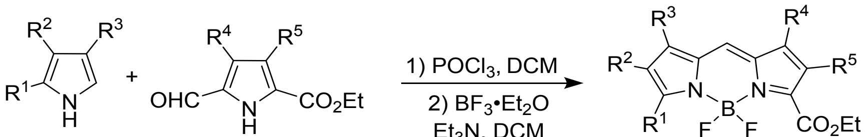

P 2-4
P 6-7
FP 4-5
aBDP 1-6

Compounds **aBDP 1-6** were prepared using a modified literature procedure.[9] A mixture of formylpyrrole (**FP2-FP5**, 1 mmol) and corresponding pyrrole (0.123 g, 1 mmol) in 20 mL of dichloromethane (DCM) was cooled with an ice bath under flow of N_2. Phosphorus(V) oxychloride (0.184 g, 1.2 mmol) was then added in one portion. The reaction mixture was left to stir for 16 hr at room temperature. After stirring, the reaction was cooled with an ice bath and Et_3N (0.506 g, 5 mmol), followed by $BF_3{\cdot}Et_2O$ (0.852 g, 6 mmol) was then added dropwise, and the resulting mixture was left to stir for an additional 16 hr at room temperature. The mixture was then diluted with DCM (50 mL), washed with H_2O (2 × 50 mL), dried over Na2SO4 and evaporated in vacuum. The crude product was purified by column chromatography on a hexane-packed silica column using hexane-EtOAc mixture (2:1 v/v) as an eluent. The symmetrical by-products, **sBDP-1** and **sBDP-2** were isolated as an early eluting fraction during chromatographic purification.

Compound **sBDP-1**. The analytical data are in agreement with the literature.[10] ^{1}H NMR (400 MHz, $CDCl_3$) δ 7.04 (s, 1H), 6.04 (s, 2H), 2.52 (s, 6H), 2.24 (s, 6H). HRMS (APCI): *m/z* found 271.1174 calcd for (M^+) $C_{13}H_{15}BF_2N_2Na$ 271.1194.

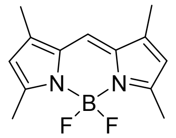
sBDP-1

Compound **sBDP2**. The analytical data are in agreement with the literature.[11] ^{1}H NMR (400 MHz, $CDCl_3$) δ 6.97 (d, *J* = 20.8 Hz, 1H), 2.49 (s, 6H), 2.38 (q, *J* = 7.6 Hz, 4H), 2.17 (d, *J* = 3.3 Hz, 6H), 1.06 (t, *J* = 7.6 Hz, 6H). HRMS (APCI): *m/z* found 327.1831, calcd for (M^+) $C_{17}H_{22}BF_2N_2Na$ 327.1820.

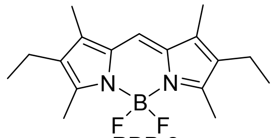
sBDP-2

Compound **aBDP-1.** 45% yield (156 mg). The analytical data are in agreement with those reported in the literature.[8]

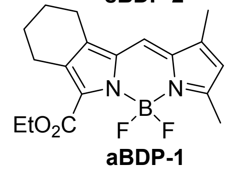

aBDP-1

Compound **aBDP-2.** 75% yield (281 mg). The analytical data are in agreement with those reported in the literature.[8]

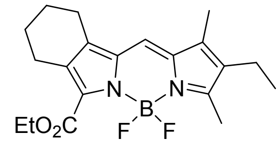

aBDP-2

Compound **aBDP-3.** 41% yield (162 mg). ^{1}H NMR (400 MHz, CDCl3) δ 7.30 – 7.21 (m, *J* = 12.6, 7.4, 2.7 Hz, 4H) overlapping with solvent peak, 7.19 (s, 1H), 6.15 (s, 1H), 4.44 (q, *J* = 7.1 Hz, 2H), 4.02 (d, *J* = 3.3 Hz, 4H), 2.62 (s, 3H), 2.27 (s, 3H), 1.45 (t, *J* = 7.1 Hz, 3H).^{13}C{^{1}H} NMR (101 MHz, CDCl3) δ 167.2, 161.2, 146.5, 138.0, 135.3, 134.7, 133.8, 132.5, 131.5, 129.7, 129.1, 126.6, 126.4, 123.2, 121.7, 61.1, 28.6, 26.4, 15.8, 14.4, 11.6. HRMS (APCI): *m/z* found 394.1674, calcd for (M^+) $C_{22}H_{21}BF_2N_2O_2$ 394.1664.

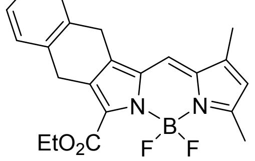

aBDP-3

Compound **aBDP-4.** 89% yield (376 mg). ^{1}H NMR (400 MHz, Acetone) δ 7.68 (s, 1H), 7.33 – 7.25 (m, 2H), 7.22 – 7.17 (m, *J* = 4.7 Hz, 2H), 4.36 (q, *J* = 7.1 Hz, 2H), 4.07 (d, *J* = 7.2 Hz, 5H), 2.59 (s, 3H), 2.48 (q, *J* = 7.6 Hz, 2H), 2.30 (s, 3H), 1.42 (t, *J* = 7.1 Hz, 3H), 1.10 (t, *J* = 7.6 Hz, 3H). ^{13}C{^{1}H} NMR (101 MHz, Acetone) δ 206.3, 168.0, 161.7, 142.9, 138.6, 137.6, 135.6, 134.7, 134.2, 133.9, 131.9,

130.4, 130.1, 129.9, 127.2, 127.1, 122.7, 61.1, 29.1, 26.9, 17.8, 14.7, 14.3, 13.9, 9.5·HRMS (APCI): *m/z* found 422.1980, calcd for (M^+) $C_{24}H_{25}BF_2N_2O_2$ 422.1977.

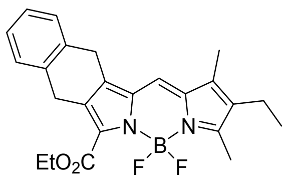

aBDP-4

Compound **aBDP-5.** 20% Yield (87 mg). ^{1}H NMR (400 MHz, CDCl3) δ 7.31 – 7.28 (m, 1H), 7.25 – 7.19 (m, 3H), 4.45 (q, *J* = 7.1 Hz, 2H), 4.05 (d, *J* = 34.7 Hz, 4H), 2.62 (s, 2H), 2.55 (s, 3H), 2.30 (s, 2H), 1.78 (s, 4H), 1.46 (t, *J* = 7.1 Hz, 3H). ^{13}C{^{1}H} NMR (101 MHz, CDCl3) δ 167.0, 161.3, 144.6, 136.8, 134.8, 133.7, 132.8, 132.1, 131.1, 129.7, 129.5, 129.1, 126.5, 126.3, 119.8, 60.9, 28.8, 26.3, 22.5, 22.0, 21.4, 20.9, 14.5, 13.8. HRMS (APCI): *m/z* found 434.1987, calcd for (M^+) $C_{25}H_{25}BF_2N_2O_2$ 434.1977.

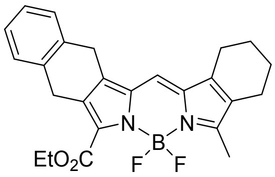

aBDP-5

Compound **aBDP-6.** 30% yield (130 mg). ^{1}H NMR (400 MHz, CDCl3) δ 7.26 (s, 3H), 7.09 (s, 1H), 4.39 (q, *J* = 7.1 Hz, 2H), 3.99 (s, 2H), 3.64 (s, 2H), 2.71 (s, 2H), 2.64 (s, 5H), 1.75 (s, 4H), 1.39 (t, *J* = 7.1 Hz, 4H). ^{13}C{^{1}H} NMR (101 MHz, CDCl3) δ 164.4, 161.4, 140.8, 136.5, 135.6, 135.4, 132.7, 132.0, 131.9, 131.8, 130.6, 129. 8, 129. 3, 127.0, 126.9, 120.8, 60.8, 29.8, 26.6, 22.8, 22.6, 21.6, 14.4, 13.9·HRMS (APCI): *m/z* found 434.1987, calcd for (M^+) $C_{25}H_{25}BF_2N_2O_2$ 434.1977.

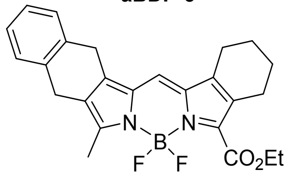

aBDP-6

General procedure for the synthesis of benzo- and napthoBODIPYs

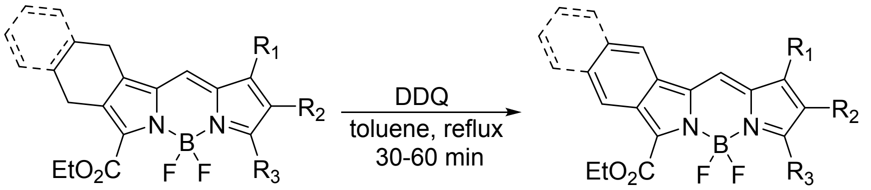

aBDP 1-4 → **aBBDP1-2, aNBDP1-2**

A modified literature procedure was used.[12] Selected **aBDP** compound (0.5 mmol) was mixed with DDQ (1.5 mmol for compounds **aBDP 1-2**; 0.75 mmol for compounds **aBDP 3-4**) in toluene (10-30 mL). The mixture was then heated at reflux using an oil bath under N_2 for 30-60 min. A color change from a dark red/purple colour to a vibrant magenta colour was observed upon formation of benzoBODIPY products, and from a dark black/green color to a vibrant emerald color was recorded for the naphthoBODIPY products. The reaction progress was monitored by TLC using DCM as an eluent. After completion, the mixture was diluted with DCM (50 mL) and washed with H2O (2 × 50 mL). The crude product was purified on a silica column using DCM as an eluent.

Compound **aBBDP-1**. 45% yield (77 mg). ^{1}H NMR (400 MHz, $CDCl_3$) δ 8.19 (d, *J* = 8.3 Hz, 1H), 7.78 (d, *J* = 8.1 Hz, 1H), 7.47 (s, 1H), 7.42 – 7.29 (m, 2H), 6.13 (s, 1H), 4.56 (q, *J* = 7.1 Hz, 2H), 2.63 (s, 3H), 2.30 (s, 3H), 1.53 (dd, *J* = 15.6, 8.5 Hz, 3H). ^{13}C{^{1}H} NMR (101 MHz, $CDCl_3$) δ 163.7, 160.6, 144.5, 136.5, 134.6, 133.7, 130.7, 128.8, 128.3, 126.5, 124.1, 121.8, 119.5, 118.5, 61.7, 15.6, 14.4, 11.6. HRMS (APCI): *m/z* found 342.1358, calcd for (M^+) $C_{18}H_{17}BF_2N_2O_2$ 342.1351.

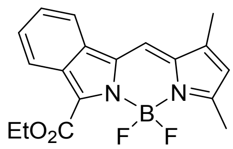

aBBDP-1

Compound **aBBDP-2**. 74% yield (137 mg). ^{1}H NMR (400 MHz, CDCl3) δ 8.20 (d, *J* = 8.2 Hz, 1H), 7.77 (d, *J* = 8.1 Hz, 1H), 7.40 (s, 1H), 7.34 (m, *J* = 21.2, 6.9 Hz, 2H), 4.55 (q, *J* = 7.1 Hz, 2H), 2.62 (s, 3H), 2.39 (q, *J* = 7.6 Hz, 2H), 2.22 (s, 3H), 1.51 (t, *J* = 7.1 Hz, 3H), 1.09 (t, *J* = 7.6 Hz, 3H). ^{13}C{^{1}H} NMR (101 MHz, CDCl3) δ 164.4, 160.7, 140.1, 136.5, 135.6, 133.1, 132.8, 130.7, 128.5, 127.7, 126.3, 124.0, 118.5, 118.3, 61.4, 29.8, 17.5, 14.5, 14.3, 13.7, 9.6. HRMS (APCI): *m/z* found 370.1678, calcd for (M^+) $C_{20}H_{21}BF_2N_2O_2$ 370.1664

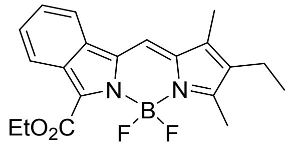

aBBDP-2

Compound **aNBDP-1**. 33% yield (65 mg). ^{1}H NMR (400 MHz, CDCl3) δ 8.63 (s, 1H), 8.28 (s, 1H), 7.91 (dd, *J* = 13.5, 8.5 Hz, 2H), 7.53 (s, 1H), 7.49 – 7.39 (m, 2H), 6.01 (s, 1H), 4.62 (q, *J* = 7.1 Hz, 2H), 2.59 (s, 3H), 2.29 (s, 3H), 1.57 (t, *J* = 6.1 Hz, 4H). ^{13}C{^{1}H} NMR (101 MHz, CDCl3) δ 160.45, 159.84, 142.18, 135.15, 133.77, 132.61, 131.40, 130.08, 129.32, 128.61, 127.24, 125.74, 124.19, 120.30, 117.64, 117.11, 62.07, 15.25, 14.42, 11.52.HRMS (APCI): *m/z* found 392.1501, calcd for (M^+) $C_{22}H_{19}BF_2N_2O_2$ 392.1508

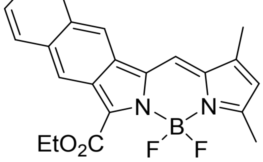

aNBDP-1

Compound **aNBDP-2**. 36% yield (76 mg). ^{1}H NMR (400 MHz, CDCl3) δ 8.67 (s, 1H), 8.27 (s, 1H), 7.90 (dd, *J* = 17.2, 8.4 Hz, 2H), 7.44 (s, 1H), 7.40 (m, *J* = 16.0, 7.6 Hz, 2H), 4.62 (q, *J* = 7.1 Hz, 2H), 2.52 (s, 3H), 2.21 (q, *J* = 7.6 Hz, 2H), 2.16 (s, 3H), 1.57 (t, *J* = 7.2 Hz, 3H), 1.02 (t, *J* = 7.6 Hz, 3H). ^{13}C{^{1}H} NMR (101 MHz, $CDCl_3$) δ 160.6, 138.5, 135.2, 134.5, 134.1, 133.4, 132.6, 131.1, 130.0, 129.5, 129.0, 128.6, 126.8, 125.5, 123.6, 117.1, 116.7, 61.8, 17.3, 14.5, 14.4, 13.3, 9.5. HRMS (APCI): *m/z* found 420.1826, calcd for (M^+) $C_{24}H_{23}BF_2N_2O_2$ 420.1821.

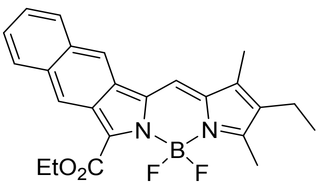

aNBDP-2

Synthesis of compound aNBDP3

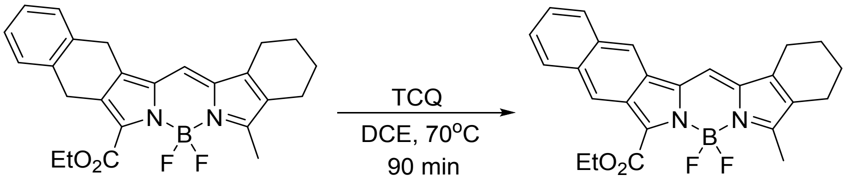

aBDP-5 **aNBDP-3**

Compound **aBDP-5** (0.0645 mmol) was mixed with tetrachlorobenzoquinone (0.0645 mmol) in 1,2-dichloroethane (10 mL). The mixture was then heated at 70 °C with an oil bath under N_2 for 90 min. The reaction progress was monitored by TLC using DCM as an eluent. After completion, the mixture was diluted with DCM (50 mL) and washed with H2O (2 × 50 mL). The crude product was purified on a silica column using DCM as an eluent.

Compound **aNBDP-3**. 45% yield (12.6 mg). ^{1}H NMR (400 MHz, $CDCl_3$) δ 8.74 (s, 1H), 8.29 (s, 1H), 7.90 (m, *J* = 13.5, 6.8 Hz, 2H), 7.51 – 7.35 (m, 3H), 4.63 (q, *J* = 7.1 Hz, 2H), 2.46 (s, 3H), 1.76 (s, 3H), 1.58 (t, *J* = 7.0 Hz, 7H) overlapping signals. ^{13}C{^{1}H} NMR (101 MHz, $CDCl_3$) δ 175.8, 160.7, 142.0, 134.3, 133.3, 132.5, 130.2, 129.9, 129.9, 128.7, 127.2, 126.7, 125.5, 123.5, 117.1, 116.4, 61.7, 22.7, 22.3, 21.5, 20.8, 14.5, 13.2. HRMS (APCI): *m/z* found 432.1827, calcd for (M^+) $C_{25}H_{23}BF_2N_2O_2$ 432.1821.

3. NMR spectra

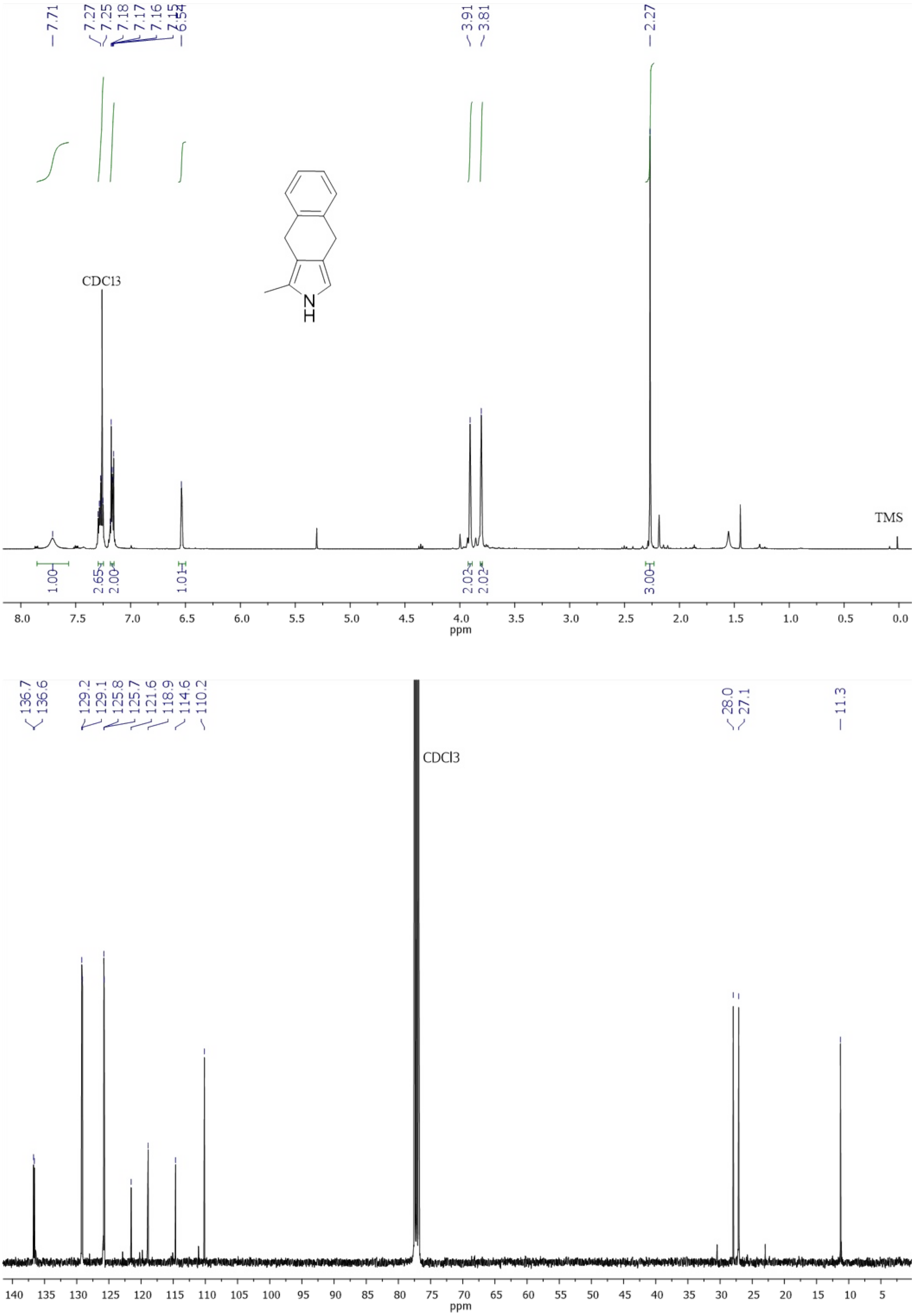

Figure S1: ^{1}H (400 MHz) and ^{13}C NMR (101 MHz) spectra of compound **P7** in $CDCl_3$**.**

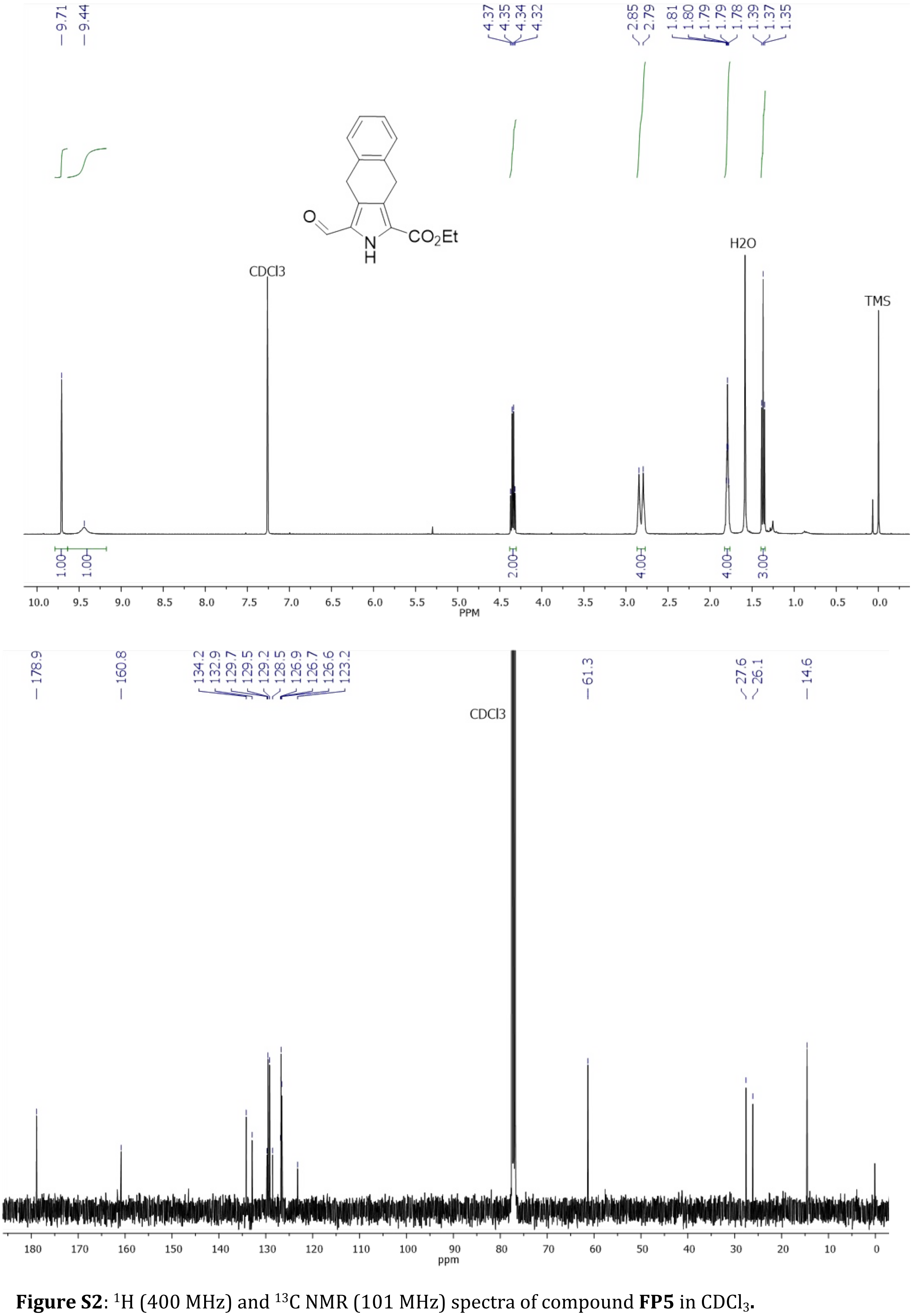

Figure S2: ^{1}H (400 MHz) and ^{13}C NMR (101 MHz) spectra of compound **FP5** in $CDCl_3$**.**

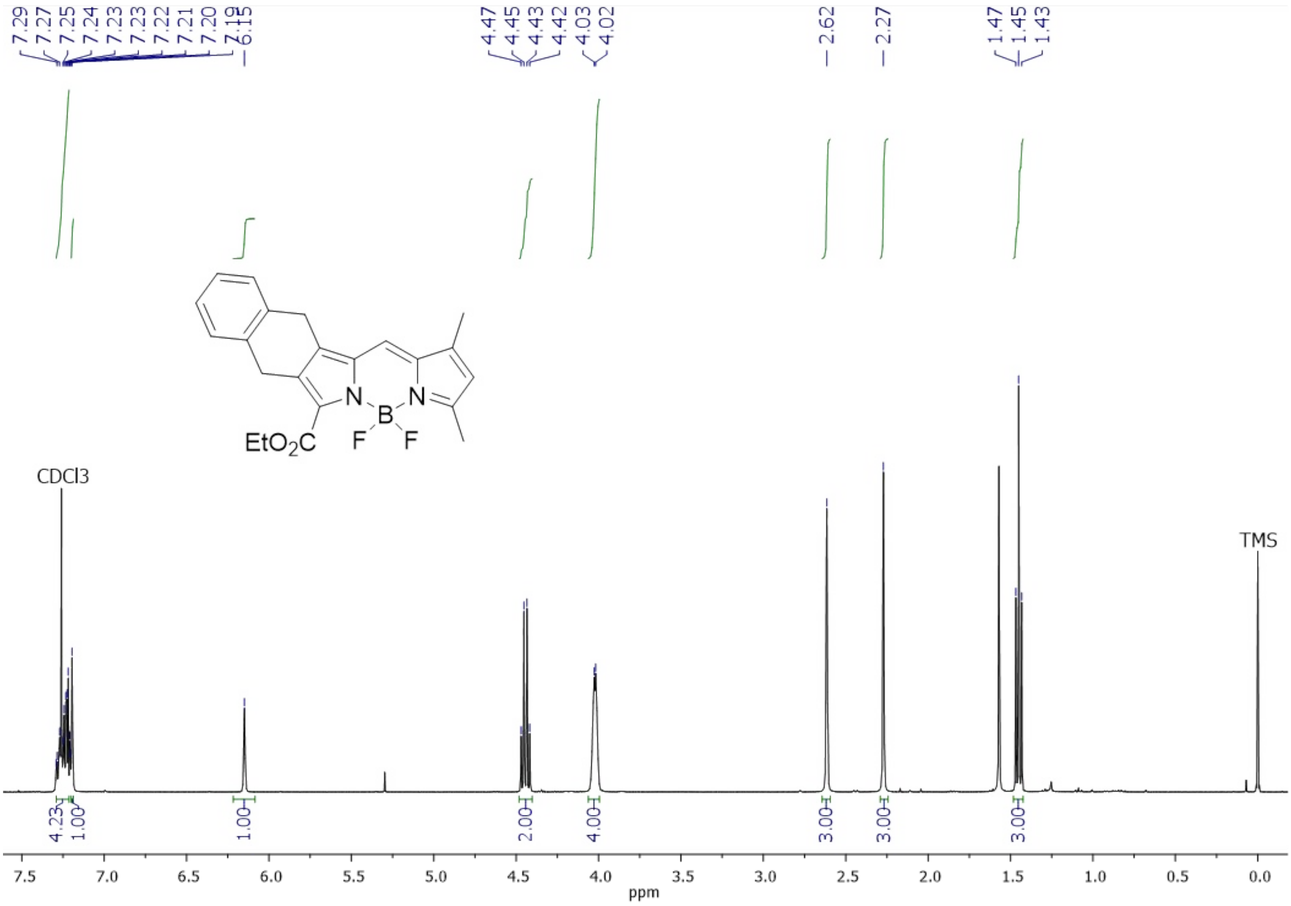

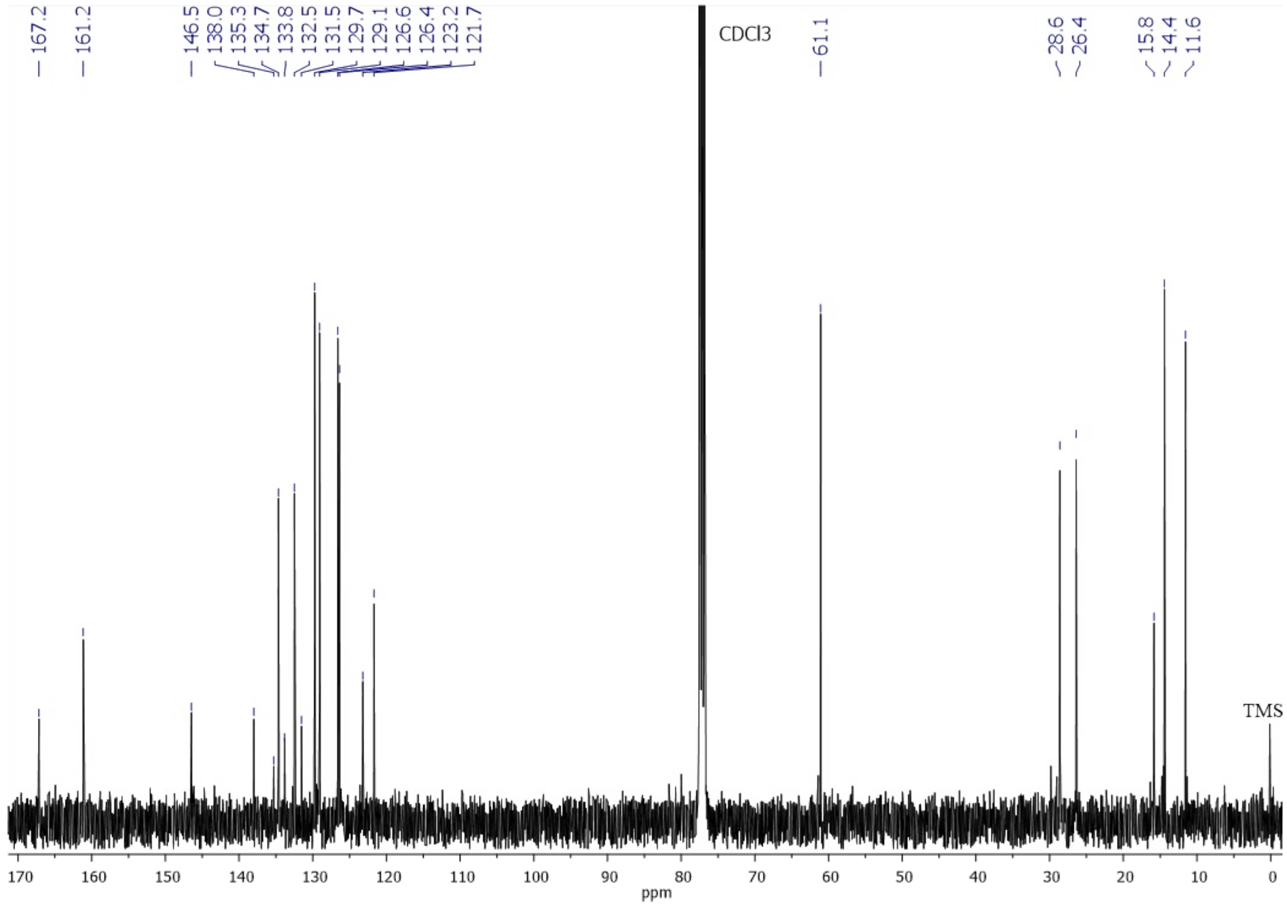

Figure S3: ^{1}H (400 MHz) and ^{13}C NMR (101 MHz) spectra of compound **aBDP-3** in $CDCl_3$**.**

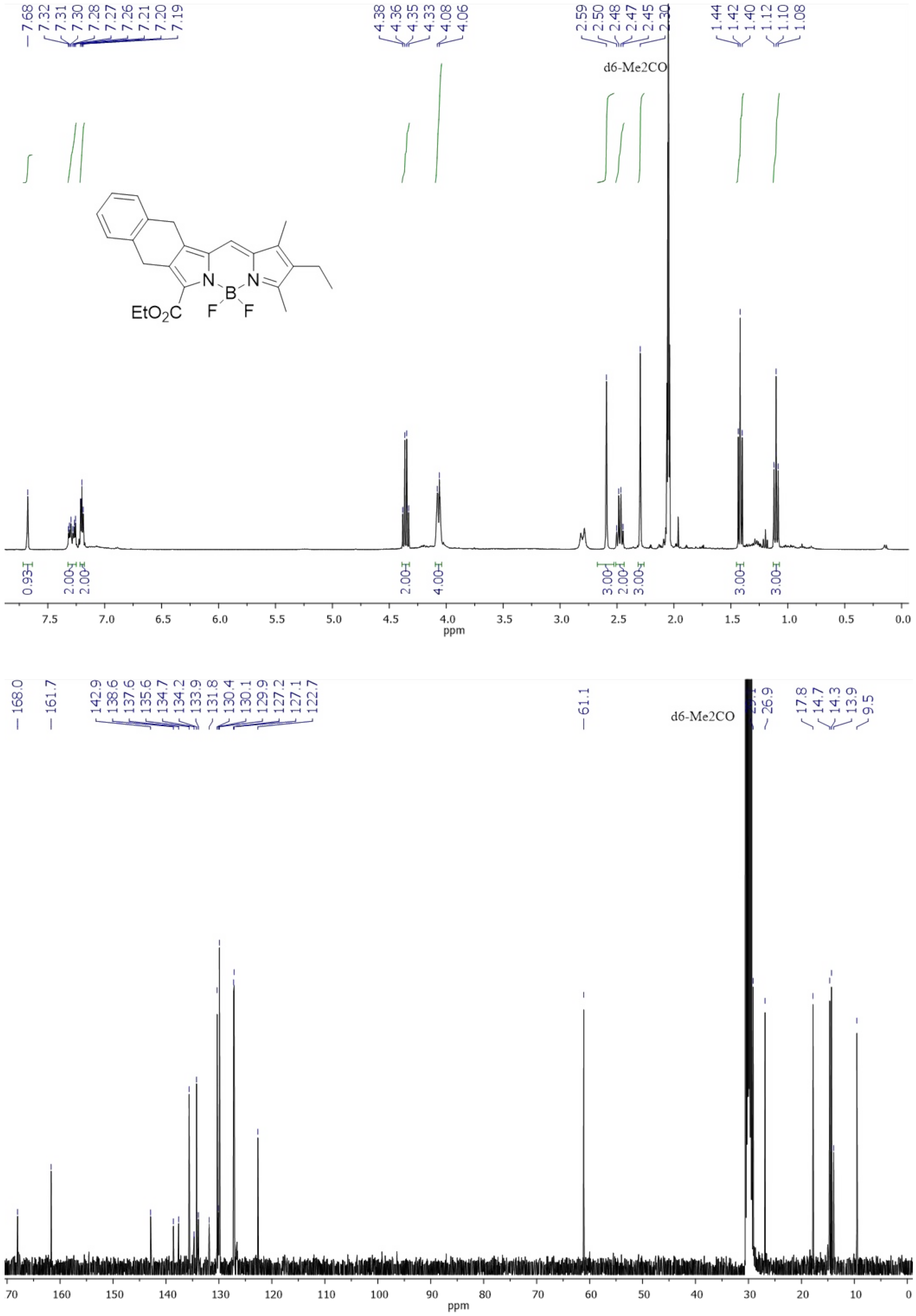

Figure S4: ^{1}H (400 MHz) and ^{13}C NMR (101 MHz) spectra of compound **aBDP-4** in d^6-acetone**.**

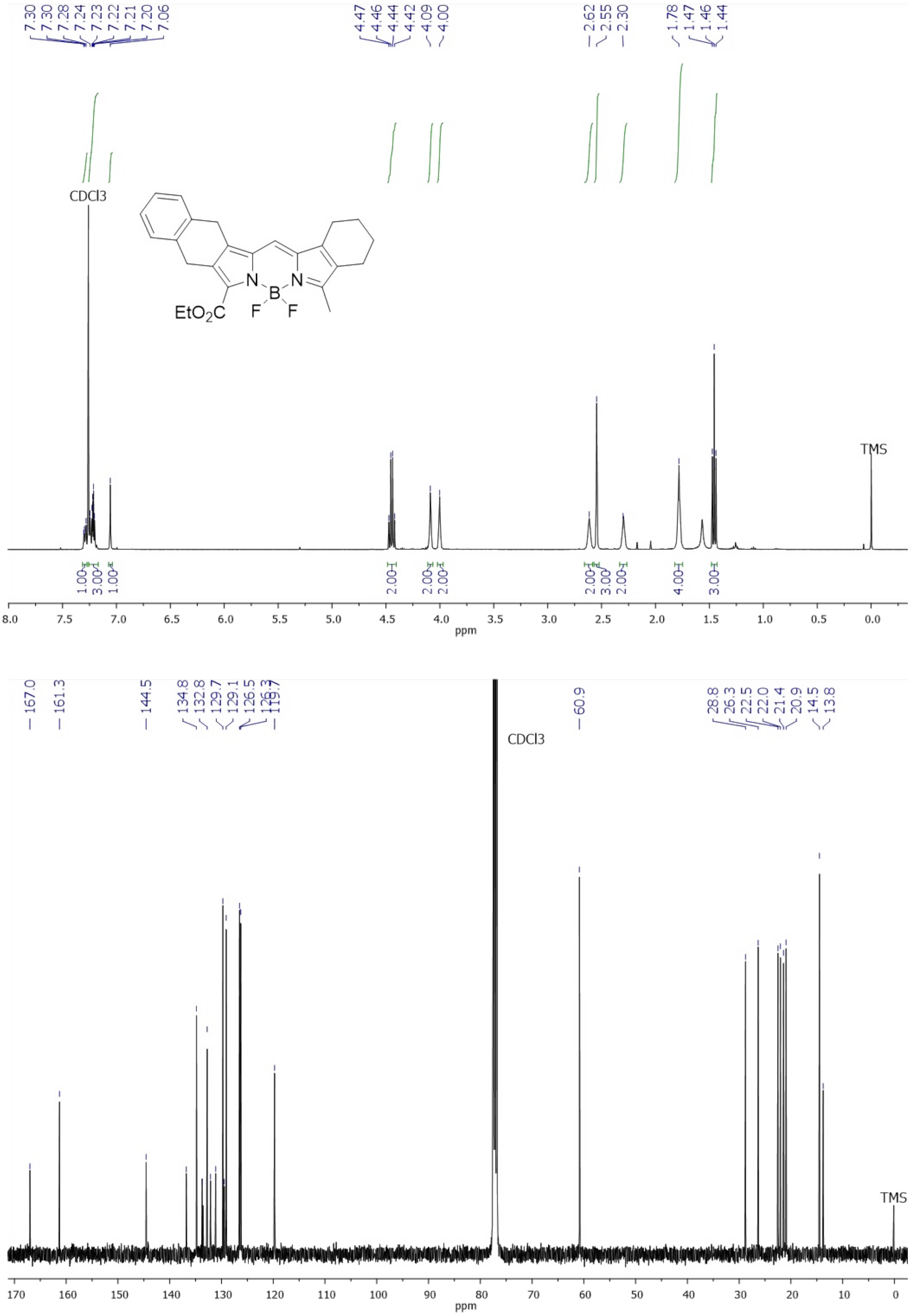

Figure S5: ^{1}H (400 MHz) and ^{13}C NMR (101 MHz) spectra of compound **aBDP-5** in $CDCl_3$.

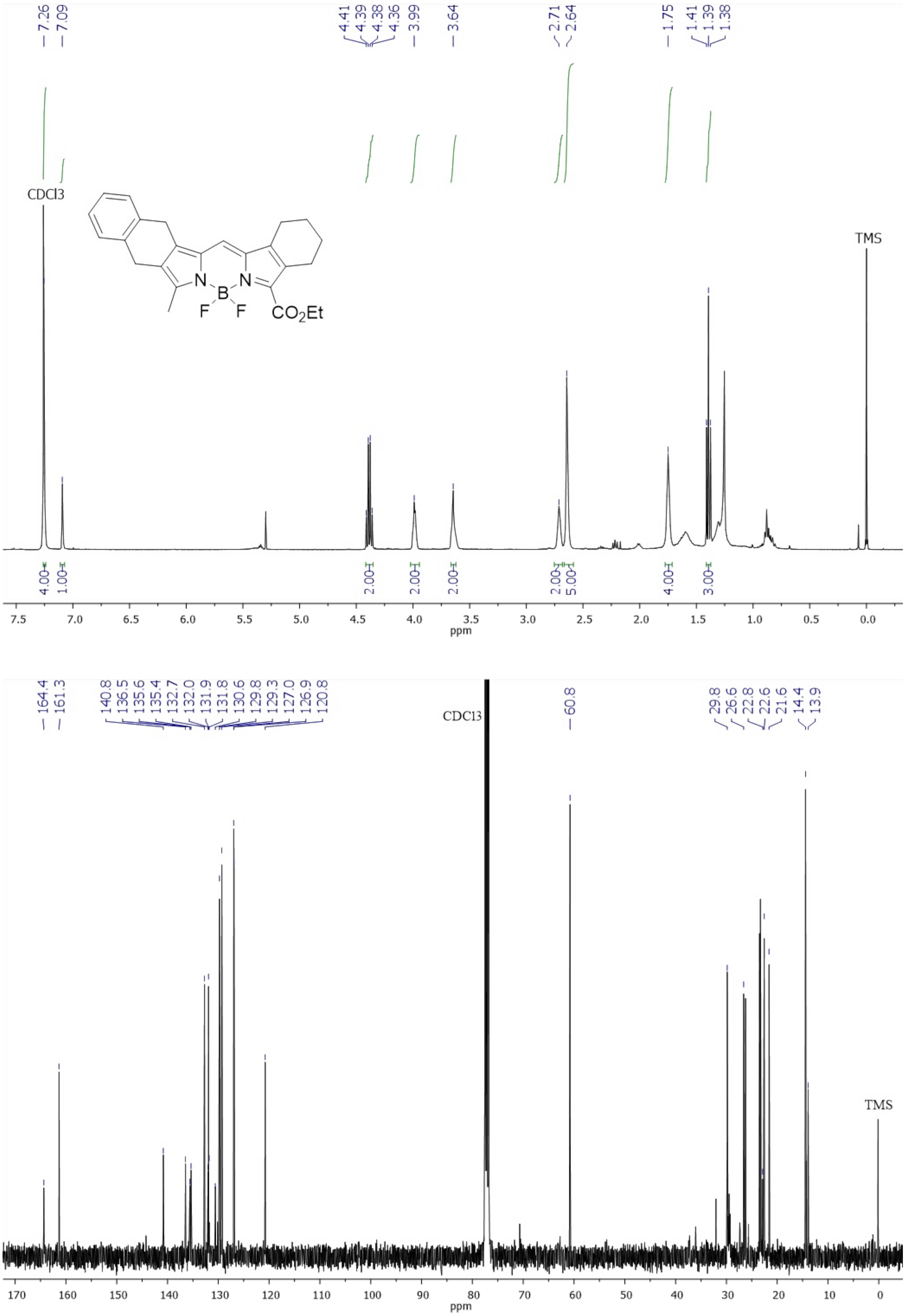

Figure S6: ^{1}H (400 MHz) and ^{13}C NMR (101 MHz) spectra of compound **aBDP-6** in $CDCl_3$.

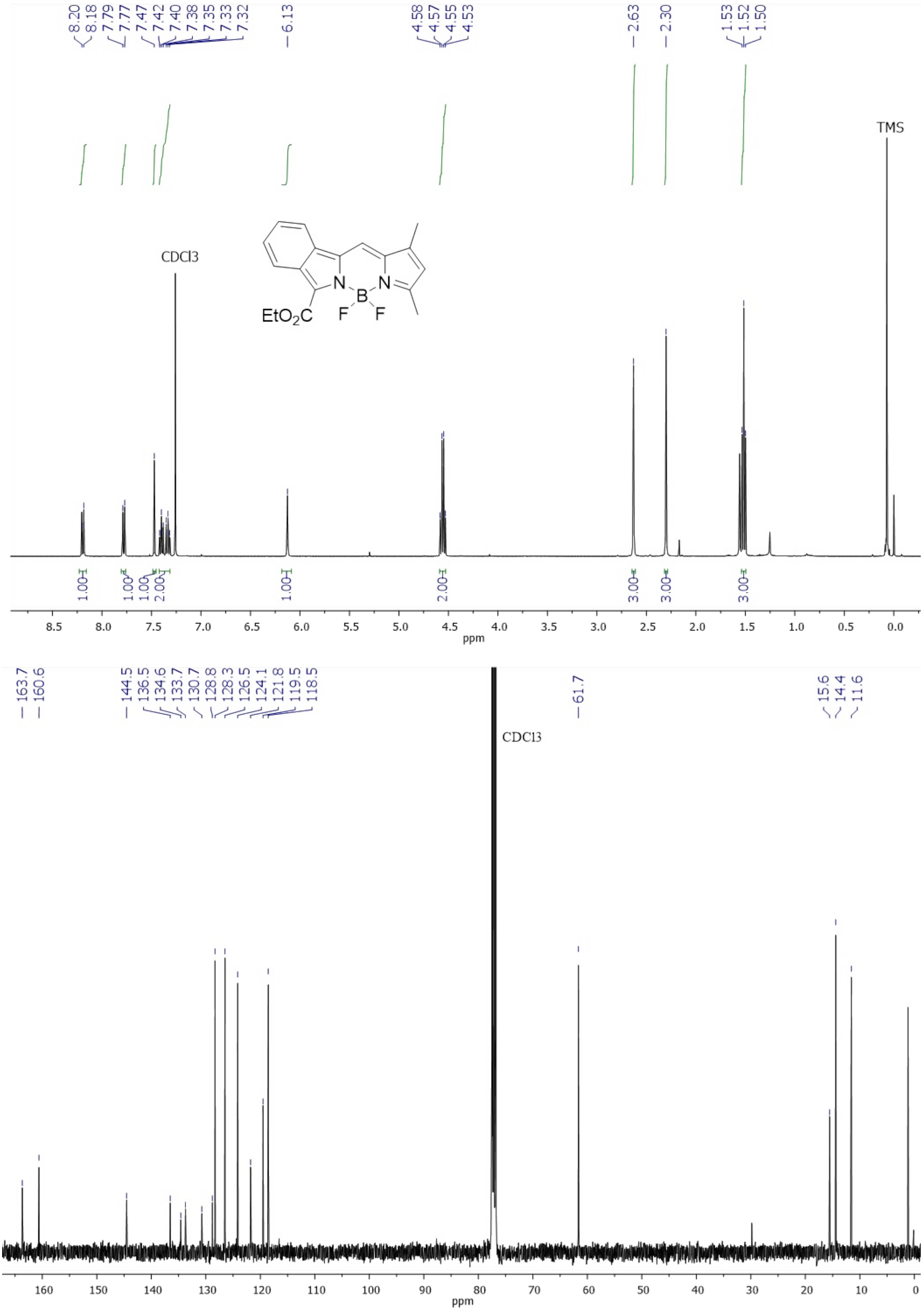

Figure S7: ^{1}H (400 MHz) and ^{13}C NMR (101 MHz) spectra of compound **aBBDP-1** in $CDCl_3$.

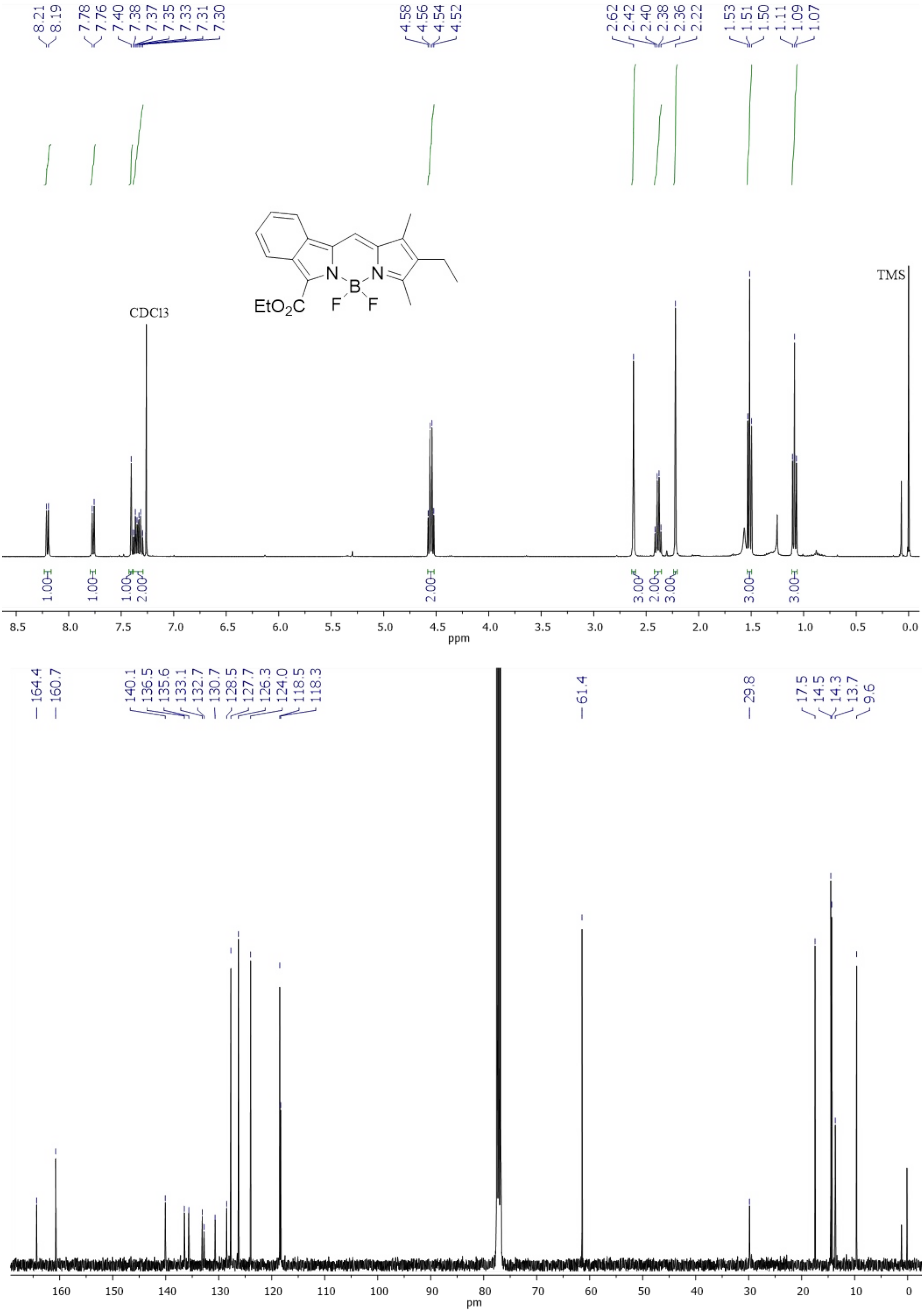

Figure S8: ^{1}H (400 MHz) and ^{13}C NMR (101 MHz) spectra of compound **aBBDP-2** in $CDCl_3$.

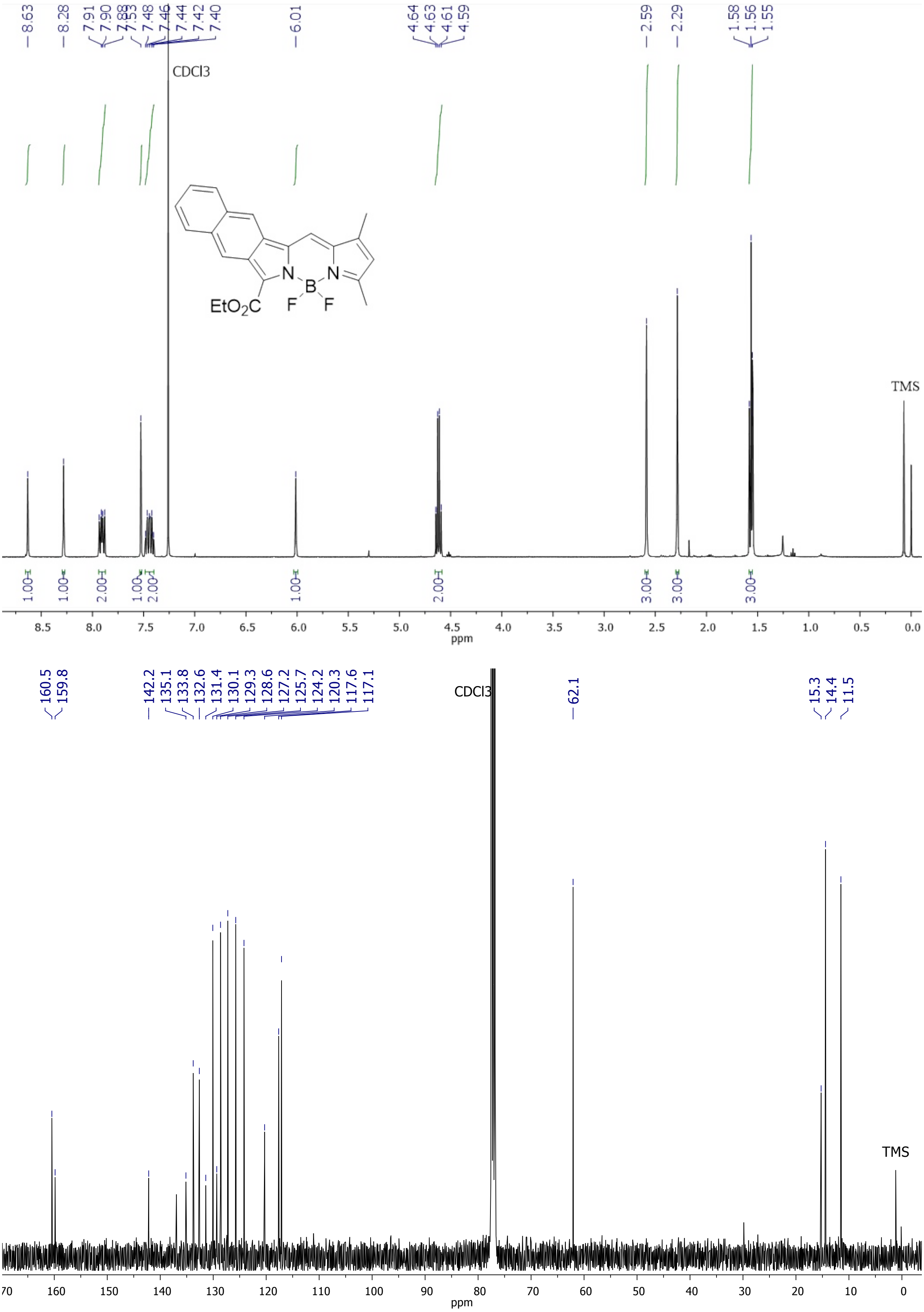

Figure S9: ^{1}H (400 MHz) and ^{13}C NMR (101 MHz) spectra of compound **aNBDP-1** in $CDCl_3$.

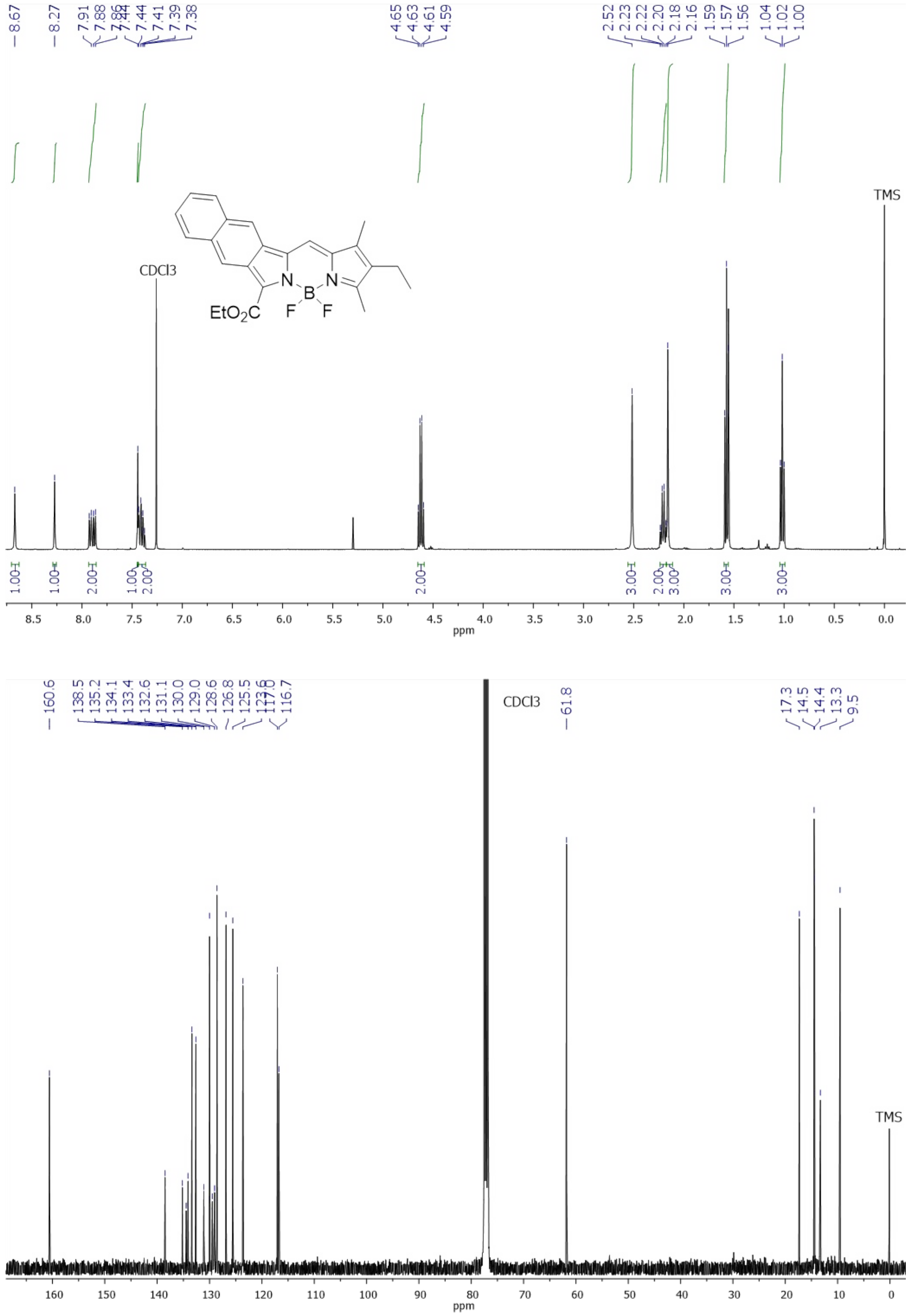

Figure S10: ^{1}H (400 MHz) and ^{13}C NMR (101 MHz) spectra of compound **aNBDP-2** in $CDCl_3$.

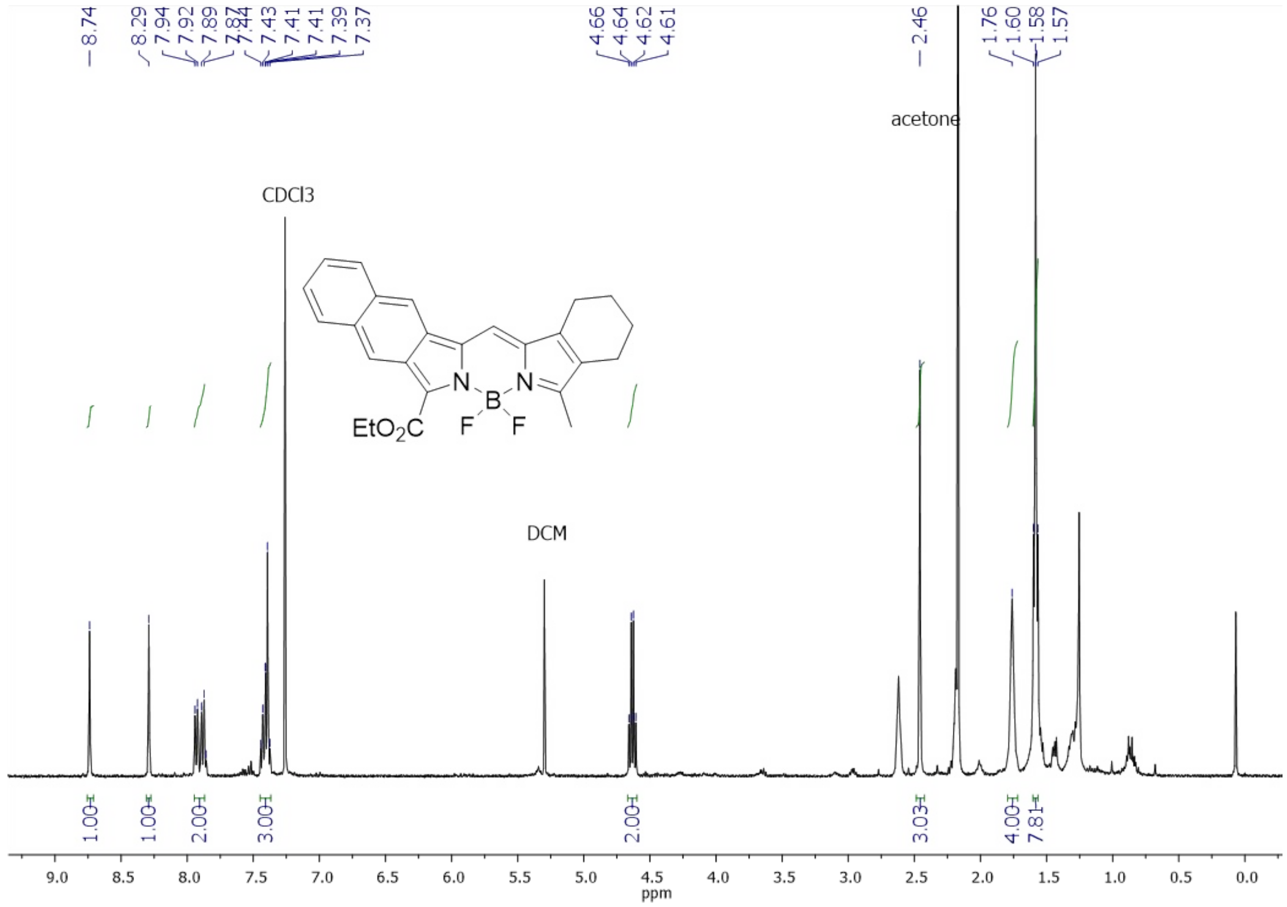

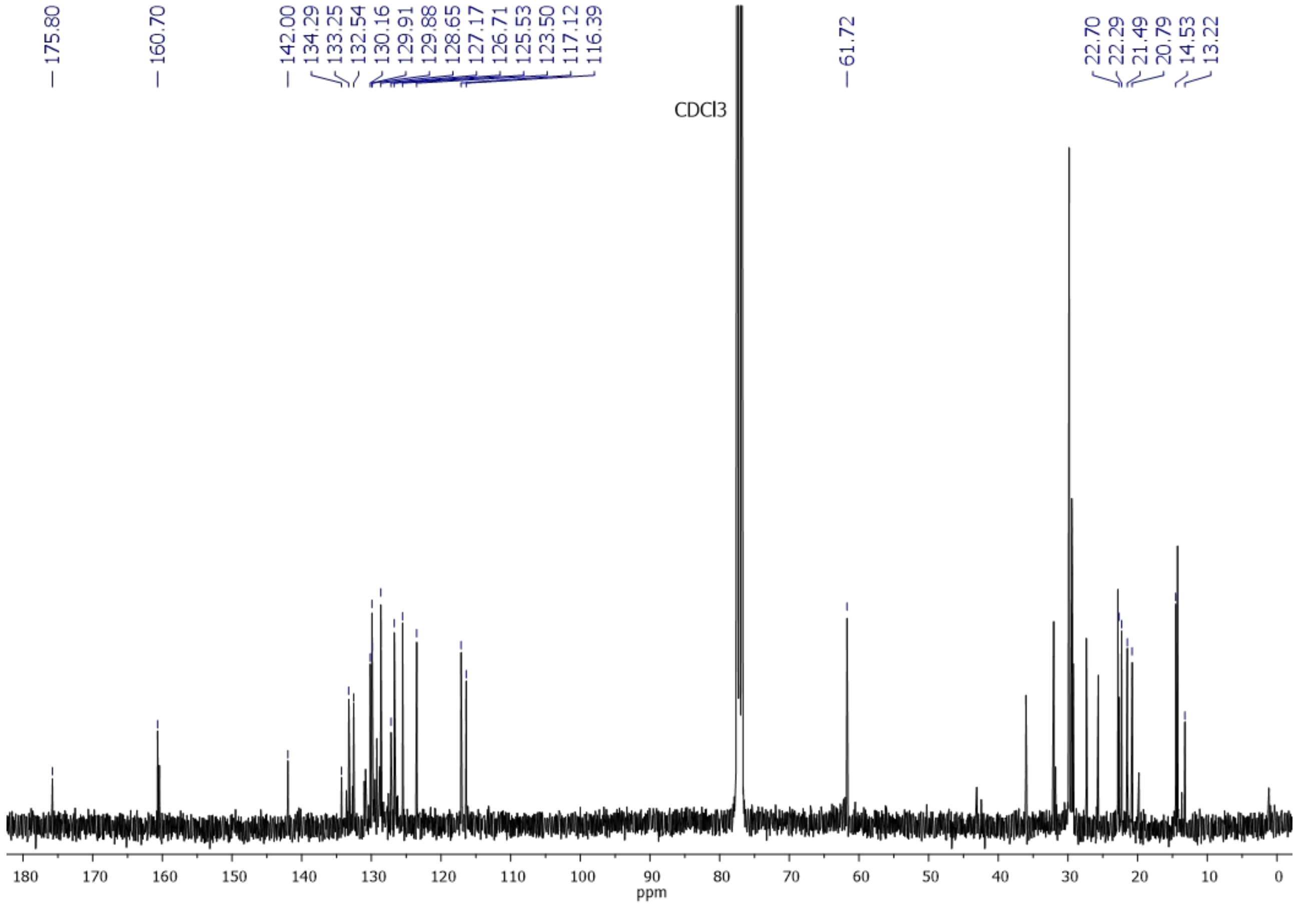

Figure S11: ^{1}H (400 MHz) and ^{13}C NMR (101 MHz) spectra of compound **aNBDP-3** in $CDCl_3$**.**

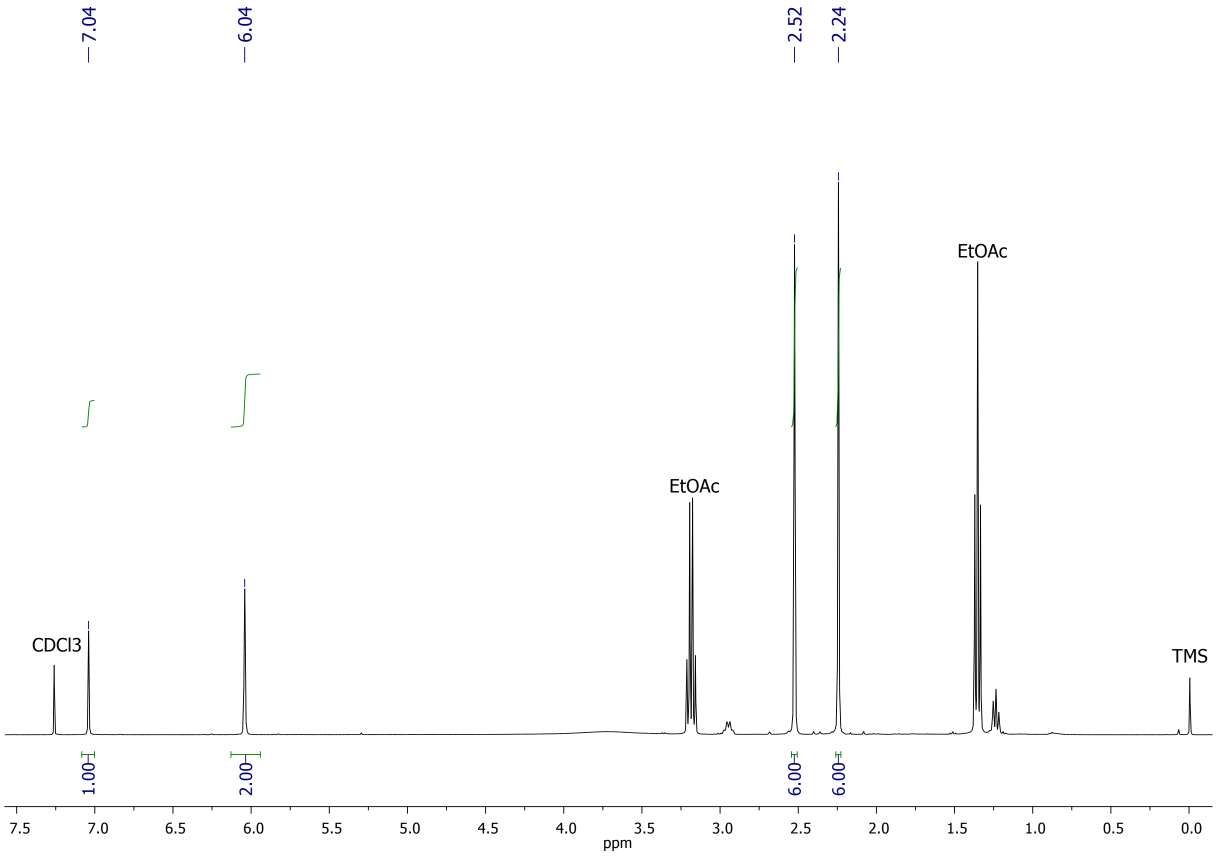

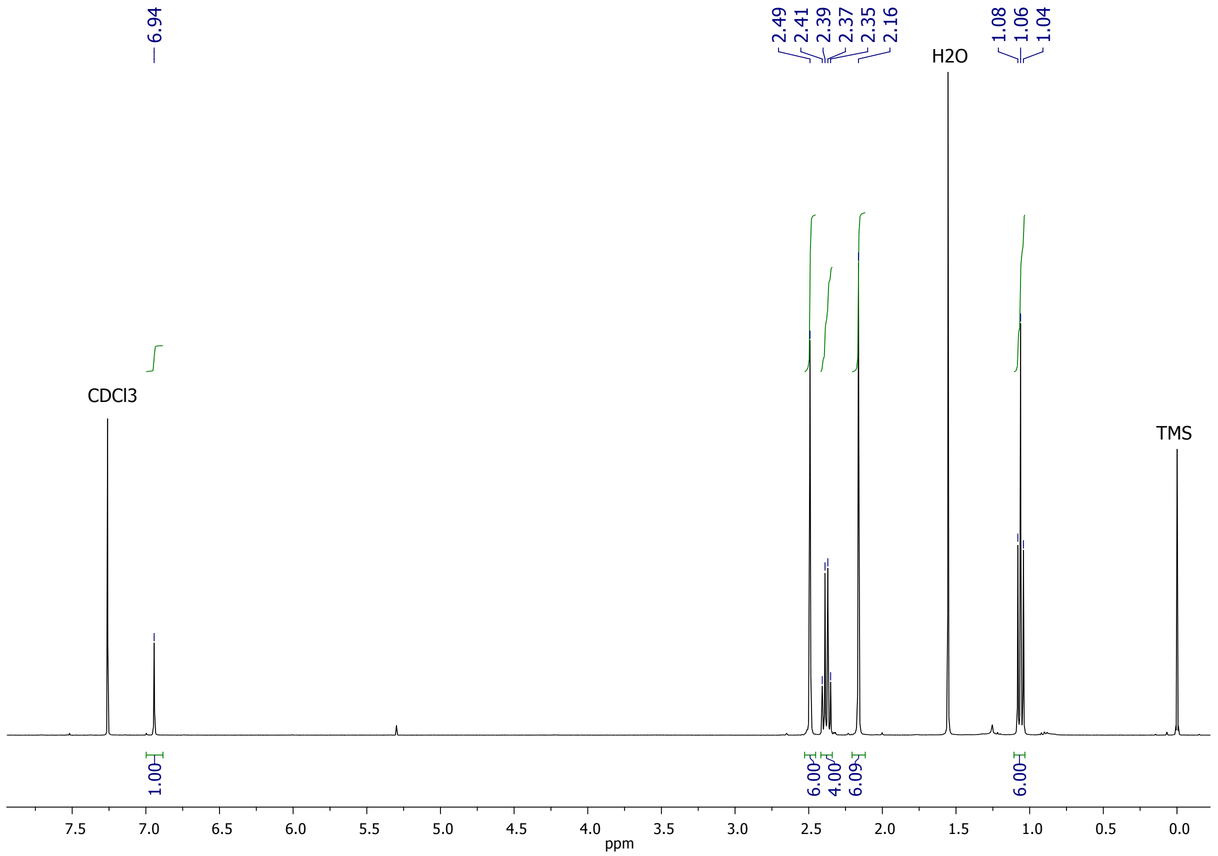

Figure S12: ^{1}H (400 MHz) spectra of compound **sBDP1** and **sBDP2** in $CDCl_3$**.**

4. Optical Properties

4.1 Absorption and emission spectra in different solvents

aBDP-1

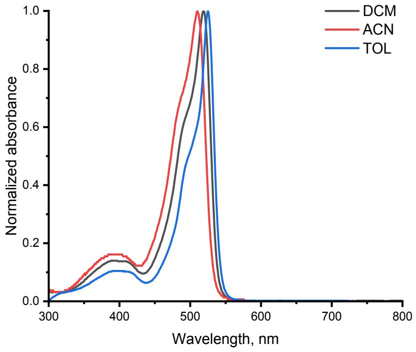

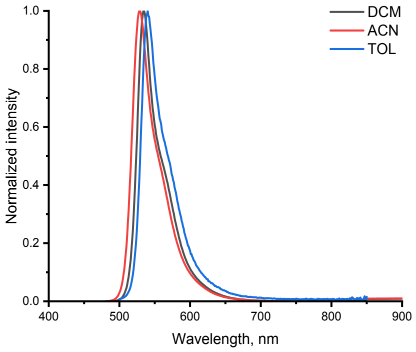

aBDP-2

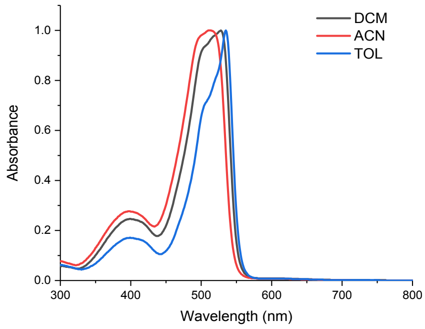

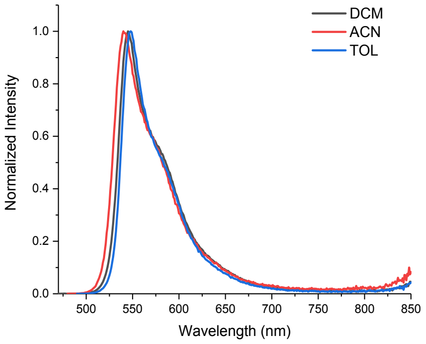

aBDP-3

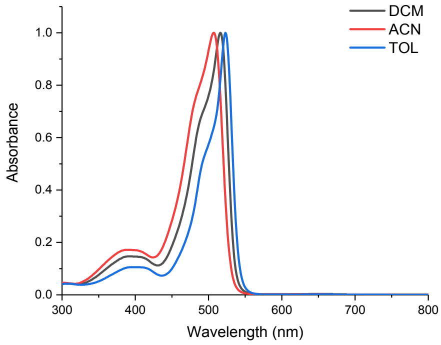

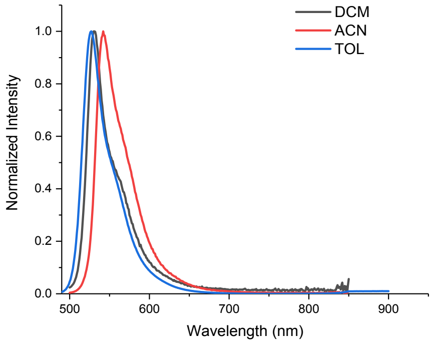

aBDP-4

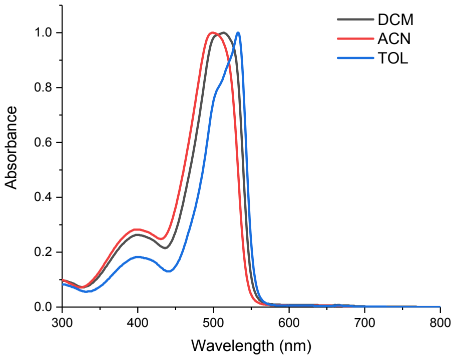

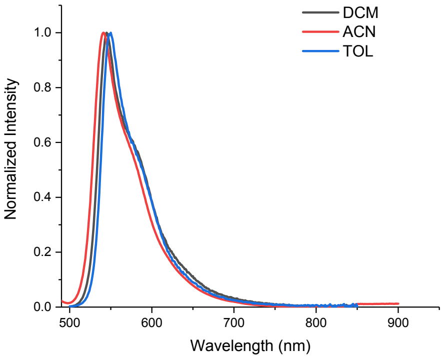

aBDP-5

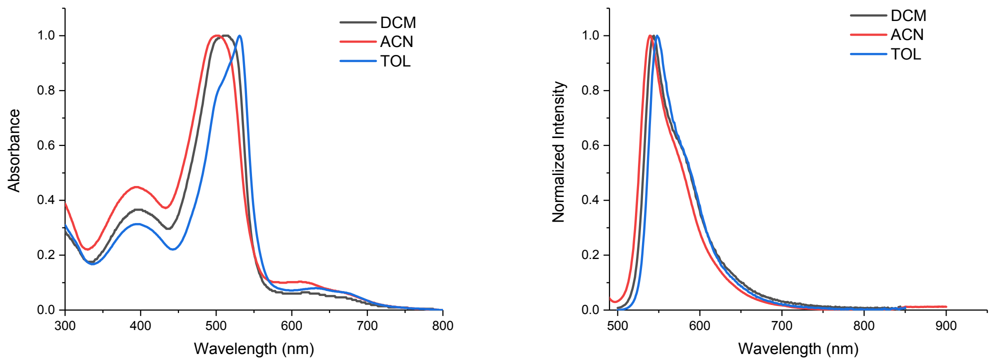

aBDP-6

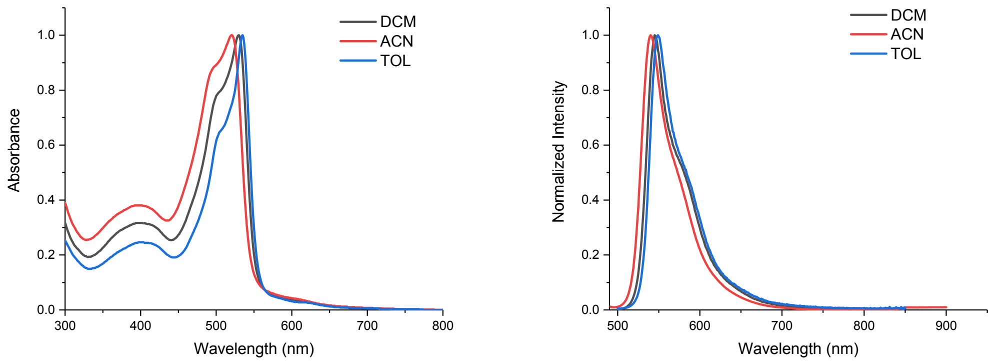

aBBDP-1

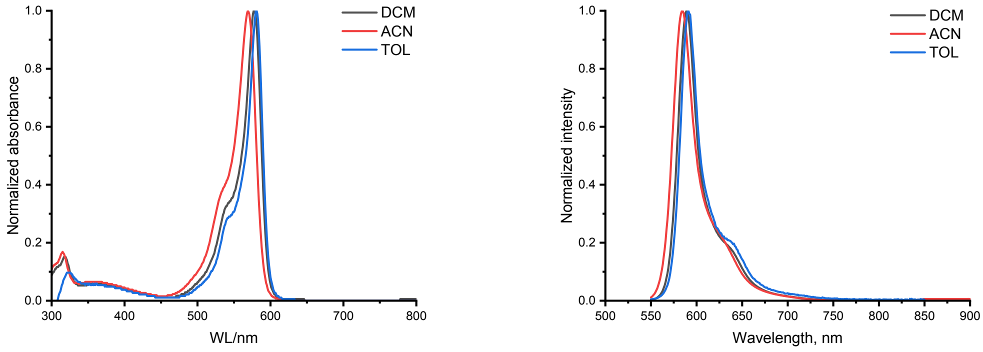

aBBDP-2

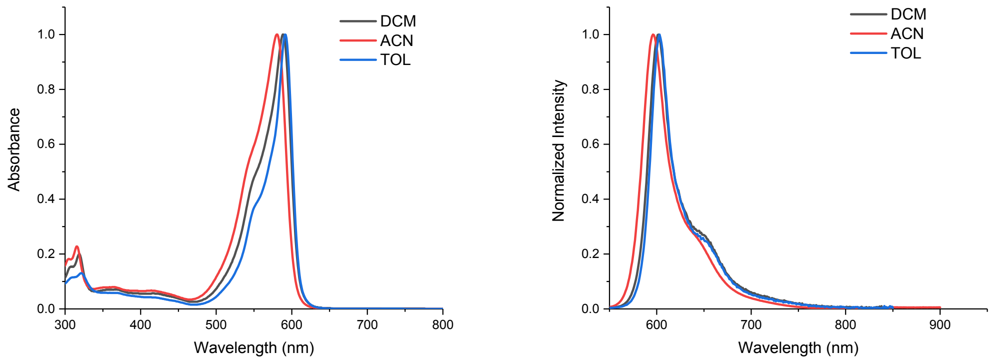

aNBDP-1

aNBDP-2

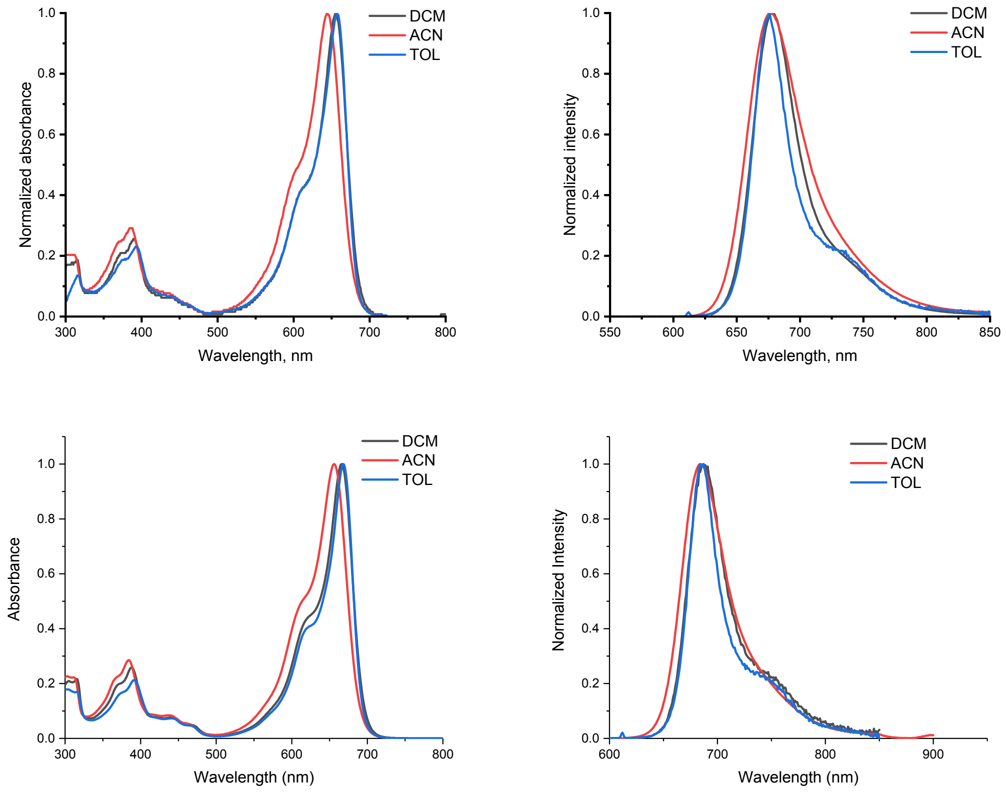

Figure S13: Comparison of absorption and emission spectra of the compounds synthesized in different solvents**.**

Table S1. Optical properties of studied compounds in acetonitrile.

Compound	Absorption λ_{max} (nm)	Fluorescence λ_{max} (nm)	Φ_F	τ, ns
**aBDP-1**	510	527	0.70	5.72
**aBDP-2**	512	540	0.29	3.04
**aBDP-3**	507	542	0.695	5.29
**aBDP-4**	500	541	0.21	2.18
**aBDP-5**	500	539	0.19	3.32
**aBDP-6**	523	540	0.505	4.65
**aBBDP-1**	569	584	0.84	6.62
**aBBDP-2**	581	596	0.38	3.45
**aNBDP-1**	644	677	0.53	6.97
**aNBDP-2**	656	684	0.30	4.12

Table S2. Optical properties of studied compounds in toluene.

Compound	Absorption λ_{max} (nm)	Fluorescence λ_{max} (nm)	Φ_F	τ, ns
**aBDP-1**	525	540	0.76	5.28
**aBDP-2**	535	549	0.65	4.55
**aBDP-3**	523	527	0.795	5.06
**aBDP-4**	532	550	0.56	4.11
**aBDP-5**	530	548	0.51	4.11
**aBDP-6**	534	549	0.65	4.85
**aBBDP-1**	580	591	0.86	5.60
**aBBDP-2**	591	603	0.62	4.69
**aNBDP-1**	657	676	0.60	6.25
**aNBDP-2**	668	687	0.36	4.21

4.2 Singlet oxygen generation quantum yields measurements

4.2.1 Chemical trapping method

The singlet oxygen quantum yield measurements were performed according to previously described method.[13] Solutions of the $^1O_2$ trap, 1,9-dimethylanthracene (DMA), with an optical density of around 1.5 at 378 nm in air-saturated solvent were employed. The corresponding BODIPY was added to the cuvette, and its absorbance was adjusted to 0.05-0.2 at the wavelength of irradiation (520 nm or 635 nm). The solutions in the cuvette were irradiated with a laser at a constant power density. For compounds **aBDP 1-6** and **aBBDP 1-2**, 520 nm (14 mW cm^{-2}) laser was used. For compounds **aNBDP 1-2**, 635 nm (10 mW cm^{-2}) laser was used. The absorption spectra of the solutions were measured every 30 s. The slope of plots of absorbance of DMA at 376 nm vs. irradiation time for each photosensitizer was calculated.

Singlet oxygen quantum yields were calculated based on the equation:

$$\Phi_\Delta = \Phi_\Delta^{ref} \times \frac{k}{k_{ref}} \times \frac{I_{abs}^{ref}}{I_{abs}} \quad \text{(S1)}$$

where Φ_Δ is the singlet oxygen quantum yield; the superscript *ref* stands for 2,6-diiodo-8-phenyl-1,3,5,7-tetramethylBODIPY (Φ_Δ = 0.85 in toluene)[14] or Methylene Blue (Φ_Δ = 0.51 in toluene)[15]; *k* is the slope of the curves of DMA absorption (376 nm) vs. irradiation time; Iabs represents the absorption correction factor which is given by $I = 1–10^{-OD}$ (OD is the optical density at 520 nm). Recorded UV-Vis spectra showing the decrease in DMA absorbance are shown in Figure S14.

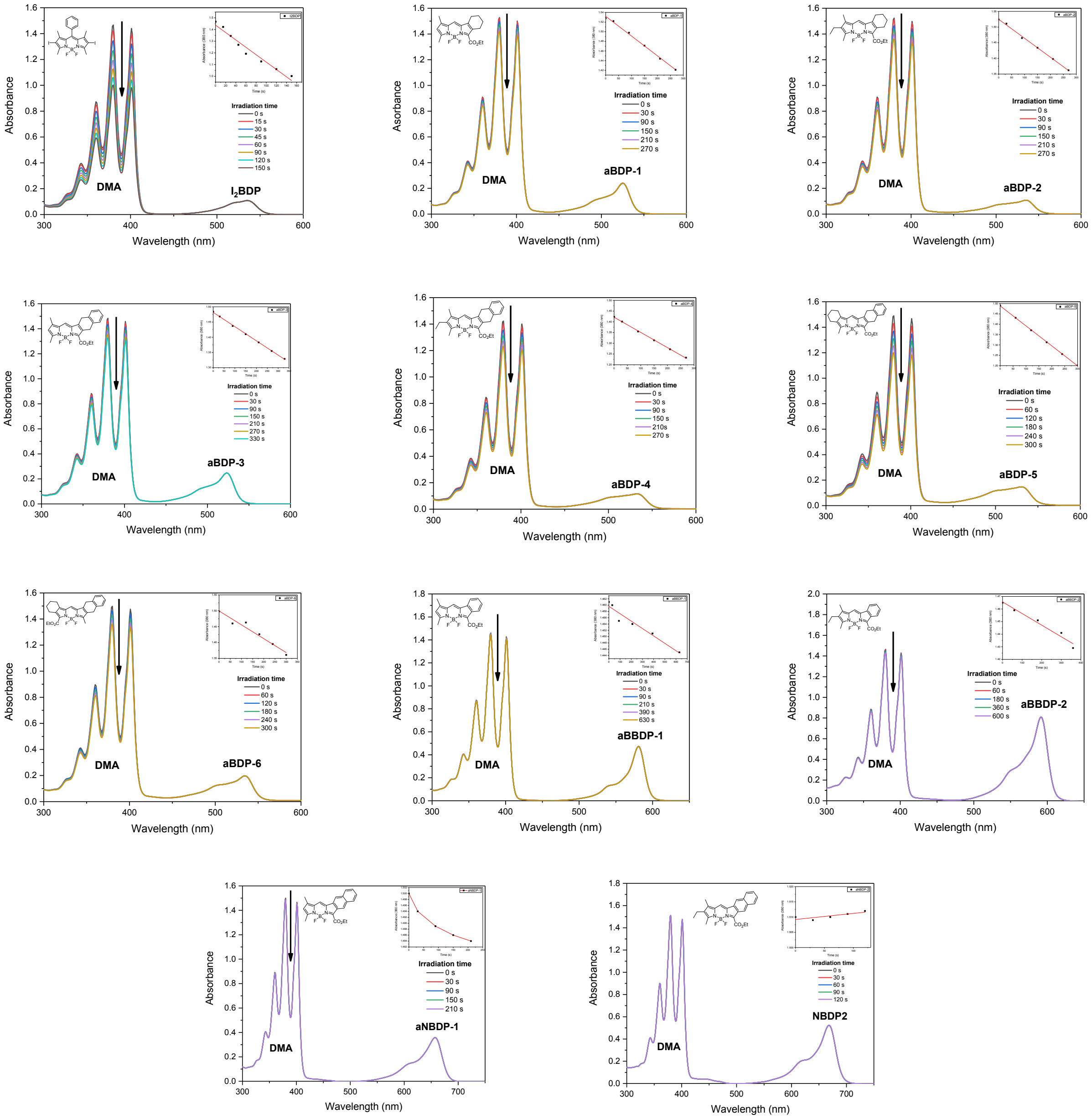

Figure S14. UV-vis spectra of **I_2BDP**, **aBDPs** 1-6, **aBBDPs** 1-2 and **aNBDPs** 1-2 in toluene in the presence of DMA at fixed irradiation times with a laser showing the decrease of absorbance of DMA.

Table S3. Singlet oxygen quantum yields of compounds aBDP-1 and aBDP-2 in different solvents (CYH – cyclohexane, TOL – toluene, DCM – dicholoromethane, CAN – acetonitrile).

Compound	Solvent	Φ_Δ	Compound	Solvent	Φ_Δ
**aBDP-1**	CYH	0.08	**aBDP-2**	CYH	0.27
	TOL	0.06		TOL	0.18
	DCM	0.13		DCM	0.28
	ACN	0.21		ACN	0.25

4.2.2 Singlet oxygen phosphorescence method

Steady state solution phosphorescence emission of $^1O_2$ was acquired on a FluoroMax™ Plus fluorimeter from HORIBA, equipped with 150W ozone-free xenon source and DSS-IGA020L liquid nitrogen cooled InGaAs detector (800-1550 nm at liquid nitrogen temperature). Liquid samples of BODIPY were

prepared in carbon disulfide as a solvent. Absorbance at the excitation wavelength was adjusted to 0.05-0.2. Solutions were transferred into quartz cuvettes and placed in the instrument's sample holder. Samples were excited at 520 nm for **aBDP 1-6**, at 550 nm for **aBBDP 1-2** and at 600 nm for **aNBDP 1-2**. Excitation and emission slits were set to 2 and 29 nm, respectively. Diffuse light, scattering and harmonic phenomena were filtered off from with a long pass filter (500-830 nm) from Edmund Optics placed before the emission monochromator. Singlet oxygen quantum yields were calculated based on the equation:

$$\Phi_\Delta = \Phi_\Delta^{ref} \times \frac{Abs_{Ref}}{Abs_S} \times \frac{Area_S}{Area_{Ref}} \qquad \text{(S2)}$$

where Φ_Δ is the singlet oxygen quantum yield; the superscript *ref* stands for 2,6-diiodo-8-phenyl-1,3,5,7-tetramethylBODIPY (Φ_Δ = 0.85);14 *Abs* is the absorbance of the sample at the excitation wavelength (520 nm); *AreaS and AreaRef* represents the area under the emission peak for the sample and the reference, respectively.

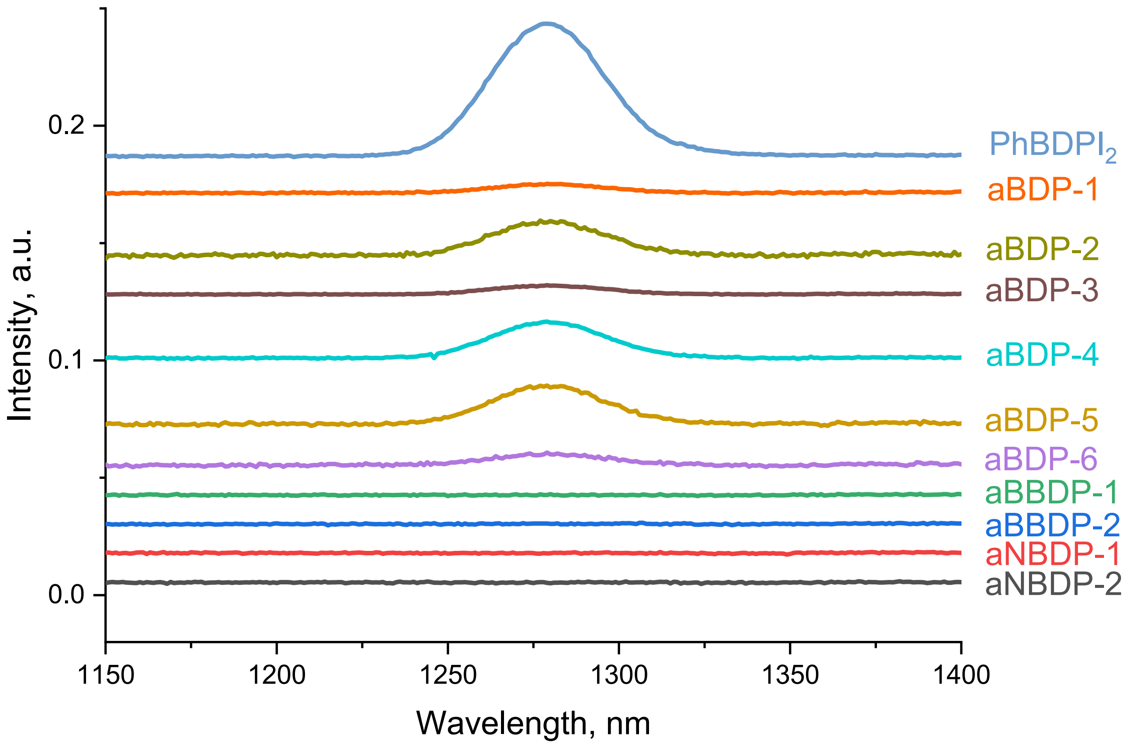

Figure S15: Comparison of singlet oxygen phosphorescence spectra for different BODIPY samples in CS_2 solution. Excitation wavelengths: 520 nm for **PhBDPI2** and **aBDP 1-6**, 550 nm for **aBBDP 1-2**, 600 nm for **aNBDP 1-2**. Absorption at the excitation wavelength was adjusted to ~0.1 for all samples.

4.2 Two-photon absorption measurements

The methods and protocols for obtaining two-photon absorption spectra and cross sections were described previously.[16,17] Briefly, the two-photon absorption spectra were measured as corrected fluorescence excitation spectra by stepping the wavelength of a femtosecond laser (DeepSee Insight, MKS - Spectra-Physics) and collecting fluorescence signal at each wavelength. The power dependence of fluorescence signal was checked for several excitation wavelengths, and only the data points where it was quadratic were selected and presented in Figures 8 and S16. Two-photon absorption cross section, σ^2, was measured at 940 or 1060 nm relatively to rhodamine 6G in methanol comparing one- and two-photon excited fluorescence signals for both the sample and reference standard.

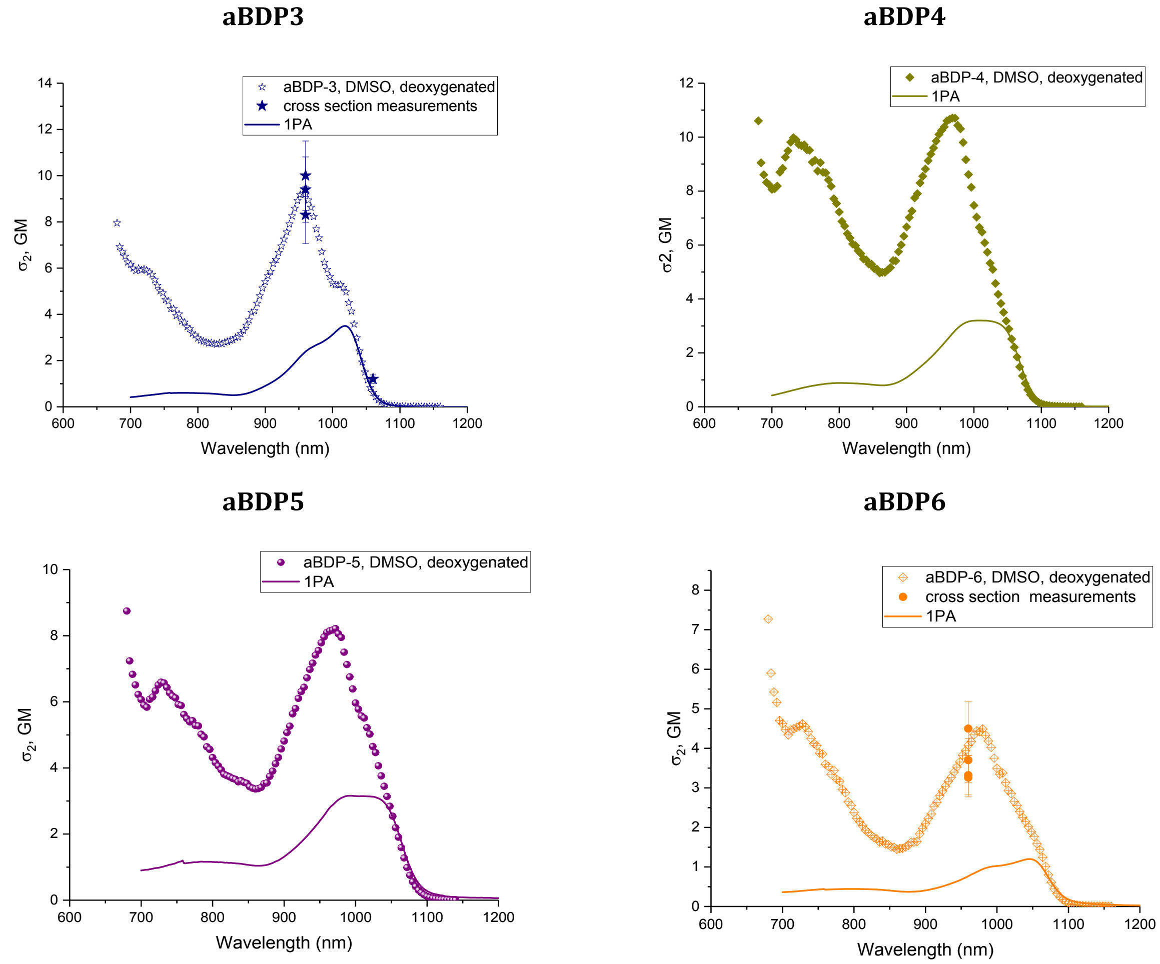

Figure S16: 2PA spectra (data points) of **aBDP 3-6** measured in deoxygenated DMSO. The corresponding 1PA spectra (solid lines) are arbitrarily scaled. The red edge (1300 nm) was determined by the tunability range of the laser.

5. Computational Studies

Density functional theory (DFT) and time-dependent DFT (TDDFT) calculations were performed using Firefly 8.2.0[18] and Gaussian 16[19] software packages. The results were visualized using Chemcraft 1.7 software. All calculations were carried out using B3LYP/6-31G(d,p) model chemistry.

The electric dipole operator matrix was computed by TDDFT for 30 excited states as implemented in Firefly 8.2. The 2PA spectra were computed using a custom code based on SOS formalism with full orientational averaging, as described in previous publications.[20]

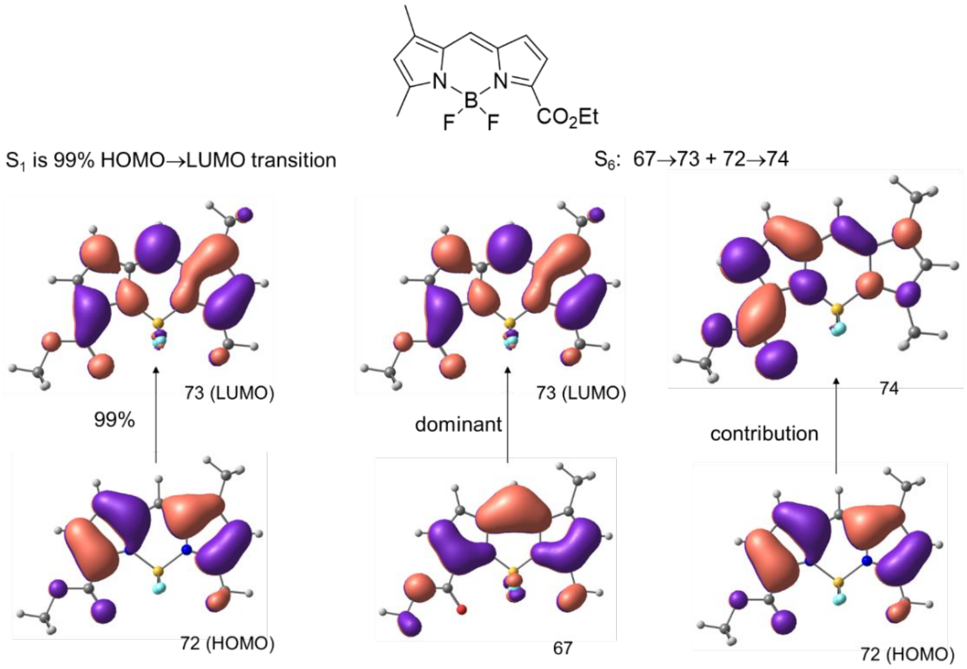

Figure S17: Orbitals involved in 1P (S_1) and 2P (S_6) transitions of 1-ethoxycarbonyl-3,7-dimethylBODIPY.

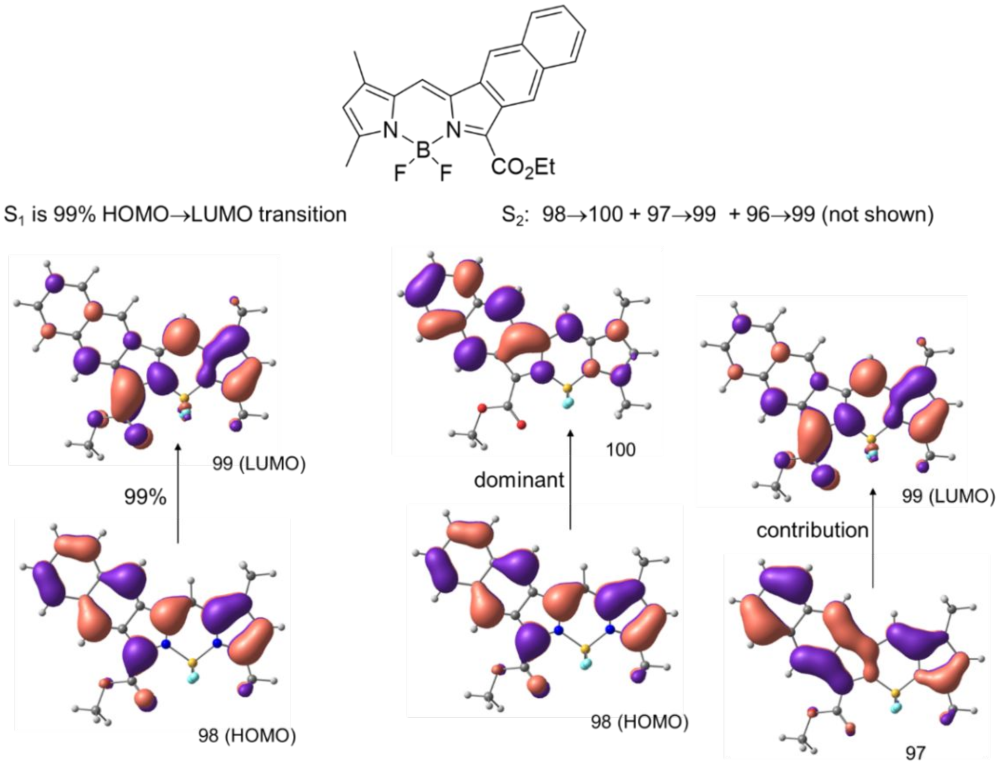

Figure S18: Orbitals involved in 1P (S_1) and 2P (S_2) transitions of **aBBDP-1**.

1-ethoxycarbonyl-3,7-dimethylBODIPY

Optimized geometry:

ATOM	ATOMIC CHARGE	X	Y	Z
			COORDINATES (BOHR)	
CARBON	6.0	-4.1547638976	4.1376225315	0.0001373200
CARBON	6.0	-3.7338705437	1.5031032421	0.0000819368
CARBON	6.0	-1.8014361283	5.2867103662	0.0001070161
CARBON	6.0	0.0152057497	3.3273969364	0.0000497710
NITROGEN	7.0	-1.2089284211	1.0116360089	0.0000244791
CARBON	6.0	2.6644319876	3.5138072489	0.0000507454
CARBON	6.0	4.2035417485	1.4178199843	0.0000420103
NITROGEN	7.0	3.1369315345	-0.9937863901	0.0000637777
CARBON	6.0	5.0241840245	-2.6835221447	0.0000215671
CARBON	6.0	7.3855165789	-1.3913709911	-0.0000034323
CARBON	6.0	6.9071139601	1.1773634800	0.0000015811
BORON	5.0	0.2025825152	-1.6385994403	0.0000300020
FLUORINE	9.0	-0.3253617084	-2.9596749019	-2.1809320491
FLUORINE	9.0	-0.3254671897	-2.9596947514	2.1809550418
CARBON	6.0	-5.7032298758	-0.4821741673	-0.0000248372
HYDROGEN	1.0	3.4984177678	5.3879578308	0.0000562324
CARBON	6.0	4.5709047911	-5.4663966306	0.0000153160
HYDROGEN	1.0	9.2140532407	-2.3088648278	-0.0000384072
CARBON	6.0	8.7802507202	3.3006931056	-0.0002549055
HYDROGEN	1.0	3.4560934831	-6.0074824066	-1.6573448977
HYDROGEN	1.0	6.3566904460	-6.5025870822	-0.0002634656
HYDROGEN	1.0	3.4565700828	-6.0075858043	1.6576664590
HYDROGEN	1.0	10.0063581871	3.2144463186	-1.6687125656
HYDROGEN	1.0	7.8592144900	5.1513809228	0.0022477092
HYDROGEN	1.0	10.0099151460	3.2116795063	1.6654129152
OXYGEN	8.0	-8.0327330786	0.6018061396	0.0003075905
OXYGEN	8.0	-5.3639794263	-2.7442224820	-0.0004077815
CARBON	6.0	-10.0993828454	-1.1524546524	-0.0000352910
HYDROGEN	1.0	-10.0345252047	-2.3539865419	1.6798746196
HYDROGEN	1.0	-10.0350445598	-2.3526849405	-1.6809043356
HYDROGEN	1.0	-11.8046345142	0.0047663921	0.0006745413
HYDROGEN	1.0	-5.9925570421	5.0231360968	0.0001774959
HYDROGEN	1.0	-1.3801199067	7.2880446749	0.0001150376

Imaginary frequencies: 1.

DFT REFERENCE ENERGY = -987.8908411767

TDDFT EXCITATION ENERGIES

STATE	HARTREE	EV	KCAL/MOL	CM-1	NANOMETERS	OSC. STR.
1A	0.1099833503	2.9928	69.0156	24138.56	414.28	0.4676164
1A	0.1244795860	3.3873	78.1121	27320.11	366.03	0.1666928
1A	0.1308076884	3.5595	82.0831	28708.97	348.32	0.0000406
1A	0.1320612555	3.5936	82.8697	28984.10	345.02	0.0451218
1A	0.1706191474	4.6428	107.0651	37446.57	267.05	0.0099835
1A	0.1773286262	4.8254	111.2754	38919.13	256.94	0.0047275
1A	0.1810994154	4.9280	113.6416	39746.73	251.59	0.0000531
1A	0.1955069427	5.3200	122.6825	42908.81	233.05	0.1671114
1A	0.2017592332	5.4901	126.6058	44281.03	225.83	0.1497893
1A	0.2028514613	5.5199	127.2912	44520.75	224.61	0.0002233
1A	0.2086732134	5.6783	130.9444	45798.48	218.35	0.0013307
1A	0.2128974593	5.7932	133.5952	46725.59	214.02	0.0003869
1A	0.2202550140	5.9934	138.2121	48340.39	206.87	0.0242956
1A	0.2237491730	6.0885	140.4047	49107.27	203.64	0.0001976
1A	0.2241593141	6.0997	140.6621	49197.28	203.26	0.0091374
1A	0.2329079022	6.3377	146.1519	51117.38	195.63	0.0000315
1A	0.2376388542	6.4665	149.1207	52155.70	191.73	0.1953524
1A	0.2401069483	6.5336	150.6694	52697.38	189.76	0.0008175
1A	0.2483349390	6.7575	155.8325	54503.22	183.48	0.0181212
1A	0.2502511542	6.8097	157.0350	54923.78	182.07	0.0265646
1A	0.2516250725	6.8471	157.8971	55225.32	181.08	0.0000052
1A	0.2603375269	7.0841	163.3643	57137.48	175.02	0.0122891
1A	0.2620897550	7.1318	164.4638	57522.05	173.85	0.0532889
1A	0.2647115753	7.2032	166.1090	58097.48	172.12	0.0000003
1A	0.2655651844	7.2264	166.6447	58284.82	171.57	0.0001072

1A	0.2673343743	7.2745	167.7549	58673.11	170.44	0.0725622
1A	0.2713620738	7.3841	170.2823	59557.09	167.91	0.0005046
1A	0.2732882301	7.4366	171.4910	59979.83	166.72	0.0907894
1A	0.2738094172	7.4507	171.8180	60094.22	166.41	0.0014356
1A	0.2758547642	7.5064	173.1015	60543.12	165.17	0.0002369

aBBDP-1

Optimized geometry:

ATOM	ATOMIC CHARGE	COORDINATES (BOHR) X	Y	Z
CARBON	6.0	-4.2904364911	1.6496902765	0.0001253183
CARBON	6.0	-2.9849628462	-0.7258331326	0.0002060979
CARBON	6.0	-2.3883136655	3.5526082074	0.0002273539
CARBON	6.0	-0.0115829130	2.2642082265	0.0002827057
NITROGEN	7.0	-0.4558426995	-0.3192162759	0.0003021307
CARBON	6.0	2.4243932628	3.2727201192	0.0001235240
CARBON	6.0	4.5695359500	1.7811674964	-0.0001115776
NITROGEN	7.0	4.3337446280	-0.8375420263	-0.0001498638
CARBON	6.0	6.6735568940	-1.8380192604	-0.0004347969
CARBON	6.0	8.4825302073	0.1397295560	-0.0006286796
CARBON	6.0	7.1949444580	2.4244542953	-0.0004229714
BORON	5.0	1.7833540876	-2.3841259415	0.0004583880
FLUORINE	9.0	1.6779845354	-3.7890676865	-2.1853823883
FLUORINE	9.0	1.6785401158	-3.7882060077	2.1868657149
CARBON	6.0	-6.8658764001	2.3458245563	0.0000294206
CARBON	6.0	-4.0941342639	-3.2975135758	0.0002309300
CARBON	6.0	-3.0330849981	6.1282013762	0.0002286363
HYDROGEN	1.0	2.6195473472	5.3143562570	0.0001169300
CARBON	6.0	7.1314528421	-4.6206789556	-0.0003668145
HYDROGEN	1.0	10.5097706533	-0.1355708250	-0.0009033174
CARBON	6.0	8.2837567214	5.0387347974	-0.0005780899
CARBON	6.0	-7.4681611258	4.8902359089	0.0000407200
CARBON	6.0	-5.5686555507	6.7721647390	0.0001414177
HYDROGEN	1.0	-8.3256489117	0.9139097732	-0.0000627654
HYDROGEN	1.0	-1.5845293369	7.5840593039	0.0003001877
HYDROGEN	1.0	6.2524216731	-5.4934849235	-1.6584097559
HYDROGEN	1.0	9.1553165683	-5.0303153404	-0.0017049020
HYDROGEN	1.0	6.2548052669	-5.4929987488	1.6592188654
HYDROGEN	1.0	9.4732924459	5.3556362063	-1.6681274078
HYDROGEN	1.0	6.8137605740	6.4928907064	0.0002153708
HYDROGEN	1.0	9.4746948034	5.3552287706	1.6660405700
HYDROGEN	1.0	-9.4400846332	5.4621233434	-0.0000421193
HYDROGEN	1.0	-6.1151893919	8.7515037740	0.0001582302
OXYGEN	8.0	-6.6609747395	-3.1693664662	-0.0008852456
OXYGEN	8.0	-2.9455123950	-5.2743147836	0.0011270835
CARBON	6.0	-7.9184169003	-5.5737037418	-0.0011305940
HYDROGEN	1.0	-7.4070998497	-6.6612875206	1.6791957987
HYDROGEN	1.0	-7.4085836651	-6.6600847575	-1.6827125032
HYDROGEN	1.0	-9.9330761962	-5.1398302564	-0.0001427306

Imaginary frequencies: 2

DFT REFERENCE ENERGY = -1141.5350574096

TDDFT EXCITATION ENERGIES

STATE	HARTREE	EV	KCAL/MOL	CM-1	NANOMETERS	OSC. STR.
1A	0.0953708704	2.5952	59.8461	20931.49	477.75	0.5254684
1A	0.1185852871	3.2269	74.4134	26026.46	384.22	0.0698308
1A	0.1270648684	3.4576	79.7344	27887.52	358.58	0.0281041
1A	0.1315105866	3.5786	82.5242	28863.24	346.46	0.0000691
1A	0.1394481546	3.7946	87.5051	30605.33	326.74	0.0249932
1A	0.1510381124	4.1100	94.7779	33149.03	301.67	0.2398746
1A	0.1724497397	4.6926	108.2139	37848.34	264.21	0.0096460
1A	0.1757062649	4.7812	110.2574	38563.07	259.32	0.0146270
1A	0.1824442398	4.9646	114.4855	40041.88	249.74	0.0000012
1A	0.1848072160	5.0289	115.9683	40560.50	246.55	0.1429763
1A	0.1953127907	5.3147	122.5606	42866.20	233.28	0.0781481

1A	0.1982883841	5.3957	124.4279	43519.27	229.78	0.1222945
1A	0.2020442265	5.4979	126.7847	44343.58	225.51	0.1725087
1A	0.2033278665	5.5328	127.5902	44625.31	224.09	0.0001450
1A	0.2040436181	5.5523	128.0393	44782.40	223.30	0.0003686
1A	0.2080888604	5.6624	130.5777	45670.23	218.96	0.0046759
1A	0.2111169662	5.7448	132.4779	46334.82	215.82	0.0238558
1A	0.2119234043	5.7667	132.9840	46511.81	215.00	0.0013953
1A	0.2226060302	6.0574	139.6874	48856.38	204.68	0.0998664
1A	0.2259117229	6.1474	141.7618	49581.89	201.69	0.0002165
1A	0.2267242777	6.1695	142.2717	49760.23	200.96	0.0317675
1A	0.2275194342	6.1911	142.7706	49934.74	200.26	0.0000166
1A	0.2291511892	6.2355	143.7946	50292.87	198.84	0.0049387
1A	0.2330132762	6.3406	146.2181	51140.50	195.54	0.0003273
1A	0.2334264084	6.3519	146.4773	51231.17	195.19	0.0266090
1A	0.2355241122	6.4089	147.7936	51691.57	193.46	0.0357635
1A	0.2413878386	6.5685	151.4732	52978.51	188.76	0.0000402
1A	0.2417053614	6.5771	151.6724	53048.20	188.51	0.0766538
1A	0.2447290924	6.6594	153.5698	53711.83	186.18	0.2112921
1A	0.2457119419	6.6862	154.1866	53927.54	185.43	0.0006259

aBBDP-2

Optimized geometry:

ATOM	ATOMIC CHARGE	COORDINATES (BOHR) X	Y	Z
CARBON	6.0	2.9980049878	0.9013168451	0.0576511335
CARBON	6.0	0.7657873409	2.4319769088	0.0572899029
CARBON	6.0	2.1583796901	-1.6564404198	0.1028803393
CARBON	6.0	-0.5425104733	-1.5782569679	0.1018148116
NITROGEN	7.0	-1.3078717262	0.9254232195	0.0899297854
CARBON	6.0	-2.2680336262	-3.5773025422	0.0427099439
CARBON	6.0	-4.8501050787	-3.2113512539	-0.0661460911
NITROGEN	7.0	-5.8073818808	-0.7679413924	-0.0893136402
CARBON	6.0	-8.3340819183	-0.9255561618	-0.2229616216
CARBON	6.0	-9.1094856256	-3.5193129455	-0.3022153841
CARBON	6.0	-6.9096619833	-4.9664717414	-0.2018491440
BORON	5.0	-4.2258698842	1.7562370984	0.1159738151
FLUORINE	9.0	-4.7430734136	3.2175960126	-1.9734190399
FLUORINE	9.0	-4.8027421434	2.8893319708	2.3857214116
CARBON	6.0	5.6101910743	1.4446051938	0.0444030176
CARBON	6.0	0.5923428687	5.2241389373	0.0736052653
CARBON	6.0	3.9002737219	-3.6617339994	0.1294369232
HYDROGEN	1.0	-1.5179535736	-5.4854419285	0.0576820343
CARBON	6.0	-9.9619903649	1.3797769921	-0.2954780267
CARBON	6.0	-6.5549356134	-7.7837737396	-0.2413272311
CARBON	6.0	7.2972122422	-0.5516131593	0.0747637657
CARBON	6.0	6.4522972969	-3.0893766399	0.1163994570
HYDROGEN	1.0	6.2636139693	3.3824236757	0.0050284195
HYDROGEN	1.0	3.2675929521	-5.6154970000	0.1647286906
HYDROGEN	1.0	-9.7094226800	2.3844190218	-2.0893737423
HYDROGEN	1.0	-11.9568999863	0.9011946232	-0.0752554743
HYDROGEN	1.0	-9.4023673633	2.6916673834	1.2015262282
HYDROGEN	1.0	-7.5303551620	-8.7099537869	1.3310411990
HYDROGEN	1.0	-7.2726382871	-8.6293842005	-1.9898513046
HYDROGEN	1.0	-4.5542247123	-8.2851785217	-0.1005706405
OXYGEN	8.0	2.8860386071	6.2736844087	-0.4222200248
OXYGEN	8.0	-1.2841219941	6.4661586251	0.4825463812
CARBON	6.0	2.9115585780	8.9869331220	-0.4104343283
HYDROGEN	1.0	2.3518681151	9.7123070615	1.4416287410
HYDROGEN	1.0	1.6151078821	9.7280696886	-1.8375891788
HYDROGEN	1.0	4.8540566963	9.5209482049	-0.8447146098
CARBON	6.0	-11.8681955428	-4.2453133255	-0.4798861929
HYDROGEN	1.0	-12.8920697650	-3.3125332982	1.0693607994
HYDROGEN	1.0	-12.6594822300	-3.4216685441	-2.2169405378
CARBON	6.0	-12.5278670705	-7.0650308358	-0.4257603605
HYDROGEN	1.0	-11.9093430649	-7.9679713865	1.3297061699
HYDROGEN	1.0	-11.6832654701	-8.0903567154	-2.0107361042
HYDROGEN	1.0	-14.5800756220	-7.3006020840	-0.5566082327
HYDROGEN	1.0	7.8347436462	-4.6074682183	0.1391818421
HYDROGEN	1.0	9.3147019158	-0.1709077152	0.0643566962

Imaginary frequencies: 0

DFT REFERENCE ENERGY = -1220.1614961965

TDDFT EXCITATION ENERGIES

STATE	HARTREE	EV	KCAL/MOL	CM-1	NANOMETERS	OSC. STR.
1A	0.0937041435	2.5498	58.8002	20565.68	486.25	0.5490086
1A	0.1165520528	3.1715	73.1375	25580.22	390.93	0.0836127
1A	0.1272206094	3.4618	79.8321	27921.70	358.14	0.0293022
1A	0.1334249455	3.6307	83.7254	29283.39	341.49	0.0006246
1A	0.1397113363	3.8017	87.6702	30663.09	326.12	0.0264047
1A	0.1498120104	4.0766	94.0085	32879.94	304.14	0.2508030
1A	0.1712760672	4.6607	107.4774	37590.75	266.02	0.0181971
1A	0.1763920372	4.7999	110.6877	38713.58	258.31	0.0149871
1A	0.1828449621	4.9755	114.7370	40129.83	249.19	0.0424277
1A	0.1843521830	5.0165	115.6828	40460.63	247.15	0.0858401
1A	0.1915217283	5.2116	120.1817	42034.16	237.90	0.0248111
1A	0.1976841168	5.3793	124.0487	43386.65	230.49	0.2219770
1A	0.2037176614	5.5434	127.8348	44710.86	223.66	0.0149171
1A	0.2039138388	5.5488	127.9579	44753.91	223.44	0.0807098
1A	0.2056912776	5.5971	129.0732	45144.02	221.51	0.0006640
1A	0.2069561626	5.6316	129.8670	45421.63	220.16	0.0358771
1A	0.2110122146	5.7419	132.4122	46311.83	215.93	0.0007099
1A	0.2117787714	5.7628	132.8932	46480.07	215.15	0.0335328
1A	0.2185377376	5.9467	137.1345	47963.49	208.49	0.1337961
1A	0.2190271738	5.9600	137.4416	48070.91	208.03	0.0031996

aNBDP-1

Optimized geometry:

ATOM	ATOMIC CHARGE	COORDINATES (BOHR) X	Y	Z
CARBON	6.0	2.9932254454	0.8749325719	0.0488444672
CARBON	6.0	0.7576340765	2.4195243481	0.0949596931
CARBON	6.0	2.1311466763	-1.6986854618	0.1041908408
CARBON	6.0	-0.5818959184	-1.5899908385	0.1441482488
NITROGEN	7.0	-1.3123268810	0.9312855521	0.1605774723
CARBON	6.0	-2.3377630984	-3.5463541357	0.0756859470
CARBON	6.0	-4.9208411003	-3.1292675862	-0.0314405267
NITROGEN	7.0	-5.8510507425	-0.6698349807	-0.0570562020
CARBON	6.0	-8.3922641322	-0.7855242237	-0.1992429966
CARBON	6.0	-9.1504354384	-3.3530212984	-0.2815327369
CARBON	6.0	-6.9969680716	-4.8478502548	-0.1754375646
BORON	5.0	-4.2385813083	1.8095228861	0.2256896646
FLUORINE	9.0	-4.6976237678	3.3612051055	-1.8122608812
FLUORINE	9.0	-4.7699823636	2.8840636982	2.5308380197
CARBON	6.0	5.5670627981	1.4129586504	-0.0025817229
CARBON	6.0	0.6168516930	5.2185461192	0.1407029836
CARBON	6.0	3.8298348335	-3.6907870770	0.0995245654
HYDROGEN	1.0	-1.6381186153	-5.4749499776	0.0708256313
CARBON	6.0	-10.0166143827	1.5204154608	-0.2457578433
HYDROGEN	1.0	-11.0879120283	-3.9980504546	-0.4081122113
CARBON	6.0	-6.8320707011	-7.6746633668	-0.2155662130
CARBON	6.0	7.3177295466	-0.5894033443	-0.0048411516
CARBON	6.0	6.4428393853	-3.1795173926	0.0472455119
HYDROGEN	1.0	6.2311262795	3.3488802949	-0.0492244701
HYDROGEN	1.0	3.1897497904	-5.6430964733	0.1420010518
HYDROGEN	1.0	-9.5728444634	2.6913035637	-1.8938009275
HYDROGEN	1.0	-12.0151104993	1.0024900566	-0.2959154220
HYDROGEN	1.0	-9.6564429141	2.6884225436	1.4252473290
HYDROGEN	1.0	-8.7180520870	-8.5186514279	-0.2703828170
HYDROGEN	1.0	-5.7908209951	-8.3600900683	-1.8733739455
HYDROGEN	1.0	-5.8626511273	-8.4112438120	1.4629762214
CARBON	6.0	9.9765112765	-0.1278555437	-0.0584763595
CARBON	6.0	8.2756034000	-5.1567282534	0.0464210639
OXYGEN	8.0	2.8506234180	6.2346584922	-0.6186823760
OXYGEN	8.0	-1.1792522592	6.4771980754	0.7834389198

CARBON	6.0	11.6771466067	-2.0829864259	-0.0581809074
CARBON	6.0	10.8137879053	-4.6294615626	-0.0041499909
HYDROGEN	1.0	10.6266018056	1.8209341114	-0.0998271196
HYDROGEN	1.0	7.6164277619	-7.1027525621	0.0869642091
HYDROGEN	1.0	13.6934297012	-1.6987954173	-0.0990099635
HYDROGEN	1.0	12.1818251590	-6.1604896934	-0.0031646087
CARBON	6.0	2.9332406341	8.9480934325	-0.5792919905
HYDROGEN	1.0	2.6110866550	9.6569789954	1.3345723417
HYDROGEN	1.0	1.4943062280	9.7333392297	-1.8357676119
HYDROGEN	1.0	4.8217374308	9.4494301751	-1.2339942137

Imaginary frequencies: 0

DFT REFERENCE ENERGY = -1295.1749291267

TDDFT EXCITATION ENERGIES

STATE	HARTREE	EV	KCAL/MOL	CM-1	NANOMETERS	OSC. STR.
1A	0.0813103558	2.2126	51.0230	17845.56	560.36	0.4265711
1A	0.1124478657	3.0599	70.5621	24679.45	405.20	0.0254169
1A	0.1172769426	3.1913	73.5924	25739.31	388.51	0.0004881
1A	0.1216527068	3.3103	76.3382	26699.68	374.54	0.2304697
1A	0.1270976183	3.4585	79.7550	27894.70	358.49	0.2156265
1A	0.1335976812	3.6354	83.8338	29321.30	341.05	0.0072400
1A	0.1508295726	4.1043	94.6470	33103.26	302.09	0.0970861
1A	0.1611833830	4.3860	101.1441	35375.66	282.68	0.0702822
1A	0.1644237603	4.4742	103.1775	36086.84	277.11	0.0211605
1A	0.1707355800	4.6460	107.1382	37472.13	266.87	0.3777985
1A	0.1735368928	4.7222	108.8961	38086.95	262.56	0.0211790
1A	0.1758848186	4.7861	110.3694	38602.26	259.05	0.0302585
1A	0.1832093758	4.9854	114.9656	40209.81	248.70	0.0272800
1A	0.1854438141	5.0462	116.3678	40700.21	245.70	0.0648727
1A	0.1869182336	5.0863	117.2930	41023.81	243.76	0.2361847
1A	0.1932501157	5.2586	121.2663	42413.50	235.77	0.0002672
1A	0.1942694305	5.2863	121.9059	42637.21	234.54	0.1289206
1A	0.1977386993	5.3807	124.0829	43398.63	230.42	0.0496130
1A	0.2022579046	5.5037	126.9188	44390.48	225.27	0.0027090
1A	0.2036098410	5.5405	127.7671	44687.19	223.78	0.0029073
1A	0.2068177711	5.6278	129.7801	45391.25	220.31	0.1840266
1A	0.2113791901	5.7519	132.6425	46392.37	215.55	0.1051361
1A	0.2120509045	5.7702	133.0640	46539.79	214.87	0.0074671
1A	0.2131817058	5.8010	133.7736	46787.98	213.73	0.0029895
1A	0.2157190543	5.8700	135.3658	47344.86	211.22	0.0443474
1A	0.2175643174	5.9202	136.5237	47749.85	209.42	0.1048108
1A	0.2181205816	5.9354	136.8728	47871.93	208.89	0.0647884
1A	0.2230730428	6.0701	139.9805	48958.87	204.25	0.0000190
1A	0.2234686235	6.0809	140.2287	49045.69	203.89	0.0014686
1A	0.2248403084	6.1182	141.0894	49346.74	202.65	0.0039163

aNBDP-2

Optimized geometry:

ATOM	ATOMIC CHARGE	COORDINATES (BOHR) X	Y	Z
CARBON	6.0	3.0111406026	0.8768689347	0.0712589228
CARBON	6.0	0.7636372124	2.4030618805	0.1043829588
CARBON	6.0	2.1693671758	-1.7042154553	0.1271784927
CARBON	6.0	-0.5436715739	-1.6185750212	0.1593254307
NITROGEN	7.0	-1.2949056747	0.8922844191	0.1654032416
CARBON	6.0	-2.2780483545	-3.5994483428	0.0899334515
CARBON	6.0	-4.8648803578	-3.2182143820	-0.0192819056
NITROGEN	7.0	-5.8103273799	-0.7728108179	-0.0248002687
CARBON	6.0	-8.3424151475	-0.9175878878	-0.1857284800
CARBON	6.0	-9.1233109016	-3.5009050327	-0.3085752763
CARBON	6.0	-6.9257270270	-4.9599153140	-0.1943337032
BORON	5.0	-4.2255822471	1.7329527434	0.2122671078
FLUORINE	9.0	-4.7159324697	3.2301104139	-1.8582994452
FLUORINE	9.0	-4.7739461093	2.8469137250	2.4964345806
CARBON	6.0	5.5823318341	1.4321041623	0.0229504403
CARBON	6.0	0.5881633141	5.1970356745	0.1203782735

CARBON	6.0	3.8836689195	-3.6841594568	0.1266344556
HYDROGEN	1.0	-1.5405954783	-5.5125674711	0.0816929095
CARBON	6.0	-9.9563850271	1.3985827830	-0.2325365804
CARBON	6.0	-6.5835547011	-7.7783001781	-0.2521860963
CARBON	6.0	7.3470669647	-0.5572650202	0.0237007448
CARBON	6.0	6.4922563117	-3.1546730875	0.0760278640
HYDROGEN	1.0	6.2328773622	3.3726125956	-0.0239757075
HYDROGEN	1.0	3.2563427360	-5.6408424755	0.1701126035
HYDROGEN	1.0	-9.6867194309	2.4360409613	-2.0051485317
HYDROGEN	1.0	-11.9554490300	0.9265532587	-0.0360767388
HYDROGEN	1.0	-9.4055819254	2.6797189456	1.2946386753
HYDROGEN	1.0	-7.6242144799	-8.7150511096	1.2707305142
HYDROGEN	1.0	-7.2358052630	-8.6037060387	-2.0360022697
HYDROGEN	1.0	-4.5925371067	-8.2912218545	-0.0344932031
CARBON	6.0	10.0028882788	-0.0757476460	-0.0274843336
CARBON	6.0	8.3412306256	-5.1178644042	0.0763248817
OXYGEN	8.0	2.8463306594	6.2399489253	-0.5282948044
OXYGEN	8.0	-1.2564729803	6.4404128320	0.6485790829
CARBON	6.0	11.7184969968	-2.0173962379	-0.0235410705
CARBON	6.0	10.8750153067	-4.5709716088	0.0296448726
HYDROGEN	1.0	10.6381573441	1.8779259178	-0.0694573600
HYDROGEN	1.0	7.6981750628	-7.0694684324	0.1131348531
HYDROGEN	1.0	13.7317196479	-1.6170422247	-0.0616778299
HYDROGEN	1.0	12.2552547299	-6.0912672164	0.0318048825
CARBON	6.0	2.8780177931	8.9540263119	-0.5290819396
HYDROGEN	1.0	2.4095784763	9.6878080870	1.3444407103
HYDROGEN	1.0	1.5160820153	9.6909063497	-1.8960127057
HYDROGEN	1.0	4.7977772357	9.4826593527	-1.0597360387
CARBON	6.0	-11.8788299652	-4.2208864365	-0.5583118397
HYDROGEN	1.0	-12.9484384188	-3.2621929992	0.9428454154
HYDROGEN	1.0	-12.6138375527	-3.4235766006	-2.3324195791
CARBON	6.0	-12.5496539592	-7.0377793637	-0.4804558679
HYDROGEN	1.0	-11.9811952975	-7.9162228258	1.3043214071
HYDROGEN	1.0	-11.6663403649	-8.0890615160	-2.0267442973
HYDROGEN	1.0	-14.5984578289	-7.2692519830	-0.6627065961

Imaginary frequencies: 0

DFT REFERENCE ENERGY = -1373.8007795493

TDDFT EXCITATION ENERGIES

STATE	HARTREE	EV	KCAL/MOL	CM-1	NANOMETERS	OSC. STR.
1A	0.0803546305	2.1866	50.4233	17635.80	567.03	0.4550709
1A	0.1110132091	3.0208	69.6619	24364.58	410.43	0.0008993
1A	0.1124286294	3.0593	70.5500	24675.23	405.26	0.0293476
1A	0.1220315815	3.3206	76.5760	26782.84	373.37	0.2635784
1A	0.1270039905	3.4560	79.6962	27874.15	358.76	0.2205535
1A	0.1334824831	3.6322	83.7615	29296.02	341.34	0.0052990
1A	0.1503736032	4.0919	94.3609	33003.19	303.00	0.1151881
1A	0.1604611904	4.3664	100.6909	35217.16	283.95	0.0642511
1A	0.1639026399	4.4600	102.8505	35972.47	277.99	0.0387898
1A	0.1674156888	4.5556	105.0549	36743.50	272.16	0.0272288
1A	0.1715636247	4.6685	107.6578	37653.86	265.58	0.3803788
1A	0.1762063273	4.7948	110.5712	38672.82	258.58	0.0251770
1A	0.1827763252	4.9736	114.6939	40114.77	249.28	0.0407952
1A	0.1845774269	5.0226	115.8241	40510.06	246.85	0.0561786
1A	0.1861743313	5.0661	116.8262	40860.54	244.73	0.2318798
1A	0.1932798030	5.2594	121.2849	42420.01	235.74	0.0004256
1A	0.1940826517	5.2813	121.7887	42596.22	234.76	0.0972018
1A	0.1972304596	5.3669	123.7640	43287.08	231.02	0.0563612
1A	0.2022102414	5.5024	126.8889	44380.02	225.33	0.0029559
1A	0.2043994719	5.5620	128.2626	44860.50	222.91	0.0071352

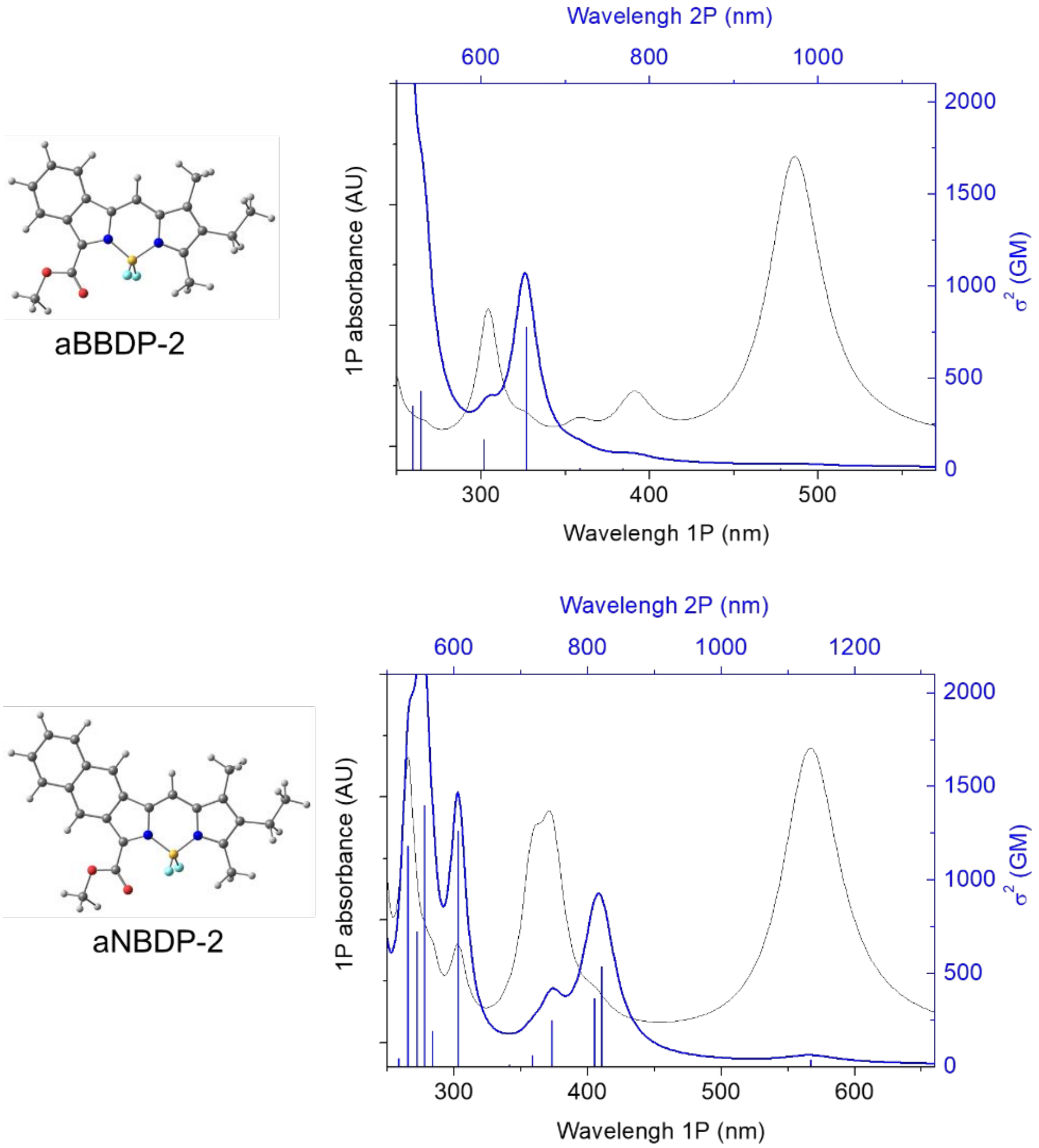

Figure S19: 1PA (black lines) and 2PA spectra (blue bars) of **aBBDP-2** and **aNBDP-2** computed using TDDFT/SOS methodology.

6. Crystallographic Data

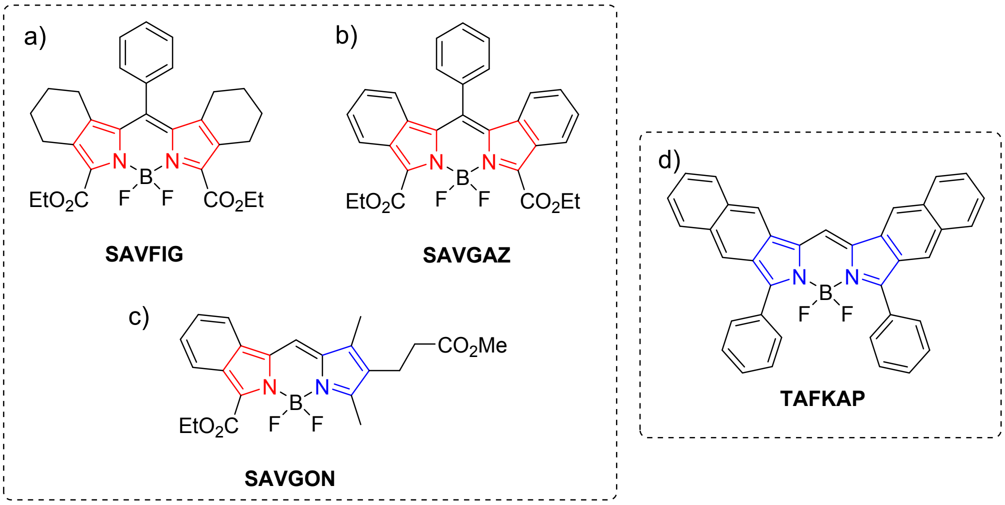

Figure S20: Structures of the compounds a) SAVFIG b) SAVGAZ and c) SAVGON reported by Uppal et al.[12] and d) TAFKAP reported by Yamazawa et al.[21]

Crystals were grown by dissolving the compounds in a DCM-MeOH mixture and allowing for slow evaporation over time. Data for samples was collected on a Bruker Kappa Apex Duo using CuKα (λ = 1.54178 Å) radiation. Samples were mounted on a MiTeGen microloop and data collected at 100(2) K using an Cobra (Bruker Kappa Apex Duo) low temperature device. Data were collected by using omega and phi scans and were corrected for Lorentz and polarization effects using APEX.[22a] Absorption corrections were applied using SADABS.[22b]

The structure was solved with the XT structure solution program,[22c,d] using the intrinsic phasing solution method and refined against ∣ F2 ∣ with XL using least squares minimization[22e] within OLEX2.[22f] Hydrogen atoms, unless specified, were placed in geometrically calculated positions and refined using a riding model. Molecular graphics were generated using OLEX2. Details on data collection and refinement are given in Table S4.

Refinement details for aBDP1 (TCD2044)

Refined as a rotational twin using the TWINS routine in OLEX2 with twin law: 0.996, -0.012, 0.016, 0.2, -1.001, 0.002, 0.599, -0.004, -0.995 and refined with HKLF5 data giving a twin ratio of 0.178(5). The disorder in the 6-membered ring was refined over two locations (88:12% occupancy) using geometrical (SADI) and displacement (SIMU) restraints.

Refinement details for aNBDP1 (TCD2066)

Modelled as a rotational twin using PLATON with a 2-axis rotation around (8, -1, 0) [1, 0, 0], with Angle () [] = 0.42 Deg, (1.000, 0.000, 0.008, -0.250, -1.000, -0.001, 0.000, 0.000, -1.000). Refined twin ratio 0.354(9).

Refinement details for aNBDP1·Tol (TCD2047)

Main molecule lies on a mirror plane. End carbon of the ethoxy group is disordered across the plane (50%) and also the terminal phenyl group (C16-C19, 50%). Modelled with displacement restraints (SIMU, RIGU). The toluene molecule is disordered over inversion symmetry and is modelled at 25% occupancy in the ASU using a rigid group and displacement constraints (EADP). The final formula is 1 molecule of BODIPY to 0.5 toluene generated by symmetry.

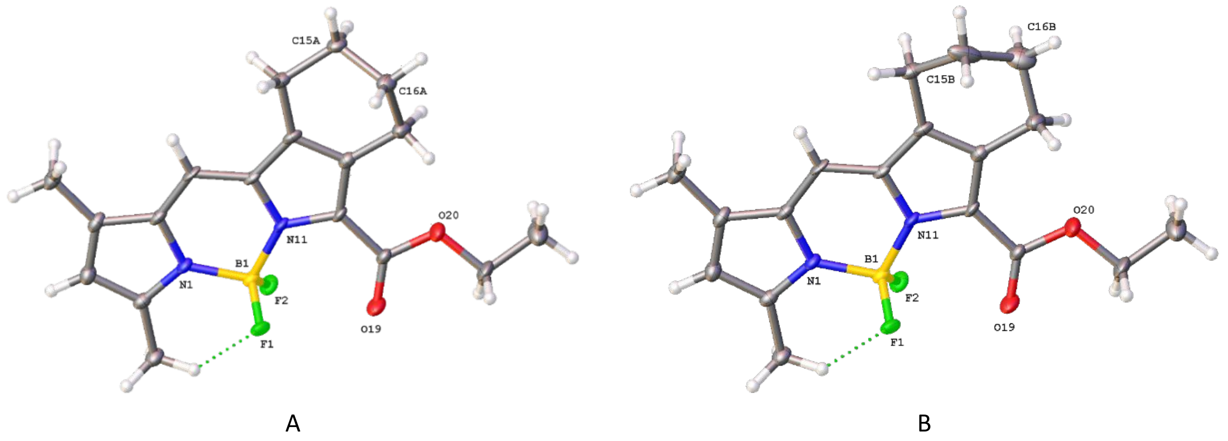

Figure S21: Individual representations of each disordered moiety in **aBDP-1** with (A) 88% occupied and (B) 12% occupied. Atomic displacement shown at 50% probability.

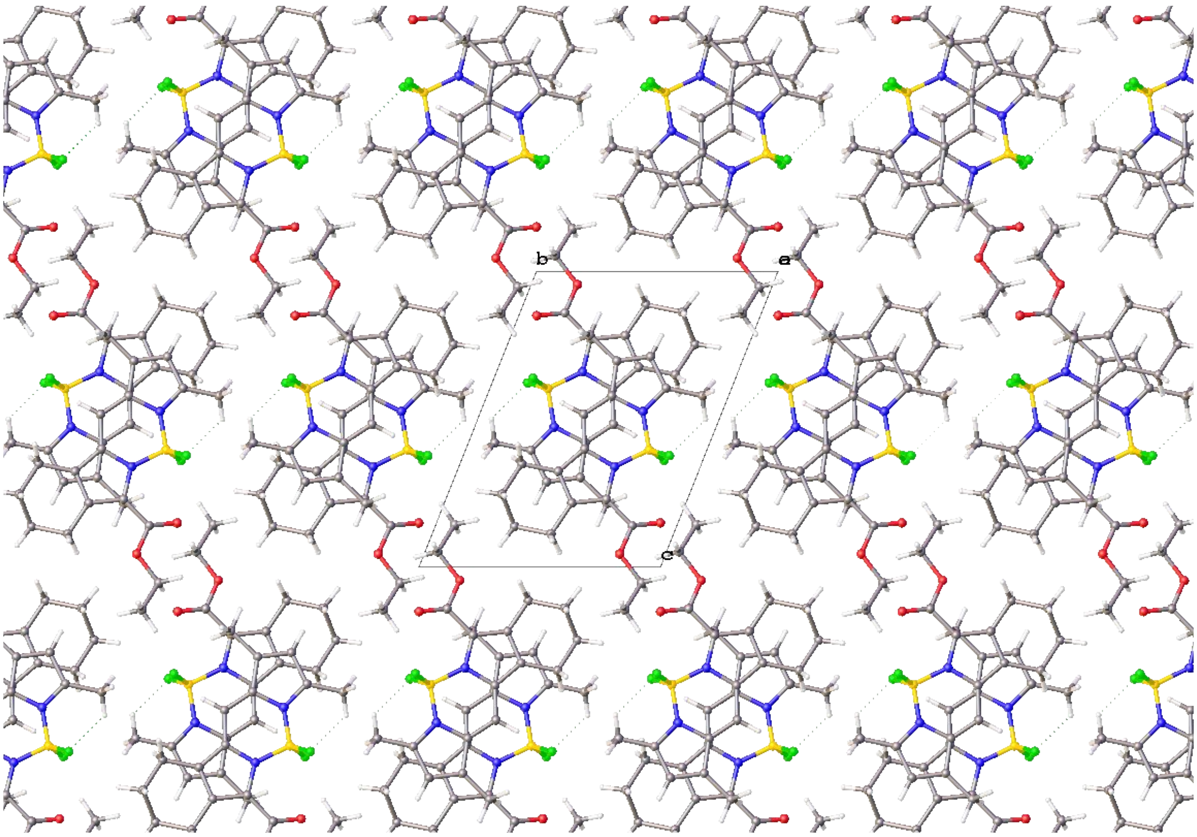

Figure S22: Schematic packing diagram of the majority occupied moiety in **aBDP-1**, viewed normal to the a-axis. Possible intramolecular hydrogen bonding is shown as a dotted line.

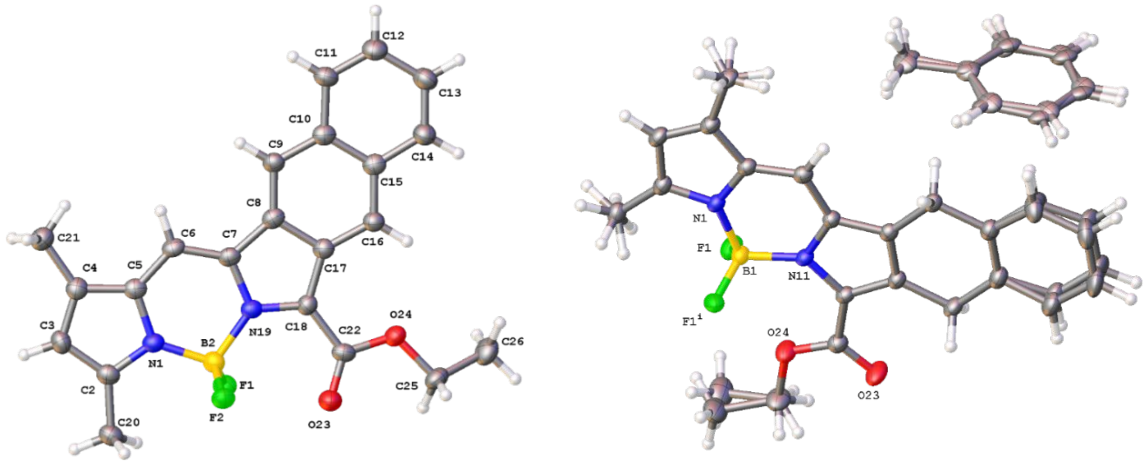

A B

Figure S23: A) Molecular structure of **aNBDP-1**. Atomic displacement shown at 50% probability. B) Molecular structure of **aNBDP1-1-Tol**, symmetry generated over a mirror plane (i = +X, -Y, +Z). The terminal carbon of the ethoxy group and also the terminal phenyl group (C16-C19) are disordered across the plane (50%). Atomic displacement shown at 50% probability and heteroatoms and symmetry unique carbon atoms labelled only.

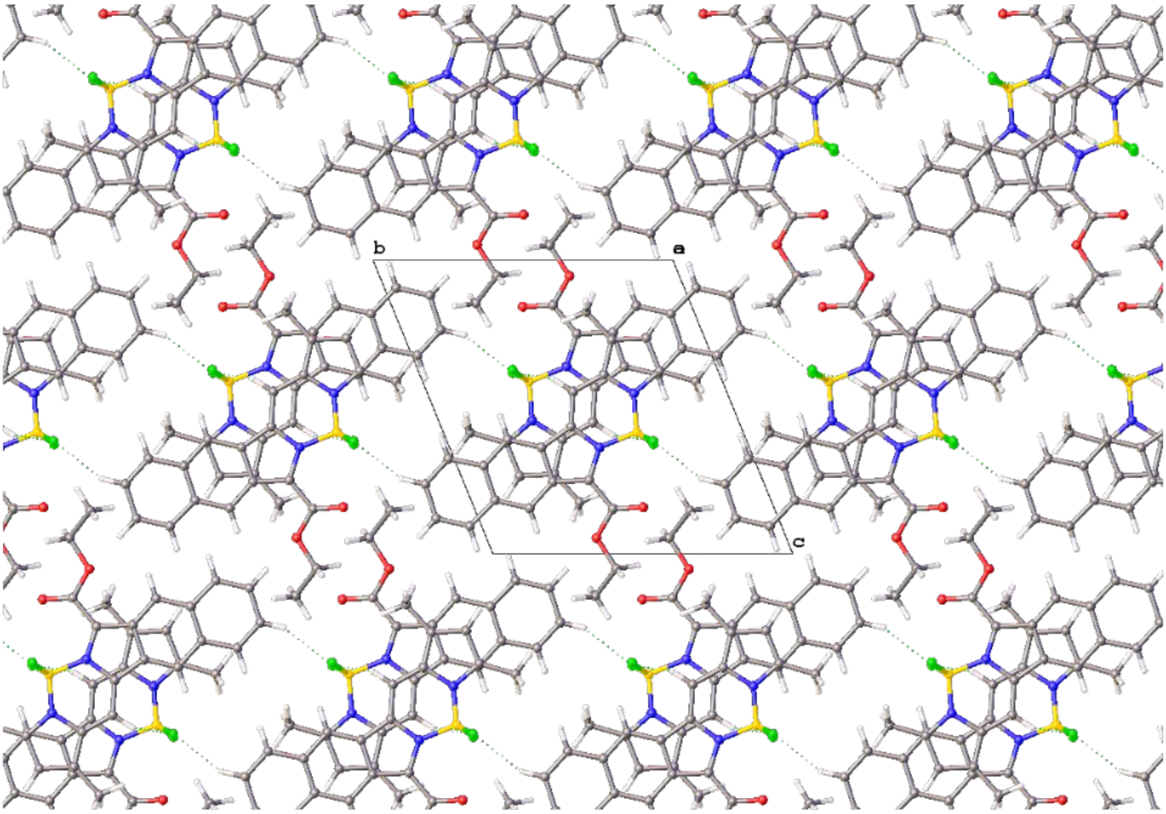

Figure S24: Schematic packing diagram of **aNDBP-1**, viewed normal to the a-axis. Dotted lines indicate intermolecular hydrogen bonding.

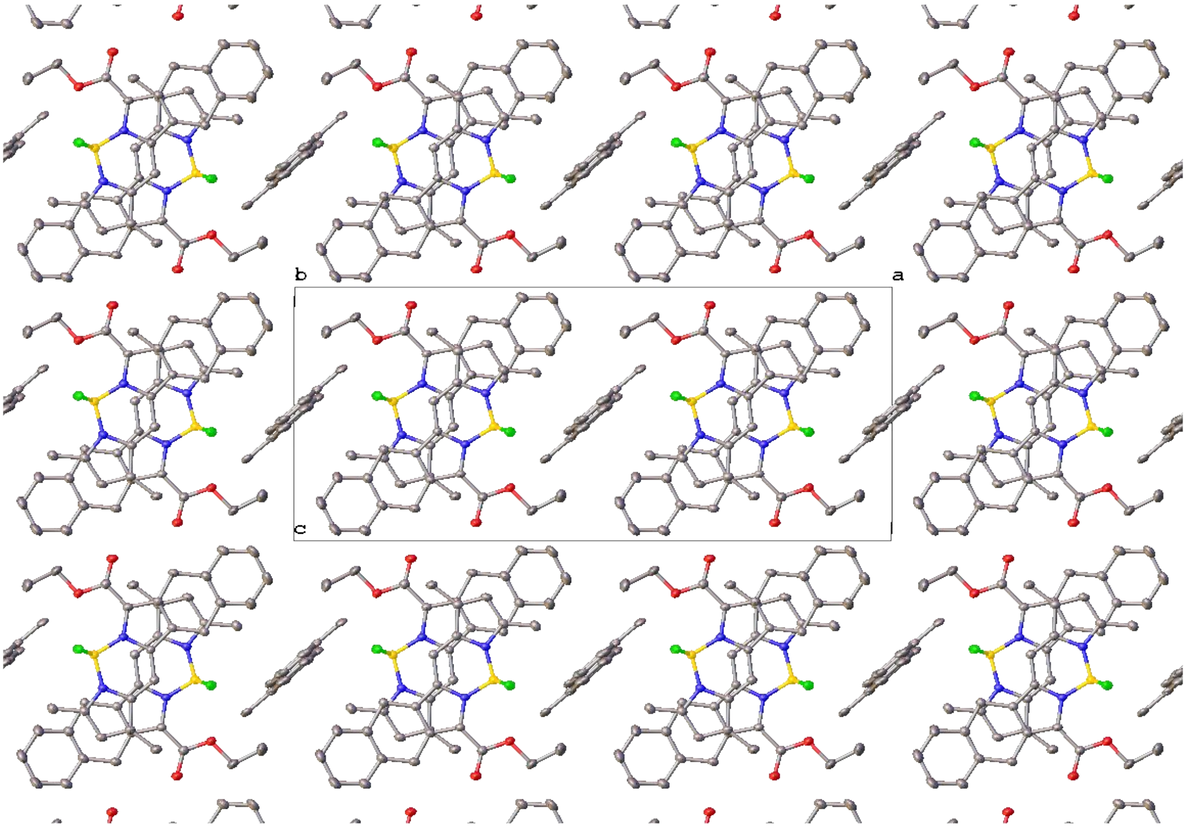

Figure S25: Schematic packing diagram of **aNBDP-1-Tol** viewed normal to the b-axis, showing the solvent channel. Hydrogen atoms omitted for clarity.

Table S4: Crystal data and refinement details.

Compound	**aBDP1**	**aNBDP1·Tol**	**aNBDP1**
CCDC number	2457666	2457664	2457665
Internal code	TCD2044	TCD2047	TCD2066
Empirical formula	C18H21BF2N2O2	C25.50H25BF2N2O2	C44H38B2F4N4O4
Formula weight	346.18	440.28	784.40
Temperature [K]	100(2)	100(2)	100(2)
Crystal system	triclinic	monoclinic	triclinic
Space group (number)	$P\bar{1}$ (2)	$C2/m$ (12)	$P\bar{1}$ (2)
a [Å]	7.1416(4)	27.4133(11)	6.7512(5)
b [Å]	9.7368(5)	6.8908(3)	11.8970(8)
c [Å]	12.9463(7)	11.6241(5)	12.5169(8)
α [°]	67.920(2)	90	112.126(5)
β [°]	81.129(2)	90.0596(15)	90.424(6)
γ [°]	85.624(2)	90	93.926(6)
Volume [Å3]	824.07(8)	2195.79(16)	928.52(11)
Z	2	4	1
ρcalc [gcm−3]	1.395	1.332	1.403
μ [mm−1]	0.879	0.783	0.858
F(000)	364	924	408
Crystal size [mm3]	0.276×0.154×0.072	0.332×0.135×0.032	0.282×0.111×0.027
Crystal colour	clear red	red	blue
Crystal shape	block	block	plate
Radiation	Cu $K\alpha$ (λ=1.54178 Å)	Cu $K\alpha$ (λ=1.54178 Å)	Cu$K\alpha$ (λ=1.54178 Å)
2Θ range [°]	7.44 to 139.98 (0.82 Å)	6.45 to 139.79 (0.82 Å)	7.63 to 135.32 (0.83 Å)
Index ranges	-8 ≤ h ≤ 8<br>-10 ≤ k ≤ 11<br>0 ≤ l ≤ 15	-33 ≤ h ≤ 33<br>-8 ≤ k ≤ 7<br>-14 ≤ l ≤ 14	-8 ≤ h ≤ 7<br>-14 ≤ k ≤ 14<br>-12 ≤ l ≤ 14
Reflections collected	3032	23742	3261
Independent reflections	3032<br>Rint = 0.0687<br>Rsigma = 0.0372	2264<br>Rint = 0.0508<br>Rsigma = 0.0236	3261<br>Rint = 0.0756<br>Rsigma = 0.0785
Completeness	98.2 %	100.0 %	97.5 %
Data / Restraints / Parameters	3032/44/249	2264/72/203	3261/0/266
Goodness-of-fit on F2	1.086	1.191	1.064
Final R indexes [I≥2σ(I)]	R1 = 0.0694<br>wR2 = 0.1953	R1 = 0.0612<br>wR2 = 0.1478	R1 = 0.0894<br>wR2 = 0.2677
Final R indexes [all data]	R1 = 0.0734<br>wR2 = 0.2000	R1 = 0.0628<br>wR2 = 0.1487	R1 = 0.1199<br>wR2 = 0.2918
Largest peak/hole [eÅ−3]	0.43/-0.29	0.45/-0.31	0.50/-0.43

7. References

[1] R. F. Kubin, A. N. Fletcher *J. Lumin.,* **1982**, *27*, 455–462.

[2] O.S. Finikova, A.V. Cheprakov, I.P. Beletskaya, P.J. Carroll, S.A. Vinogradov, *J. Org. Chem.*, **2004**, *69*, 522-535.

[3] O.S. Finikova, S.E. Aleshchenkov, R.P. Brinas, A.V. Cheprakov, P.J. Carroll, S.A. Vinogradov, *J. Org. Chem.*, **2005**, *70*, 4617-4628.

[4] T. Mikulchyk, S. Karuthedath, C.S.P. De Castro, A.A. Buglak, A. Sheehan, A. Wieder, F. Laquai, I. Naydenova, M.A. Filatov, *J. Mater. Chem. C*, **2022**, *10*, 11588-11597.

[5] P. Kancharla, J. X. Kelly, K.A. Reynolds, *J. Med. Chem.*, **2015**, *58*, 7286–7309.

[6] S. Ito, M. Akaki, Y. Shinozaki, Y. Iwabe, M. Furuya, M. Tobata, M. Roppongi, T. Sato, N. Itoh, T. Oba, *Tetrahedron Letters*, **2017**, *58,* 1338-1342.

[7] M. J. Kim, S. M. Gaube, M. H. R. Beh, C. D. Smith, A. Thompson, *RSC. Adv.,* **2019**, *9*, 31773-31780.

[8] M.A. Filatov, T. Mikulchyk, M. Hodée, M. Dvoracek, V.N.K. Mamillapalli, A. Sheehan, C. Newman, S.M. Borisov, D. Escudero, I. Naydenova, *J. Mater. Chem. C*, **2025**, doi: 10.1039/D4TC04850D

[9] B. R. Groves, T. S. Cameron, A. Thompson, *Org. Biomol. Chem.,* **2017**, *15,* 7925.

[10] Nuraneeva, E. N.; Guseva, G. B.; Antina, E. V.; V'yugin, A. I. *J. Photochem. Photobiol. A: Chem.* **2023**, *439*, 114611–114611.

[11] Wu, L.; Burgess, K. *Chem. Commun.* **2008**, *40*, 4933–4933.

[12] T. Uppal, Dr. X. Hu, Dr. F. R. Fronczek, S. Maschek, P. Bobadova-Parvanova, M. G. H. Vicente, *Chem. Eur. J.* **2012,** *18,* 3893- 3905.

[13] A.A. Buglak, A. Charisiadis, A. Sheehan, C.J. Kingsbury, M.O. Senge, M.A. Filatov, *Chem. Eur. J.*, **2021**, *27*, 9934.

[14] Y. Zhao, R. Duan, J. Zhao, C. Li, Spin–Orbit Charge Transfer Intersystem Crossing in Perylenemonoimide–Phenothiazine Compact Electron Donor–Acceptor Dyads. *Chem. Commun.* **2018**, *54* (87), 12329–12332.

[15] F. Wilkinson, W.P. Helman, A.B. Ross, *J. Phys. Chem. Ref. Data* **1993**, *22* (1), 113–262.

[16] M. Drobizhev, R.S. Molina and T.E. Hughes, *Bio-Protocol* **2020**, *10*, e3498.

[17] Dalangin, R. *et al. Nat. Commun.* **2025**, *16*, 3318.

[18] A. A. Granovsky, *FireFly*, version 8.2.0, http://classic.chem.msu.su/gran/firefly/index.htm

[19] M. J. Frisch, G. W. Trucks, H. B. Schlegel, G. E. Scuseria, M. A. Robb, J. R. Cheeseman, G. Scalmani, V. Barone, G. A. Petersson, H. Nakatsuji, X. Li, M. Caricato, A. V. Marenich, J. Bloino, B. G. Janesko, R. Gomperts, B. Mennucci, H. P. Hratchian, J. V. Ortiz, A. F. Izmaylov, J. L. Sonnenberg, D. Williams-Young, F. Ding, F. Lipparini, F. Egidi, J. Goings, B. Peng, A. Petrone, T. Henderson, D. Ranasinghe, V. G. Zakrzewski, J. Gao, N. Rega, G. Zheng, W. Liang, M. Hada, M. Ehara, K. Toyota, R. Fukuda, J. Hasegawa, M. Ishida, T. Nakajima, Y. Honda, O. Kitao, H. Nakai, T. Vreven, K. Throssell, J. A. Montgomery Jr., J. E. Peralta, F. Ogliaro, M. J. Bearpark, J. J. Heyd, E. N. Brothers, K. N. Kudin, V. N. Staroverov, T. A. Keith, R. Kobayashi, J. Normand, K. Raghavachari, A. P. Rendell, J. C. Burant, S. S. Iyengar, J. Tomasi, M. Cossi, J. M. Millam, M. Klene, C. Adamo, R. Cammi, J. W. Ochterski, R. L. Martin, K. Morokuma, O. Farkas, J. B. Foresman and D. J. Fox, *Gaussian 16, Revision A.03*, Gaussian, Inc., Wallingford CT, **2016**.

[20] S. R. Allu, L. Ravotto, T. Troxler, S. A. Vinogradov, *J. Phys. Chem. A* **2021**, *125* (14), 2977–2988.

[21] S. Yamazawa, M. Nakashima, Y. Suda, R. Nishiyabu and Y. Kubo, *J. Org. Chem.,* **2016**, *81* (3), 1310–1315.

[22] (a) Bruker, APEX3 V2017.3-0, Bruker AXS Inc., Madison, Wisconsin, (USA), 2017; (b) L. Krause, R. Herbst-Irmer, G. M. Sheldrick, D. Stalke, *J. Appl. Crystallogr.* **2015**, *48*, 3–10; (c) G. M. Sheldrick, *Acta Crystallogr.* **2015**, *A71*, 3–8; (d) G. M. Sheldrick, *Acta Crystallogr.* **2015**, *C71*, 3–8; (e) O. V. Dolomanov, L. J. Bourhis, R. J. Gildea, J. A. K. Howard, H. Puschmann, *J. Appl. Crystallogr.* **2009**, *42*, 339–341; (f) S. M. Cohen, S. R. Halper, *Inorg. Chim. Acta* **2002**, *341*, 12–16; (g) A. L. Spek, *Acta Crystallogr.* **2015**, *C71*, 9–18.
